# Unique organization and unprecedented diversity of the *Bacteroides (Pseudobacteroides) cellulosolvens* cellulosome system

**DOI:** 10.1186/s13068-017-0898-6

**Published:** 2017-09-07

**Authors:** Olga Zhivin, Bareket Dassa, Sarah Moraïs, Sagar M. Utturkar, Steven D. Brown, Bernard Henrissat, Raphael Lamed, Edward A. Bayer

**Affiliations:** 1 Department of Biomolecular Sciences, Weizmann Institute of Science, Rehovot, Israel; 2 Graduate School of Genome Science and Technology, University of Tennessee, Knoxville, TN 37919 USA; 3 BioEnergy Science Center, Oak Ridge, TN USA; 4 Biosciences Division, Energy and Environment Directorate, Oak Ridge National Laboratory, Oak Ridge, TN USA; 5 Architecture et Fonction des Macromolécules Biologiques, Aix-Marseille University and CNRS, Marseille, France; 6 Department of Molecular Microbiology and Biotechnology, Tel Aviv University, Ramat Aviv, Israel

**Keywords:** Cohesin, Dockerin, Scaffoldin, Cellulolytic bacteria, CBM, Cellulases, Glycoside hydrolases

## Abstract

**Background:**

*(Pseudo) Bacteroides cellulosolvens* is an anaerobic, mesophilic, cellulolytic, cellulosome-producing clostridial bacterium capable of utilizing cellulose and cellobiose as carbon sources. Recently, we sequenced the *B. cellulosolvens* genome, and subsequent comprehensive bioinformatic analysis, herein reported, revealed an unprecedented number of cellulosome-related components, including 78 cohesin modules scattered among 31 scaffoldins and more than 200 dockerin-bearing ORFs. In terms of numbers, the *B. cellulosolvens* cellulosome system represents the most intricate, compositionally diverse cellulosome system yet known in nature.

**Results:**

The organization of the *B. cellulosolvens* cellulosome is unique compared to previously described cellulosome systems. In contrast to all other known cellulosomes, the cohesin types are reversed for all scaffoldins i.e., the type II cohesins are located on the enzyme-integrating primary scaffoldin, whereas the type I cohesins are located on the anchoring scaffoldins. Many of the type II dockerin-bearing ORFs include X60 modules, which are known to stabilize type II cohesin–dockerin interactions. In the present work, we focused on revealing the architectural arrangement of cellulosome structure in this bacterium by examining numerous interactions between the various cohesin and dockerin modules. In total, we cloned and expressed 43 representative cohesins and 27 dockerins. The results revealed various possible architectures of cell-anchored and cell-free cellulosomes, which serve to assemble distinctive cellulosome types via three distinct cohesin–dockerin specificities: type I, type II, and a novel-type designated R (distinct from type III interactions, predominant in ruminococcal cellulosomes).

**Conclusions:**

The results of this study provide novel insight into the architecture and function of the most intricate and extensive cellulosomal system known today, thereby extending significantly our overall knowledge base of cellulosome systems and their components. The robust cellulosome system of *B. cellulosolvens,* with its unique binding specificities and reversal of cohesin–dockerin types, has served to amend our view of the cellulosome paradigm. Revealing new cellulosomal interactions and arrangements is critical for designing high-efficiency artificial cellulosomes for conversion of plant-derived cellulosic biomass towards improved production of biofuels.

**Electronic supplementary material:**

The online version of this article (doi:10.1186/s13068-017-0898-6) contains supplementary material, which is available to authorized users.

## Background

Cellulosic biomass and waste are raw materials of great abundance, and its deconstruction conversion to soluble sugars is an important resource within the context of production of biofuels and valuable chemicals [1, 2]. Some anaerobic cellulolytic bacterial strains have developed the cellulosome, an efficient enzymatic strategy to utilize cellulosic biomass as a major carbon source. One of the major advantages of cellulosome-producing bacteria is their ability to degrade different types of carbohydrates present in various types of biomass [3]. The organization of enzymes into a cellulosome serves to concentrate them physically and position them in suitable orientation, both with respect to each other and to the cellulosic substrate, for efficient decomposition of the recalcitrant insoluble substrate [4]. Moreover, the fact that the complex is both attached to the substrate and to the cell results in minimal diffusion loss of enzymes and hydrolytic products, and precludes product-mediated feedback inhibition of the cellulolytic enzymes. The cellulosomal enzymes are incorporated into the complex via their resident dockerin module and interact specifically with the cohesin modules of a structural scaffoldin subunit [3–6]. The scaffoldin subunit can selectively integrate enzymes or additional scaffoldin subunits into the cohesive complex via specific and high-affinity cohesin–dockerin interactions, which thus determine overall cellulosome architecture [7–10].

Cohesins and dockerins have been classified traditionally into types (I, II, and III) based on sequence similarity [3, 11, 12]. Primary scaffoldins, the backbone of the cellulosomal complex, have thus far been demonstrated to contain multiple type I cohesins, each of which interacts with a type I dockerin harbored by each cellulosomal enzyme [13–15]. The primary scaffoldin may contain a dockerin module that interacts with the cohesins of an adaptor and/or anchoring scaffoldin, thereby allowing the expansion of the cellulosome complex by integration of multiple enzymes and/or the attachment of the cellulosome to the cell surface [16–18]. These scaffoldin assemblies are generally mediated by type II cohesins and dockerins that are located on the adaptor or anchoring scaffoldins [9, 18]. The anchoring scaffoldins can contain one or more cohesins and anchor the cellulosome complexes to the cell surface via a surface-layer homology (SLH) domain [19, 20]. The cohesin–dockerin interactions are considered to be species- and/or type-specific, although some cross-species interactions have been observed [21].

In this context*, (Pseudo) Bacteroides cellulosolvens* is an anaerobic, mesophilic, cellulolytic bacterium that was isolated from a methanogenic cellulose-enrichment culture of municipal sewage sludge [22]. This bacterium produces an extracellular multi-enzyme cellulosome complex for efficient degradation of plant cell wall polysaccharides and cellulosic wastes [23] and is capable of utilizing cellulose or cellobiose as a sole carbon source [22]. Originally termed *Bacteroides cellulosolvens*, the bacterium was subsequently found to be phylogenetically related to the clostridial assemblage [24] and more recently reclassified as *Pseudobacteroides cellulosolvens* [25]. Earlier work reported two major scaffoldins in *B. cellulosolvens* [26] and the cellulolytic potential of the bacterium [22, 27]. The two proteins, a primary scaffoldin and an anchoring scaffoldin, were the largest yet described, bearing 11 and 10 cohesins, respectively [28, 29]. Recently, the *B. cellulosolvens* genome was sequenced to near-completion [30] allowing comprehensive bioinformatic studies that will represent a milestone in current research on this bacterium. Therefore, in this work, we explored the architectural and functional aspects of the cellulosome of *B. cellulosolvens*, and in particular the cohesin–dockerin specificities of interactions between different scaffoldin and enzymatic modules. Its large range of cellulosomal components was revealed, and we demonstrated binding activity and specificity of selected cohesin and dockerin modules, thus revealing overall cellulosome architecture in this intriguing cellulosome-producing bacterium.

## Methods

### Anaerobic fermentation of *Bacteroides cellulosolvens*

*Bacteroides cellulosolvens* ATCC 35603 was grown under anaerobic conditions essentially as described by Murray et al. [22] with either cellobiose (CB, Sigma Chem. Co. St. Louis, MO) or microcrystalline cellulose (MCC, Avicel, E. Merck, Darmstadt, Germany) as carbon and energy source. *B. cellulosolvens* cell lysates were prepared using PopCulture Reagent (Novagen Inc, Darmstadt, Germany), as described by Slutzki et al. [31].

### Fractionation of high-molecular-weight complexes

The spent growth medium of *B. cellulosolvens* cells, grown on either CB or MCC, was concentrated 100-fold and subjected to gel-filtration chromatography on a Superose 6 gel-filtration column (GE Healthcare) as described earlier [32]. The two resultant peaks (I and II) were pooled and concentrated using a Vivaspin concentrator (50-kDa cutoff; Sartorius Stedim Biotech GmbH, Göttingen, Germany).

### Bioinformatics analysis

Blastp searches were performed against predicted *B. cellulosolvens* proteins, using deduced amino acid sequences of the known cohesin and dockerin modules as queries [16, 17, 33]. Hits above an *E*-value of 10^−4^ were examined individually, by searching for characteristic sequence features. For example, for dockerin modules, we searched for two Ca^2+^-binding repeats, putative helices and linker regions. Multiple sequence alignments were created using the Clustal Omega server [http://www.ebi.ac.uk/Tools/msa/clustalo/]. Phylogenetic trees were generated by iTOL version 3 [http://itol.embl.de/] according to the “One Click” Phylogeny analysis tool [http://www.phylogeny.fr/simple_phylogeny.cgi]. Signal peptide sequences were predicted using the SignalP server [http://www.cbs.dtu.dk/services/SignalP/]. Amino acid sequence logos were performed using the WebLogo3 application, version 3.5.

### Annotation of dockerin-containing enzymes

The proteins were annotated using the carbohydrate-active enzymes database (CAZy) http://www.cazy.org/ [34]. The analysis was based on sequence conservation between catalytic modules, and the different catalytic modules were sorted into different families.

### Cloning and expression plasmid cassettes

The XynDoc gene cassette consists of xylanase T6 from *Geobacillus stearothermophilus* with an N-terminal His-tag cloned into plasmid pET9d (Novagen Inc., Madison, WI, USA), into which a dockerin-encoding sequence was introduced between the KpnI and BamHI restriction sites of the plasmid [35]. The CBM-Coh gene cassette consists of a family CBM3 (family 3 carbohydrate-binding module) from the *Clostridium thermocellum* CipA scaffoldin cloned into plasmid pET28a (Novagen Inc., Madison, WI, USA), into which a cohesin gene was introduced between BamHI and XhoI restriction sites of the plasmid [35, 36].

### Polymerase chain reaction (PCR)

An expanded high-fidelity PCR system (Boehringer Mannheim) was used in all PCRs. PCR was performed using a Mastercycler personal instrument (Eppendorf, Hamburg, Germany), programed as follows: a 3-min predenaturation step at 95 °C was followed by 30 cycles comprising a 45-s denaturation step at 94 °C, an annealing step of 30 s at 50–60 °C (depending on the primer), and an extension step at 72 °C for 1 min. The primers used for the cloning of 43 cohesins and 27 dockerins are listed in Additional file 1: Table S1.

### Cloning procedure

PCR products were purified and double digested at 37 °C for 15–30 min with FastDigest restriction enzymes (Thermo Scientific) and ligated into the desired plasmid. Positive clones were verified by sequencing.

### Protein expression

The pET28a cassette containing the CBM-Coh fusion proteins and the pET9d cassette containing the XynDoc fusion proteins were transformed into *Escherichia coli* BL21 (DE3) strains and plated onto LB-kanamycin plates. For each plate, 4–5 ml of Luria–Bertani broth (LB) were added in order to resuspend the cells. The resuspended cells were added to 1 l of LB with 50 µg/ml kanamycin and 2 mM CaCl_2_ and were grown for 2 h at 37 °C to A_600_ ≈ 0.8–1. Protein expression was induced by adding isopropyl-1-thio-β-D-galactoside (IPTG) (Fermentas UAB, Vilnius, Lithuania) in a final concentration of 0.2 mM, and the growth was continued in 16 °C for 16 h. Cells were harvested by centrifugation at 5000 rpm for 15 min.

### Purification of CBM-containing proteins

The supernatant fluids of the cohesin-containing proteins (fused to a CBM tag, both for increased solubility and for affinity purification) were added to 2 g of preswollen cellulose gel macroporous beads (IONTOSORB, Usti nad Labem, Czech Republic) and incubated for 1 h with rotation at 4 °C. The mixture was then loaded onto a column, and washed with 100 ml of Tris-buffered saline (TBS: 13.7 mM NaCl, 0.27 mM KCl, 2.5 mM Tris, pH 7.4) brought to 1 M NaCl, and then washed with 100 ml TBS. Three 5-ml elutions of 1% triethanolamine (TEA) were then collected, protease-inhibitor cocktail was added. The fractions were subjected to SDS-PAGE in order to assess protein purity.

### Purification of Xyn-containing and His-tagged proteins

The supernatant fluids containing the dockerin-bearing proteins were mixed with 4 ml Ni–NTA, for 1 h on a 20-ml Econo-pack column, on a rotator at 4 °C (batch purification system). The column was then washed by gravity flow with 100 ml wash buffer (TBS, 15 mM imidazole). Elution was performed first using 100 mM imidazole, followed by 250 mM imidazole. Fractions (2 ml) were collected and were run on SDS-PAGE. The fractions containing relatively pure proteins were pooled, and CaCl_2_ (10 mM), as well as protease-inhibitor cocktail was added.

### Protein concentration and storage

Protein concentration was evaluated by absorbance at 280 nm, based on the extinction coefficients derived from the known composition of amino acids of each protein. Extinction coefficients were calculated using the ExPASy ProtParam tool http://web.expasy.org/protparam/. Some proteins were concentrated by Amicon ultra concentrators (Millipore, Ireland), and stored at −20 °C in 50% (vol/vol) glycerol.

### ELISA-based affinity assay

The standard ELISA procedure was performed as described previously [35]. Representative cohesin and dockerin modules were selected and expressed using one of the two cassettes described above. In this manner, we cloned 43 CBM-fused cohesins and 27 as Xyn-fused dockerins (13 from the scaffoldins and 14 from the putative enzymes). The 96-well ELISA plates (Nunc, A/S, Roskilde, Denmark) were coated with the fusion proteins CBM-Cohs or full-length scaffoldins at a concentration of 1–10 µg/ml, and variable concentrations of Xyn–Docs (0.001–1000 ng/ml) were used to detect specific cohesin–dockerin interactions. Interactions with the Xyn–Doc fusion proteins were examined immunochemically by using anti-xylanase primary antibody and HRP-labeled secondary antibody. The experiments were performed three times in duplicate.

For cell lysate-based ELISA, the 96-well ELISA plates were coated with cellobiose-grown *B. cellulosolvens* cell lysate, and graded concentrations of the desired Xyn–Docs were used to examine cohesin–dockerin interactions.

Absorbance was plotted as a function of Xyn–Doc fusion proteins concentration. For comparative purposes, the reference concentration of a Xyn–Doc standard that generates a maximum response was used in order to normalize the data as a relative binding of maximum response, as described earlier [35]. The results were presented as a heatmap (iTOL version 3, http://itol.embl.de/), whereby each node is associated with multiple numerical values, which are displayed as a set of colored boxes. Dataset values are mapped to a color gradient corresponding to the binding strength.

### Xylan hydrolysis

Xylan activity assay was performed in triplicate in a total volume of 500 µl, containing 50 mM citrate buffer (pH 6.5), 12 mM CaCl_2_, 2 mM EDTA, and 25 µg/ml of purified cellulosome complex from *B. cellulosolvens*. Xylan degradation was assayed at a final concentration of 1% beechwood xylan (Sigma-Aldrich, Rehovot, Israel), for 1 h at 42 °C (according to predetermined optimal conditions for *B. cellulosolvens* cellulosome activity). The assay performed for the purified *C.* *thermocellum* cellulosome was incubated at 70 °C (the optimal temperature for *C.* *thermocellum* cellulosome activity). The tubes were incubated under shaking (400 rpm), and the reaction was terminated by flash-cooling the tubes on ice. The tubes were centrifuged (14,000 rpm, 5 min), and 100 µl of the supernatant was transferred into 150 µl dinitrosalicylic acid (DNS) solution. The tubes were boiled for 10 min at 100 °C, and absorbance was measured at 540 nm in a plate reader. A glucose standard curve served to determine the amount of reducing sugars (in mM).

## Results

### The elaborate cellulosomal system of *B. cellulosolvens* revealed by bioinformatics

**Fig. 1:**
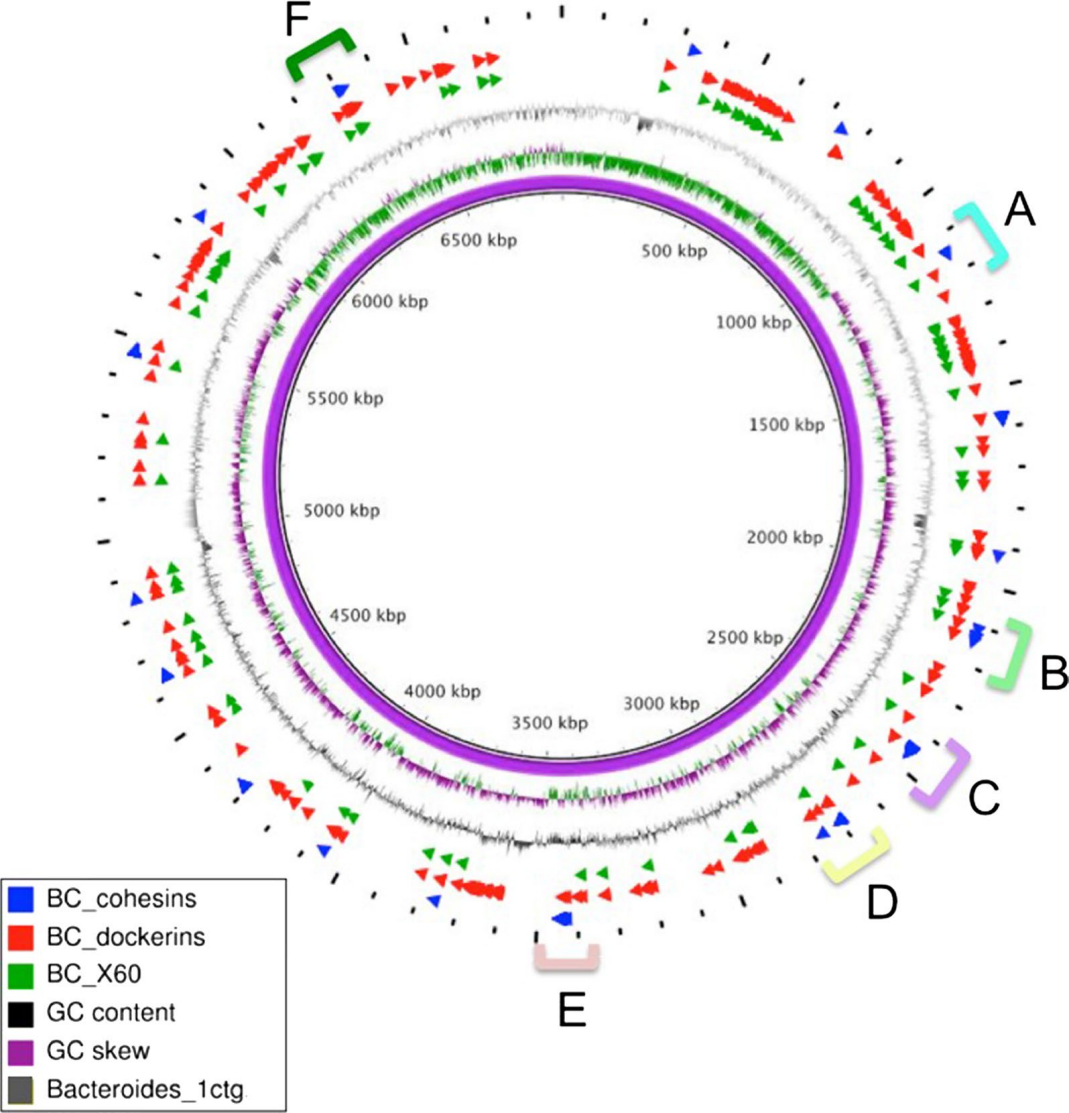
Circular genomic map of the *Bacteroides cellulosolvens* chromosome. The recently sequenced genome was assembled into a single large contig of 6,878,816 bp, translated into 5897 predicted proteins, and appears to be the largest among the known cellulolytic bacteria. The *innermost circles* represent the GC skew (*purple*/*green*) and GC content (*black*) of the sequence assembly. *Outer circles* show the location of cellulosomal elements based on Blastn homology. *Blue arrows* represent cohesin ORFs, grouped into *color-coded* clusters, marked by *letters A* to *F*. Dockerins, marked by *red arrows*, are scattered relatively evenly throughout the genome

**Fig. 2:**
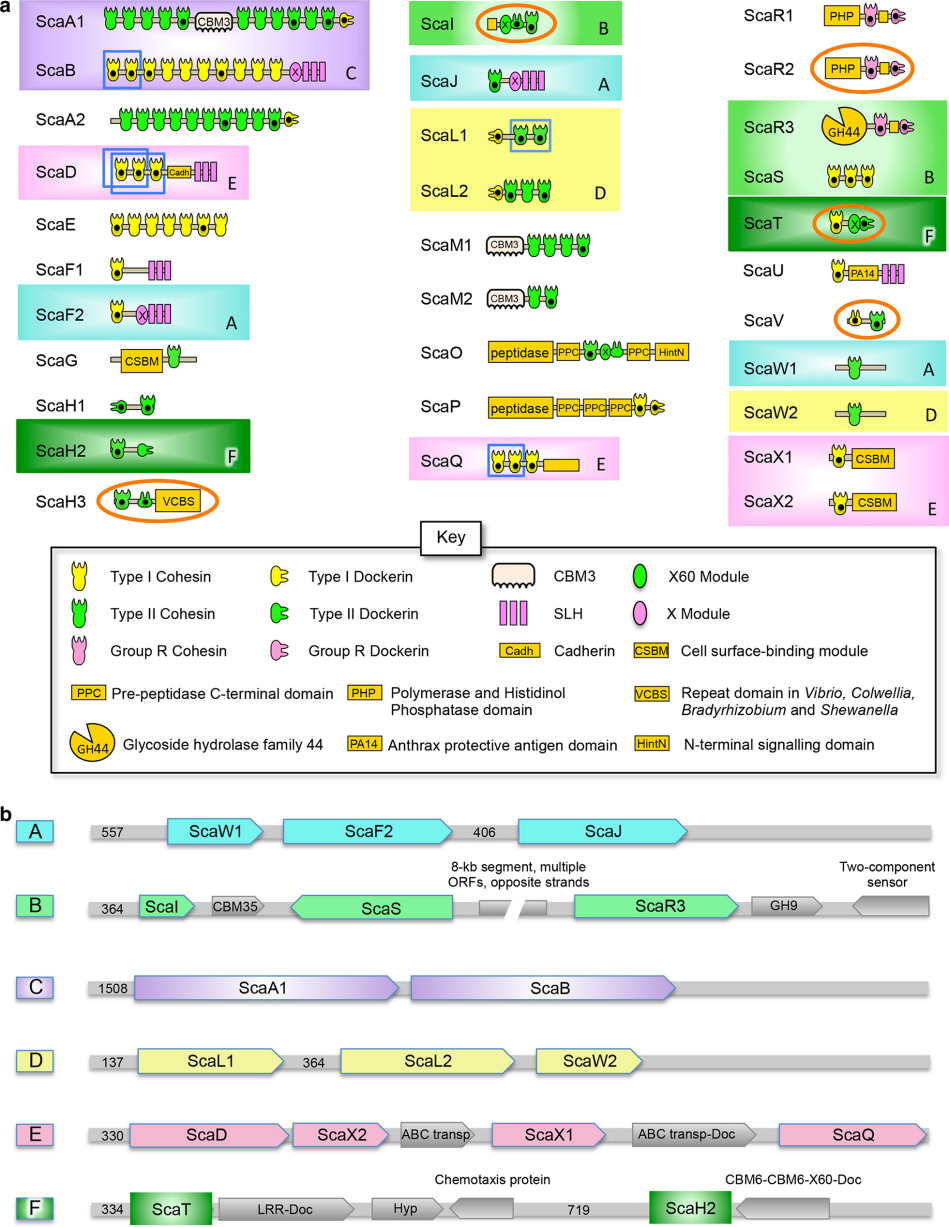
Scaffoldins and scaffoldin clusters of the *Bacteroides cellulosolvens* genome. **a** Schematic representation of the cohesin-borne scaffoldins. The 78 cohesins of *B. cellulosolvens* are classified into two main types: type I (33 modules) and type II (42 modules). In addition, group R was defined for cohesins from scaffoldins ScaR1-R3, whose sequences are notably different than those of the known types. We examined the conservation of the cohesin sequences within a given scaffoldin protein and among the different scaffoldins. *Dots* represent cohesin and dockerin modules that were selected, cloned, expressed, and examined experimentally. Clustered ORFs are enclosed by color-coded *rectangles* as defined in Fig. 1. *Orange ellipses* indicate scaffoldin ORFs that were fully expressed. *Blue squares* represent cohesins that were expressed in pairs/triplets. **b** Details of *sca* gene clusters. The figure represents the organization of the six *sca* gene clusters marked by *letters A* to *F* as designated in Fig. 1. *Color*-*coding* indicates the different ORFs within the specific cluster. The number in brackets within the regions indicates distances longer than 300 bp between the ORFs. Cluster A is a heterogeneous cluster including type I and type II cohesins where the scaffoldins possess only one cohesin. Cluster B includes ScaR3 located at a significant distance from the other two scaffoldins. The segment of 8-kb between ScaS and ScaR3 includes several ORFs of different function with relatively short distances (less than 300 bp) between them. Cluster C contains the two largest scaffoldins: ScaA1 is a primary scaffoldin that is bound by anchoring scaffoldin ScaB. The scaffoldins in Cluster D possess type II cohesins. ScaL1 and ScaL2 are very similar, except ScaL2 has an additional cohesin. The scaffoldins in Cluster E possess type I cohesins and together comprise the largest cluster with four scaffoldins. Cluster F is a heterogeneous cluster with two scaffoldins, each of which possesses a single cohesin and a single dockerin (see **a**)

We have recently sequenced the near-complete genome of *B. cellulosolvens* DSM 2933 (ATCC 35603), which appears to be the largest among the currently known cellulolytic bacteria (~6.9 Mbp) (Fig. 1) [30]. Detailed bioinformatics analysis revealed multiple cellulosomal components. In fact, this bacterium contains the largest number of cellulosomal components currently known. To delineate the mode by which the components may assemble into cellulosomal complexes remains an intriguing assignment [30]. We herein revealed 78 cohesin modules, scattered among 31 scaffoldins, and 212 dockerin-bearing ORFs, representing 197 putative carbohydrate-degrading enzymes [including assorted glycoside hydrolases (GHs), carbohydrate-binding modules (CBMs), carbohydrate esterases (CEs), polysaccharide lyases (PLs), and defined X-modules], and 15 dockerin-bearing scaffoldins (Fig. 2a). Almost half of the enzyme-borne type II dockerins (92 out of 212) possess an X60 module upstream of the dockerin sequence. As noted earlier for the then-discovered isolated *B. cellulosolvens* components [28, 29], in comparison to previously described cellulosome systems, the apparent roles of the *B. cellulosolvens* cohesins are curiously reversed, compared to all previously described cellulosomal components, in that the type II cohesins are located on the enzyme-binding primary scaffoldin, whereas the type I cohesins are located on the anchoring scaffoldin. In addition, significant numbers (17) of scaffoldin genes were found to be arranged in genomic clusters (Fig. 2b), whereas dockerin-containing genes were scattered more evenly throughout the genome (Fig. 1).

### Diversity of CAZy-associated cellulosomal enzymes

*Bacteroides cellulosolvens* was found to contain three times more dockerin-bearing proteins, as compared to other clostridia, such as *Clostridium cellulolyticum* (~60 dockerins), *C. thermocellum* (>70 dockerins), or *Clostridium clariflavum* (79 dockerins) [17, 37, 38]. The *Acidothermus cellulolyticus* genome contains 143 dockerin-containing ORFs [16]. The number (212) of dockerin-bearing ORFs in the *B. cellulosolvens* genome, however, is more comparable to those of *Ruminococcus flavefaciens* strains FD-1, 17, and 007c, which contain between 180 and 223 dockerins [33, 39, 40]. Table 1 presents the abundance of CAZy-associated modules (cellulosomal and non-cellulosomal) in the *B. cellulosolvens* genome. In general, about 50% of the *B. cellulosolvens* dockerins are associated with carbohydrate-active enzymes (GH, PL, CE). About 85 out of 212 dockerin-containing proteins were not associated with a defined CAZy module (Table 2).

**Table 1:** Comparison of CAZy-associated modules and CBMs in cellulosomal and non-cellulosomal proteins of *B. cellulosolvens*

Glycoside hydrolase	2	3	5	8	9	10	11	13	16	18	23	25	26	27	30	39
Dockerin-containing	–	1	8	4	33	7	5	–	4	1	–	–	4	1	3	–
Genome-wide	1	7	11	4	40	15	8	6	4	6	2	1	5	1	3	1

Glycoside hydrolase	43	44	48	51	53	57	62	63	67	74	75	81	94	95	Total
Dockerin-containing	9	2	3	–	–	–	1	–	–	1	–	1	–	–	88
Genome-wide	11	2	3	1	1	1	1	1	2	2	2	1	3	1	147

Polysaccharide lyase	1	3	8	9	11	12	Total
Dockerin-containing	1	–	–	1	1	–	3
Genome-wide	2	1	1	1	1	1	7

Carbohydrate esterase	1	2	3	4	6	7	8	12	15	Total
Dockerin-containing	3	1	4	3	3	–	1	2	2	19
Genome-wide	6	2	6	15	4	1	1	2	3	40

Carbohydrate-binding module	2	3	4	6	8	9	13	16	22	23	25	27	30	32
Dockerin-containing	–	16	14	14	1	1	3	–	–	1	–	1	2	2
Genome-wide	1	30	23	20	1	9	5	3	4	1	4	1	3	11

Carbohydrate-binding module	35	36	42	44	48	50	51	57	63	66	Total
Dockerin-containing	3	–	1	–	–	–	–	–	1	–	60
Genome-wide	8	1	2	1	6	15	1	1	2	1	154

**Table 2:**
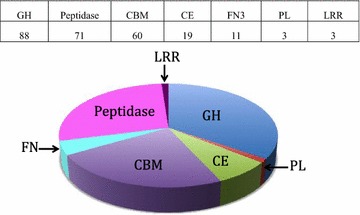
Predicted catalytic and non-catalytic modules associated with dockerins in *B. cellulosolvens*

The GH48 enzymes are known to be the definitive exoglucanase and quantitatively most abundant enzyme type, in all known cellulosomes [41, 42]. Remarkably, the *B. cellulosolvens* cellulosome contains three distinct GH48 enzymes in contrast to *A. cellulolyticus*, *C. thermocellum*, and *C. clariflavum* that contain only a single GH48 cellulosomal enzyme [17, 41]. As opposed to other known clostridial species that possess only one GH48 cellulosomal enzyme, in *B. cellulosolvens* all three GH48 possess a dockerin. The cellulase systems of other complex cellulosome-producing clostridia mentioned above also contain a second GH48 enzyme, but it bears a CBM3 rather than a dockerin and is thus not cellulosomal. One of the *B. cellulosolvens* GH48 enzymes (WP_050753099.1) was shown previously to bind a ScaA1 cohesin [26].

As reported earlier [22, 43, 44], *B. cellulosolvens* grows on cellulose and cellobiose as sole carbon sources but is also able to degrade xylan and has high xylanase activity in secreted and cell-associated fractions (Additional file 2: Figure S1). Recently, it was shown that *B. cellulosolvens* is a highly active lignocellulolytic microorganism able to efficiently digest cellulose, hemicellulose, and lignin together with *Clostridium stercorarium* [45]. Here, CAZy analysis revealed 147 GH modules, with a wide array of cellulolytic and hemicellulolytic enzymes, either cellulosomal (about 60% containing dockerins) or free enzymes (40%). Many of the cellulosomal and free enzymes (94 and 60, respectively) possess a CBM in addition to the definitive catalytic module(s), and in some cases more than one, thereby enabling extensive interaction with the lignocellulosic substrate.

Thirteen GH families in the genome are non-cellulosomal, suggesting that they could support biomass degradation of cellulose in free form or contribute to the degradation of distant or concealed carbohydrates. Interestingly, the percentage of dockerin-containing GHs (60%) in the *B. cellulosolvens* genome is similar to that of *C. thermocellum* and *A. cellulolyticus,* despite the superior number of enzymes in *B. cellulosolvens*. The GH9 family includes the highest number of enzymes, similar to the other cellulosome-producing bacteria. Among the 40 GH9s, 33 contain a dockerin. This is the most abundant GH family known today among the clostridia. In addition to dockerins, most of the cellulosomal GH9 enzymes contain CBM3 modules: fifteen CBM3c-possessing enzymes (two of them containing two CBM3s), fourteen CBM4-possessing enzymes. and two with CBM30s, in accordance with known modular architectures the CBM modules, would be expected to provide a significant contribution to enzyme action [46–48]. The wealth of the GH9 family in this bacterium indicates its important role in biomass degradation by the ability of its members to bind and hydrolyze cellulosic and xylan/xyloglucan substrates [34].

GH10 is the second most abundant family with 15 enzymes (Table 1), four of which are multifunctional enzymes together with an additional GH motif and a dockerin (Table 3). Four non-cellulosomal GH10 members are associated with CE4 and CBM22/CBM9 elements, and three of them contain a triple SLH repeat, suggesting that these enzymes are attached to the cell surface. Four of seven cellulosomal GH10 enzymes contain a CBM6, suggesting strong cellulose binding [34].

**Table 3:** Multifunctional cellulosomal proteins in *B. cellulosolvens*

Modular architecture	Accession Number
GH16-GH16-*Doc*	KNY27855.1
**GH43-** *Doc*-CBM42**-GH43**	KNY29222.1
**GH5**_8**-GH5_**8**-** *Doc*	KNY27224.1
**GH43-**CBM13-*Doc* **-GH16**	KNY26476.1
**GH11-**CBM6-*Doc* **-GH10**	KNY26370.1
**GH11-**CBM6-*Doc* **-GH10**	KNY27805.1
**GH11-GH10-** *Doc*-X124	KNY27822.1
**GH62-**CBM6-*Doc* **-GH10**	KNY27824.1
**GH11-GH10-** *Doc*-**CE4**	KNY28459.1
**GH8-** *Doc* **-CE4**	KNY25189.1
**GH8-** *Doc* **-CE3**	KNY25208.1
**GH8-** *Doc* **-CE4**	WP_081926996.1
**GH10-** *Doc* **-CE3**	KNY27825.1
**GH43-**CBM6-CBM6**-** *Doc* **-CE6**	KNY27842.1
**GH11-GH10-** *Doc* **-CE4**	KNY28459.1
**CE3-CE3-** *Doc*	WP_036941945.1
**PL1_5-**X60-*Doc* **-PL9_1**	KNY28878.1

Eleven enzymes were revealed as containing GH5 and GH43 modules, most of them cellulosomal (Table 1). In *C. thermocellum* and *A. cellulolyticus*, GH5 is the second most abundant family, but in *B. cellulosolvens* the representation is somewhat different. Similar to other bacteria, CBM3 is prevalent in the *B. cellulosolvens* genome with 30 representative proteins, 16 of which possess a dockerin module, mostly associated with GH and CE enzymes. *A. cellulolyticus* has 24 CBM3 members, 19 of which are associated with a GH9 [16]. In *B. cellulosolvens,* out of 30 CBM3s, 16 are associated with GH9 enzymes and 15 of them appear to be cellulosomal. CBM4 and CBM6 are less abundant, but represent a considerable part of the CBM family, with 23 and 20 members, respectively; 14 of these proteins in each group are associated with a dockerin. In general, we observed a very wide array of enzymatic and structural modules, which may collectively assemble into a robust machinery of both cellulosomal and free biomass-degrading components.

The variety of *B. cellulosolvens* GH catalytic modular representatives emphasizes the robustness of its cellulosome system. Another intriguing feature is the presence of 17 multifunctional enzymes (Table 3), which harbor a combination of at least two catalytic modules in the same polypeptide. Seven of these enzymes include two different GH families, and two have two catalytic modules from the same family—GH16 (GH16-GH16-*Doc*, KNY27855.1) and GH43 (GH43-*Doc*-CBM42-GH43, KNY29222.1), respectively (Table 3). There are also several mixed bifunctional hemicellulase/carbohydrate esterases, a dual carbohydrate esterase and a bifunctional polysaccharide lyase. Similar types of multifunctional protein architectures have been reported in *C. thermocellum* [46], *A. cellulolyticus* [16], *R. flavefaciens* [33], *Ruminococcus champanellensis* [19], and other bacteria [49], indicating that multifunctional enzymes are a common component in cellulosomal systems.

A high number of dockerins was associated with putative peptidases (71 proteins in total), suggesting a broader role of the cellulosomal complex in parallel with fiber degradation. Predicted peptidase modules were also found in scaffoldins ScaO and ScaP of *B. cellulosolvens* (associated with multiple PPC modules in addition to a single cohesin and dockerin), as well as in ScaO and ScaP of *A. cellulolyticus* [16]. The role of cellulosomal peptidases has not been defined experimentally, but recent studies have suggested the presence of peptidase modules associated with dockerins in the metagenome of the bovine rumen [50]. Similarly, in *R. flavefaciens* FD-1, numerous dockerins are associated with putative peptidase modules [33]. One putative cysteine peptidase associated with a C-terminal X-dockerin modular dyad from *R. flavefaciens* exhibited functional binding to the surface-anchoring ScaE cohesin [51].

### Characterization of the numerous scaffoldins and cohesins

Only two scaffoldins were previously reported in *B. cellulosolvens* [26, 28]. The present work revealed an unprecedented number (29) of *additional* cohesin-containing scaffoldins for a total of 31 *B. cellulosolvens* scaffoldins. Figure 2a presents the modular architecture of all the putative *B. cellulosolvens* scaffoldin proteins and their diverse types of cohesin and dockerin components. All scaffoldins (except ScaA2) contain a predicted signal peptide [52], suggesting that these proteins are secreted. Proteomics experiments indicated the presence of ScaA2 in the spent supernatant fluids of *B. cellulosolvens* growth cultures (data not shown), indicating that this scaffoldin was also secreted, despite the apparent lack of a signal peptide.

In naming the different *B. cellulosolvens* scaffoldins, we tried to compare their predicted architecture with those of previously described scaffoldins from other cellulosome-producing bacteria. Scaffoldins A to P (19 scaffoldins) contain cohesins and dockerins possessing modular arrangements similar to those of other known bacteria, particularly to those of *Acetivibrio cellulolyticus*, but with one important difference—the cohesin types are always reversed, i.e., if the primary cohesins of the homologous scaffoldins of *A. cellulolyticus* (and other species) are of type I, then those of *B. cellulosolvens* will be of type II and vice versa [16]. Remarkably, we observe this pattern in all *B. cellulosolvens* cellulosomal proteins that have orthologues in other cellulosome-producing bacteria.

**Fig. 3:**
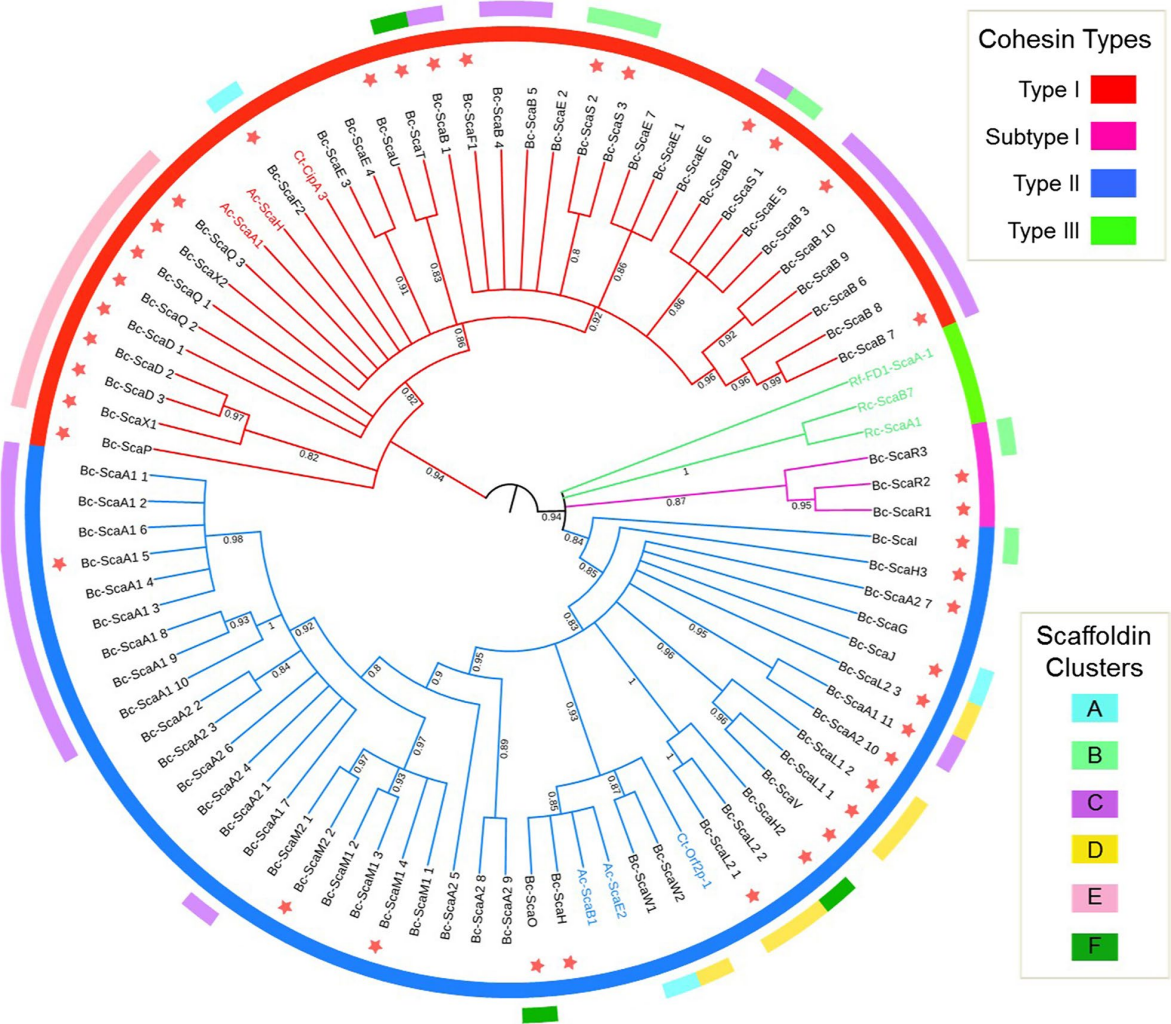
Phylogenetic tree of *B. cellulosolvens* cohesin modules. Cohesin modules were selected according to bioinformatic analysis, and the sequences were chosen individually to include only the cohesin sequence itself, without linkers or other scaffoldin modules. The final digit of each name represents the number of the specific cohesin from the N terminus in a multivalent scaffoldin. The phylogenetic tree was generated by iTOL according to the “One Click” Phylogeny analysis tool and aligned manually by BioEdit. *Colors* represent cohesin-type classification: *red lines* refer to type I cohesins; *blue line* to type II; *pink line* represent Group R cohesins, and *green lines* type III. Within the tree we used three sequences of known previously defined cohesins (from each type: I, II, and III) from other bacteria to serve as standards or markers for comparison (the names of the cohesins colored according to the type). *Red stars* represent cohesins that were expressed and experimentally tested in the present work for dockerin specificity. *Colors* in the *inner* (complete) *circle* facilitate visual identification of the cohesin types (see key, *above right*). *Colors* in the *outer* (fragmented) *circle* represent the cluster of the designated cohesins (see key, *below right*, and Fig. 2). Numbers on the branches represent bootstrap values

The cohesin modules within the scaffoldins exhibit a variety of intriguing sequence features. This bacterium also has some unique cohesin sequences which are somewhat different from the canonical type I or type II classification, according to the majority of known cellulosomal systems [21, 53]. Multiple sequence alignment of the cohesins can be found in Additional file 3: Figure S2 (the file includes scaffoldin accession numbers). Of the various *B. cellulosolvens* cohesins, 75 are classified into the two main types: type I (33 modules) and type II (42 modules). In addition to the canonical cohesin types I and II (and type III of the ruminococci), three *B. cellulosolvens* scaffoldins (ScaR1, ScaR2, and ScaR3) represented significantly different cohesin and dockerin sequences that exhibited only weak similarity to the main types and were therefore classified as ‘group R.’ We then examined the conservation patterns of the cohesin sequences, both within and among the different scaffoldins (Fig. 3). Clustered scaffoldins (Fig. 2b) may share homologous cohesins of similar types (ScaQ, ScaX1, ScaX2, and ScaD), although more distant cohesins may share some similarity as well. Two adjacent ORFs (scaffoldins ScaA1 and ScaB, Fig. 2) include different cohesin types, similar to the ScaA and ScaB pairs observed in *Clostridium thermocellum*, *A*. *cellulolyticus, and C. clariflavum* [16], albeit, as noted above, reversed in type.

Scaffoldins Q to X were found to share less similarity to the known scaffoldins from other bacteria and were named alphabetically taking into account intraspecies similarity (Fig. 2a). Overall, the modular organization of the proteins in *B. cellulosolvens* bear similarities to *A. cellulolyticus* and *C. thermocellum,* but the bacterium also contains new types of scaffoldins which were not described before (ScaQ–ScaX2). Most of the multiple cohesins within a scaffoldin are very similar, e.g., ScaA1 and ScaM1, but the phylogenetic tree also reveals variability among cohesin sequences, even within a single scaffoldin (Fig. 3). For instance, ScaA2 cohesins 6 and 10 are significantly distant from each other, although they are all classified as type II.

The numbers of the cohesins on the scaffoldins vary from a single cohesin (20 different scaffoldins) to 11 cohesins on ScaA1, the largest number of cohesin modules found on a single scaffoldin to date. ScaB, the adjacent downstream ORF of ScaA1, is an anchoring scaffoldin with 10 type I cohesins and an S-layer homology (SLH) domain, which is believed to form a non-covalent interaction with peptidoglycan-associated polymers to attach the protein to the cell surface [54]. These two largest ORFs are clustered on the genome, resembling the clusters described in other cellulosome-producing species, notably *C. thermocellum* [55], *A. cellulolyticus* [15, 18], and *R. flavefaciens* [12]. According to the bioinformatics analysis of regulatory regions flanking the *scaA* and *scaB* genes [8], it is not likely that *scaA1* and *scaB* of *B. cellulosolvens* are transcribed together. SLH domains and cell surface-binding modules (CSBMs) enable attachment of the anchoring scaffoldin to the bacterial cell surface [20, 56] and are present in nine scaffoldins (Fig. 2a). For some *B. cellulosolvens* scaffoldins (ScaB, ScaF2, and ScaJ), we observed the presence of an interesting SLH domain structure that includes a unique type of X-module at the N terminus, which differs from the X60 module of the ScaA subunit, known to stabilize dockerin interactions in *C. thermocellum, A. cellulolyticus, and C. clariflavum* [57]. The X-SLH modular dyad in scaffoldin proteins thus far seems to be unique to *B. cellulosolvens*. In addition to SLH and CSBM domains, we have identified other domains that may participate in anchoring of the scaffoldin to the cell wall or interactions with the substrate, including a PA14 domain of ScaU, a cadherin domain in ScaD (in addition to the SLH), VCBS in ScaH3, and a PPC (pre-peptidase C-terminal) domain in ScaO and ScaP (Fig. 2a).

**Fig. 4:**
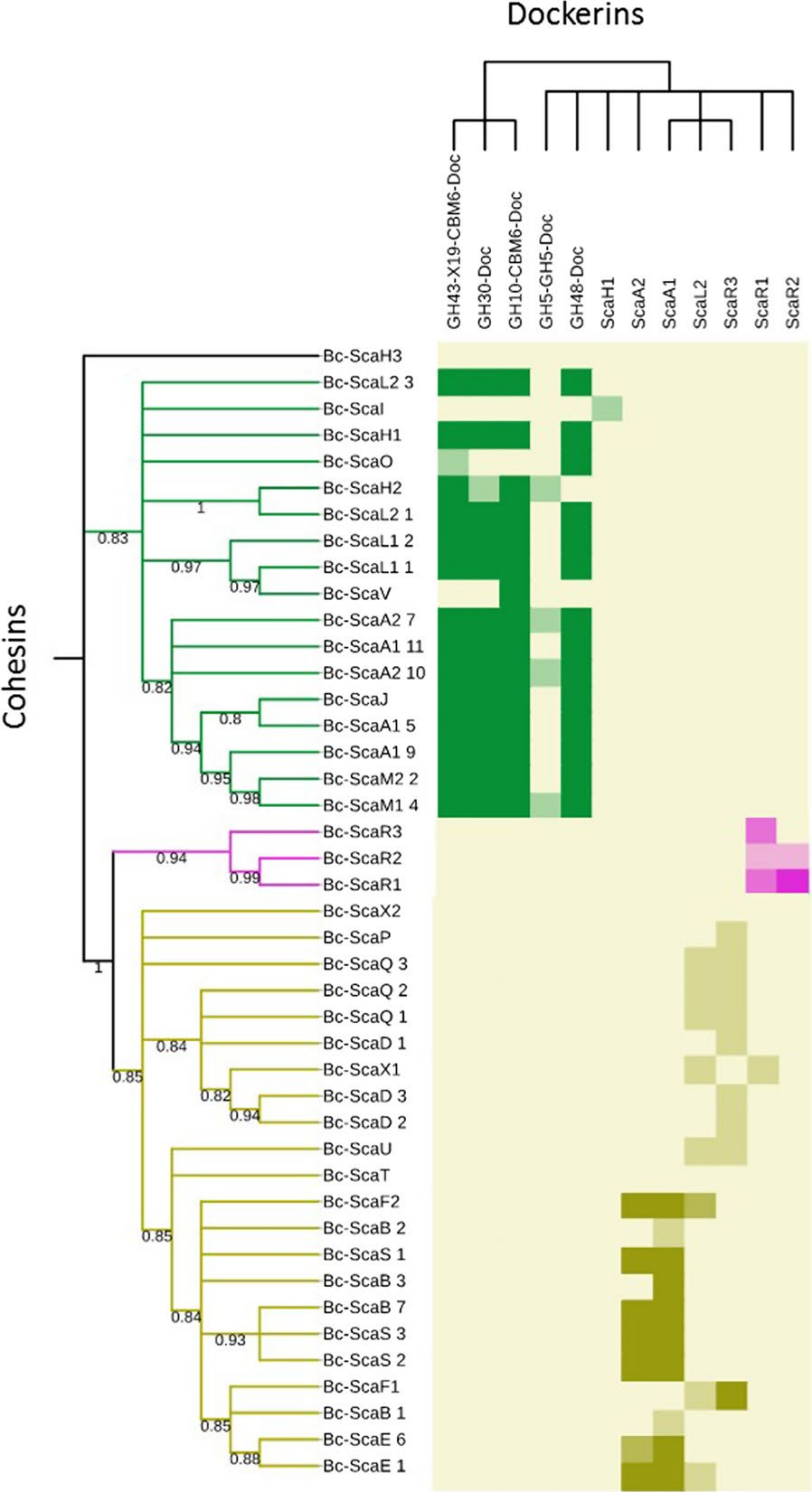
Determination of cohesin-dockerin specificity by affinity-based ELISA. The 96-well ELISA plates were coated with the desired CBM-Coh fusion proteins and variable concentrations of Xyn–Docs were applied to detect specific cohesin–dockerin interactions. *Doc* dockerin, *ScaA1 5* scaffoldin name followed by the number (position) of the cohesin. Type II cohesin interactions are shown in *green*, Type I in *light khaki* and group-R scaffoldins in *pink*. The strength of interaction (*color intensity squares*) was determined according to the OD results as defined in the “Methods” section (the *stronger colors* represent strong interaction). The cohesins (*left column*) and dockerins (*upper row*) appear in the table according to phylogenetic relationships with bootstrap values represented for cohesins

The *B. cellulosolvens* genome contains genes for seven scaffoldins with no dockerin, CSBM, or SLH domain, which implies that they may serve as cell-free scaffoldins, which contain either type I or type II cohesins (Fig. 2a). Two examples of this type of scaffoldin, ScaE and ScaS, bear type I cohesins, potentially forming cell-free cellulosomes with up to 77 dockerin-bearing enzymes (Fig. 4). Intriguingly, *C. thermocellum, A. cellulolyticus, and C. clariflavum* all produce ScaE homologues, bearing seven type II cohesins in these species (for binding primary scaffoldins, reverse in type, compared to *B. cellulosolvens*). In *B. cellulosolvens,* enzyme-binding type II cohesins from the cell-free primary scaffoldins ScaM1 and ScaM2 have a CBM3, which would bind the substrate and thus allow targeted degradation by the enzymes. Enzyme-integrating CBM-bearing ScaM homologues (with CBM2s) have been detected in the cellulosome systems of *A. cellulolyticus and C. clariflavum* but not *C. thermocellum.*

### Classification of the dockerins into types based on sequence homology

In *B. cellulosolvens* all of the type I dockerins are associated with primary scaffoldins (Fig. 2a; Additional file 4: Figure S3). Scaffoldins ScaR1, ScaR2, and ScaR3 possess a unique type of dockerin that did not fit the main types and were collectively termed ‘group R.’ Similarly, the ScaP dockerin with its unique sequence was not included into any of the main dockerin types (Additional file 4: Figure S3).

**Fig. 5:**
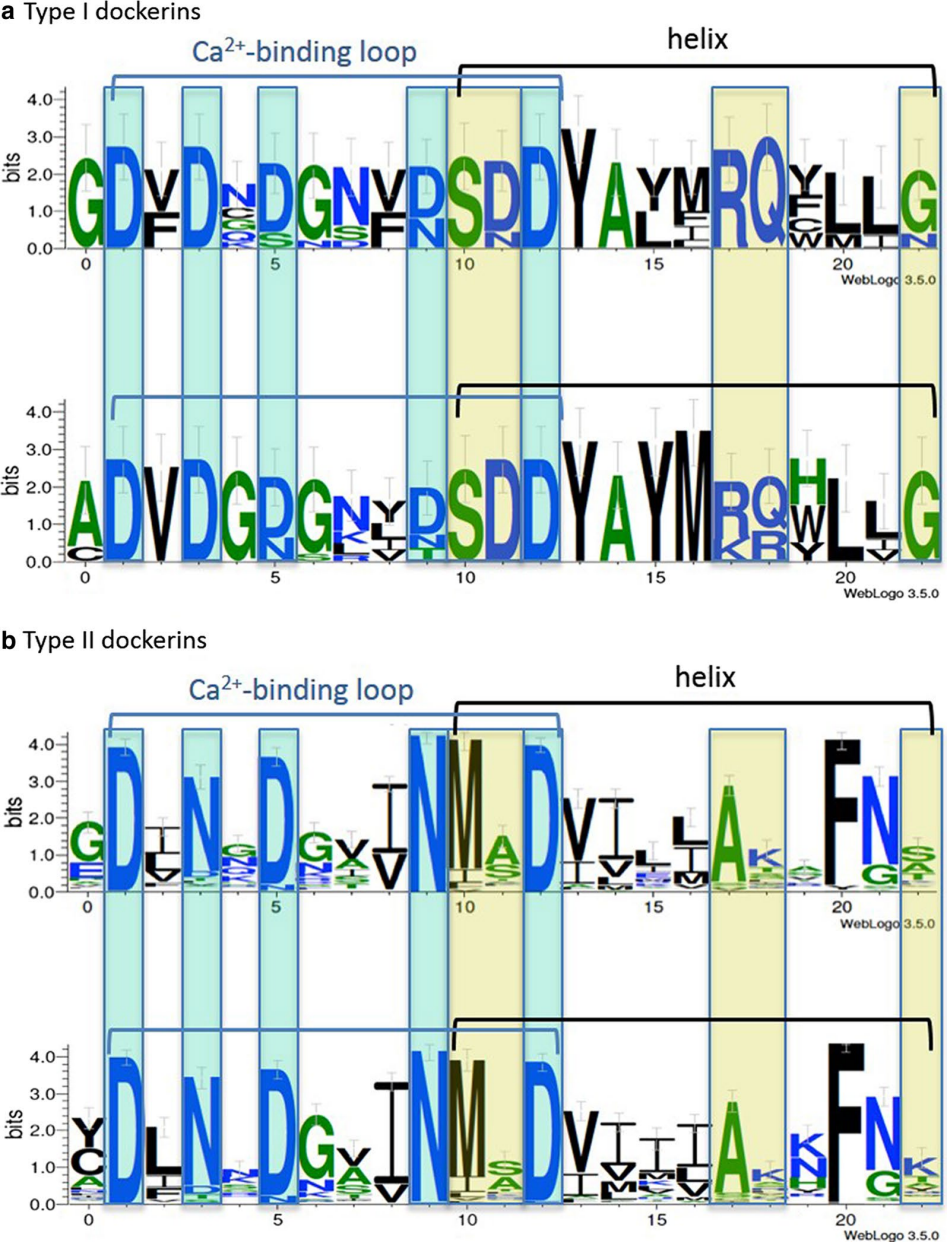
Sequence conservation pattern of type I and type II dockerin modules. **a** The two internal type I dockerin repeats of *B. cellulosolvens* (based on five type I dockerin sequences) are represented by sequence logos. **b** The two internal type II dockerin repeats of *B. cellulosolvens* (based on 146 type II dockerin sequences) are represented by sequence logos. The upper logo represents the first repeat of the duplicated sequence, and the lower logo represents the second. Positions of calcium-coordinating residues (usually D or N) are shown in *light cyan*, and putative recognition residues are shown in *light yellow*

Dockerin modules are characterized by two duplicated segments, consisting of a calcium-binding loop that precedes an α helix (Fig. 5), connected by a linker sequence, with distinctive N- and C-terminal stretches [58]. Dockerins usually display a conserved pattern within the given type. Type I dockerins would presumably bind type I cohesins within the same bacterial species, and the same applies to types II [59].

*Bacteroides cellulosolvens* dockerins were herein classified into two major previously defined groups (type I and type II) and a new group (group R that includes ScaR1-3 dockerins). Almost half of the dockerins are located downstream of an X-module and have distinctive sequence features compared to the rest of the *B. cellulosolvens* dockerins. Their X-modules belong to family X60, which displays significant sequence identity (30–57%) with the X-module at the C-terminus of the *C. thermocellum* CipA scaffoldin [57]. As mentioned above, these X-modules are known to stabilize their adjacent dockerin and render them more soluble [60]. In addition, X60-modules were described at the C-terminus of the primary scaffoldin of *A. cellulolyticus* and *C. clariflavum*, all related to type II dockerins [16, 17]. The high number of X60 modules in *B. cellulosolvens* may indicate their crucial role in the dockerin interactions. Intriguingly, all of the type II dockerins described previously were accompanied by a neighboring X-module [16, 17, 57], but here, in *B. cellulosolvens,* the presence of many proteins containing type II dockerins (111) lacking the adjacent X-module remains an enigma. The sequence alignment of the type II dockerins is presented in Additional file 5: Figure S4 and Additional file 6: Figure S5.

Prior to the sequencing of the *B. cellulosolvens* genome, the scientific community was cognizant of only a few type II dockerin and cohesin sequences. Only the type II dockerins of the primary scaffoldins of C*. thermocellum* [11, 61], *A. cellulolyticus* [16], *and C. clariflavum* [17], and the early discovery of the *B. cellulosolvens* Cel48 dockerin [29] had been reported. The 197 type II dockerins revealed by this genome has thus significantly enriched our understanding of this very basic dockerin type.

The characteristic sequence conservation profile of the *B. cellulosolvens* type II dockerin is shown in Fig. 5 [21, 53, 59]. Examination of the putative recognition residues revealed the highly conserved calcium-coordinating residues: Asp in positions 1 and 12; mostly Asn/Asp in position 3; Asn/Asp in position 5; and Asn in position 9. Positions 3, 5, and 9 are infrequently replaced by Ser, Thr, and sometimes Lys. Other variations have also been observed [24, 25, 31]. The putative calcium-binding residues are consistent with those of type II dockerins known from the literature [57], and the predicted recognition residues show Met and Ala dominating in positions 10 and 17, respectively. Interestingly, Phe dominates at position 20 of the helix and Asn and Gly are prevalent at position 21, consistent with the few previously known type II dockerin sequences.

### Selection of cohesins and dockerins

To shed light on cellulosome assembly in this unique bacterium, multiple cohesins and dockerins were selected for further experimental investigation. A total of 43 cohesins and 27 dockerins were cloned as fusion proteins with a solubility/stability tag, expressed and purified. The solubility tags, CBM3 from *C. thermocellum* CipA and xylanase T6 from *G. stearothermophilus* for the cohesin and dockerin modules, respectively, also served as general affinity tags for semiquantitative detection of the cohesin–dockerin interaction by specific antibodies directed against them using an ELISA-based system [35].

The initial alignment of the cohesin (Additional file 3: Figure S2) and dockerin sequences (Additional files 4, 5, 6: Figures S3–S5) served to determine their type distribution and was used for the selection of cohesins and dockerins for biochemical characterization. Despite the very large number of modules in this bacterium, we tested experimentally at least one cohesin from nearly all of the scaffoldins as well as the most divergent cohesins within a given multiple-cohesin scaffoldin (Fig. 2a). Only three scaffoldins: ScaW1, ScaW2, and ScaG that were discovered by scrutinizing the genome at a later stage of the study were not tested. Almost all of the scaffoldin-borne dockerins (except ScaO and ScaH2) were selected, owing to their important role in cellulosome assembly. In addition, we selected 17 dockerins that diverged in their sequences from the most prevalent GH modules (e.g. GH5, GH8, GH9, GH10, GH30, GH43, and GH48). When an X-module was adjacent to the selected dockerin, it was included in the cloned sequence.

Following expression and purification of these 70 proteins, SDS-PAGE analysis revealed single major protein bands in each case, in agreement with their calculated molecular mass.

### Identification of cohesin–dockerin interactions

**Fig. 6:**
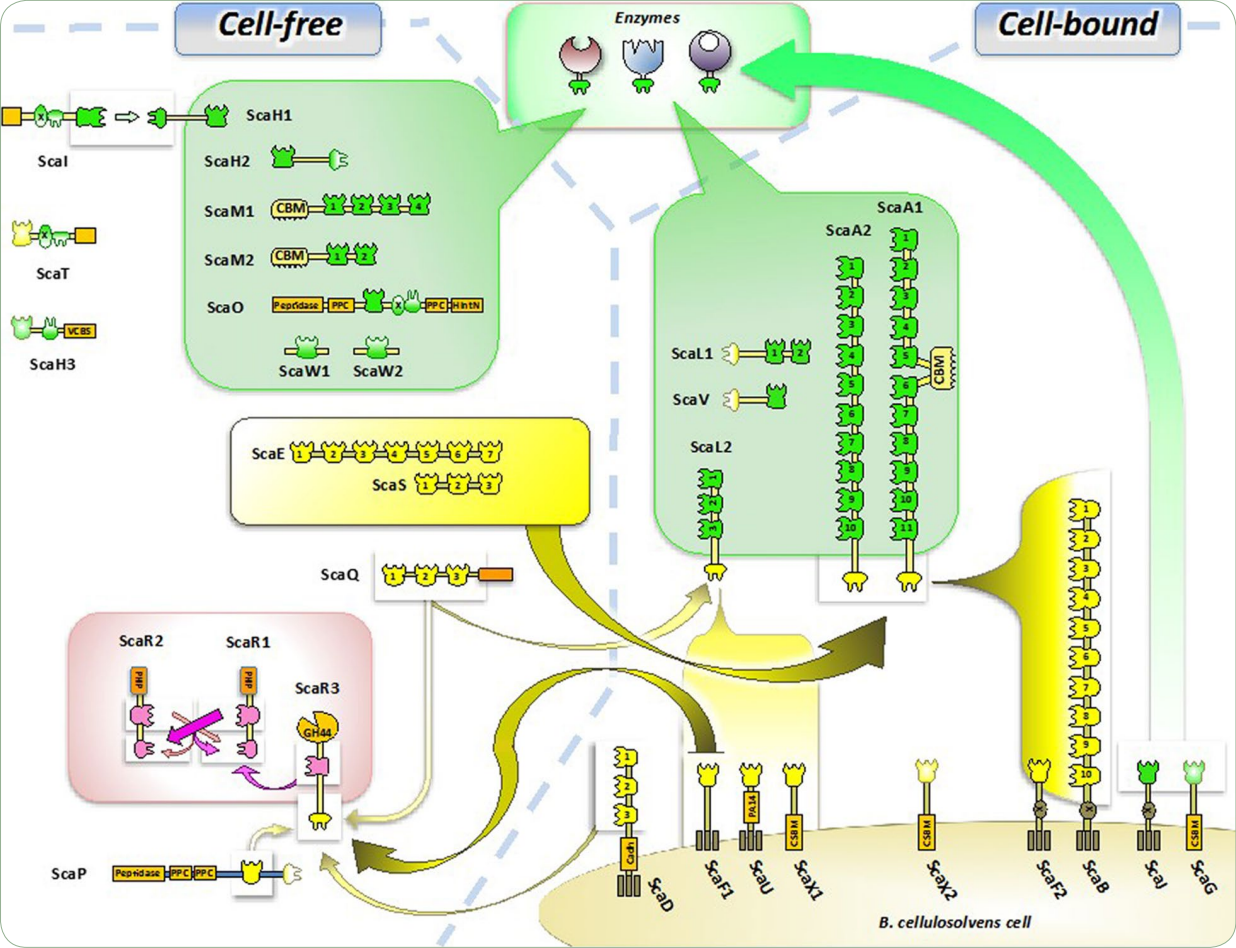
Intricacy of the *B. cellulosolvens* cellulosome assemblies. The scheme shows possible interactions among scaffoldins and enzymatic modules, as derived from examination of interactions by affinity ELISA, where binding specificities of the cohesin-borne scaffoldins are detailed in Fig. 4. The type II enzyme-borne dockerins generally bound very strongly to the cohesins of ScaA1/A2, ScaJ, ScaH1/H2, ScaL1/L2 and ScaM1/M2, ScaO and ScaV. The type I dockerins of ScaA1 and its ScaA2 sibling interacted strongly with the multiplicity of ScaB cohesins and the singular cohesin of ScaF2, which would anchor them and their associated enzymes to the *B. cellulosolvens* cell surface. Single enzymes can also be anchored directly to the cell wall via type II interaction with the ScaJ cohesin. Large *secreted* cell-free assemblies would ensue from strong type I interactions between the ScaA1/A2 dockerins with the cohesins of ScaE and ScaS. Smaller cell-free complexes would comprise the direct type II interaction between enzymes and ScaM1 and ScaM2, both of which contain a CBM3 for targeting to the cellulosic substrate. Finally, the strong type I interaction between the ScaR3 dockerin and the single F1 cohesin would serve to connect the group-R scaffoldins to the cell surface. All other cohesin–dockerin interactions detected within the framework of this study appeared to be much weaker, and the resultant complexes would presumably be less stable. ScaW1, ScaW2, and ScaG were not tested empirically, owing to their late discovery

In total, 103 positive interactions were detected (Fig. 4). The specificity of the various cohesin and dockerin counterparts revealed in this study served to determine the theoretical supramolecular organization of its known cellulosomal components. The cellulosomal architectures of *B. cellulosolvens* cellulosome are represented in Fig. 6. Our analyses underscore the highly heterogeneous and diverse supramolecular architecture of this cellulosome system.

In accordance with previous reports [28, 29], our results indicated that the type I ScaB cohesins bind selectively to the ScaA1 dockerin, whereas the GH48 (WP_050753099.1) dockerin binds specifically to the type II ScaA1 cohesins (Fig. 4). The cohesins of ScaA1 also bound to various cellulosomal enzymes, in particular to GH10 (WP_036936763.1), GH43 (WP_081926929.1), and GH30 (WP_081927211.1). In addition, cohesins of other primary scaffoldins (namely, ScaA2, ScaH1, ScaH2, ScaJ, ScaL2 ScaM2, and ScaO) shared the same binding specificities as ScaA1 (Fig. 4). Type II cohesins from scaffoldins ScaL1 and ScaV showed clear preference to bind type II dockerins but with lower intensity. The type II dockerin of ScaI failed to show any interaction, but its type II cohesin showed low levels of interaction with the ScaH1 dockerin.

Some of the tested enzyme-borne dockerins, i.e., two dockerin modules from GH9 enzymes (WP_050753192.1; KNY25939.1) and a GH5 with an X60-dockerin modular dyad (WP_050753119.1), failed to show binding specificity to ScaA1 cohesins or those of other primary scaffoldins. In addition, six of the selected dockerins failed to interact with any of the selected cohesins: one originated from an ORF containing only two dockerin modules (WP_036940956.1), another from an ORF containing a putative peptidase and two similar dockerins (WP_036945116.1), one GH30-associated dockerin along with its X60 module (KNY28903.1), dockerin modules from scaffoldins ScaI and ScaT along with their adjacent X60 and an X60-dockerin pair originating from a GH43 module (KNY26505.1). The dockerin sequence of the latter is similar to another dockerin from a GH43 enzyme (WP_038290784.1) that exhibited high binding interaction (Fig. 4). In this particular case, we designed two Xyn–Doc fusions with or without the X-module and expressed the full-length enzyme but none of those constructs exhibited any binding activity.

## Discussion

The recent sequencing of the *B. cellulosolvens* genome enabled comprehensive bioinformatics identification of the numerous cellulosomal components and cell-anchoring modules. The high quality of the genome sequence [30] allowed us to identify an unprecedented number of scaffoldins in this bacterium. The cohesin and dockerin modules contain some unique and intriguing sequences, which were separated on the basis of bioinformatics and complementary biochemical analysis into the two major conventional types and one novel group—group R, which contain only a few members.

The cellulosomal system of *B. cellulosolvens* represents the most complex so far discovered in nature, by virtue of its 31 different scaffoldins—nearly four times that of the *C. thermocellum* standard (i.e., initially discovered cellulosome system) and double that of *A. cellulolyticus*, the next most extensive known system. Theoretically, *B. cellulosolvens* can assemble up to 110 enzymes in a single, cell-associated complex (Fig. 6), consisting of only two interacting scaffoldins, ScaA1 and ScaB, without the aid of an adaptor scaffoldin [18]. In addition, its genome contains numerous free scaffoldins (which lack any apparent cell surface-binding modules) that interacted specifically with selected dockerins. This putative cell-free cellulosome system may serve an important role of degrading carbohydrates distant from the bacterial cell, as hypothesized earlier [62]. Free cellulosomes have been described before for several cellulosome-producing bacteria and are believed to contribute to a more efficient carbohydrate degradation [17, 19]. In this context, ScaE (which contains seven type I cohesins) and ScaS (which contains three type I cohesins) were shown to bind primary scaffoldins (Fig. 4) and could assemble into comparatively large free cellulosome complexes (Fig. 6). The additional cell-free scaffoldins, ScaM1 and ScaM2, contain type II cohesins along with a CBM3 module that would target the attached enzymes to the substrate. In contrast, monovalent (single cohesin) free scaffoldins (ScaW1, ScaW2) may either serve as molecular shuttles, stabilize enzyme activity through cohesin–dockerin interaction, or serve a regulatory function [63–65]. Multiple monovalent scaffoldins are widespread among complex cellulosome-producing bacteria [16, 19, 66] and could be part of a regulatory mechanism for cellulosomal composition.

Previous research suggested that a dual-binding mode of the dockerins would result in increased flexibility characteristics of the catalytic subunits [67, 68], it was reported previously that the type I ScaA dockerin has a dual-binding mode in the recognition of the *B. cellulosolvens* type I cohesins [69]. The additional type I dockerins reported here share similar binding preferences and sequence similarity. It can therefore be assumed they also have a dual mode of binding. In this context, the meaning of “reversed” cohesin types in *B. cellulosolvens* would indicate higher requirement in flexibility of scaffoldin assembly (type I interactions) rather than in enzyme integration (type II interactions).

As has been reported for other cellulosome-producing bacteria, some of the *B. cellulosolvens* scaffoldin genes are assembled in *sca* gene clusters (Fig. 2b) [16, 70]. Here, most of the clusters (with the exception of Cluster B) are only composed of scaffoldin genes without any GH genes. Cluster C consisted of genes coding for the two major multiple cohesin-carrying proteins (ScaA1 and ScaB) and represents the classic *sca* cluster [71]. However, we also find additional scaffoldin clusters in the *B. cellulosolvens* genome: cluster E, formed from genes for ScaD, ScaX1, ScaX2, and ScaQ, also includes genes encoding two ABC (ATP-binding cassette) transporters: one in between *scaX2* and *scaX* and another (including a C-terminal dockerin) downstream of *scaX1*. The ABC transporter may assist in uptake of degraded carbohydrates by the cell and the dockerin-bearing ABC transporter seems to result from a fusion of two ORFs. ScaD, ScaX1, and ScaX2 all include cell-anchoring elements (an SLH and CSBMs, respectively). Interestingly, ScaQ has an unknown protein module, which might serve a similar cell-anchoring function, considering its location on the same cluster. Moreover, the type I cohesins of the four genes are all very similar in sequence composition (Fig. 3). Only one cluster that contains genes encoding ScaI, ScaS, and ScaR3 (Fig. 2b), includes downstream genes for a GH9 and a two-component sensor, which may participate in their regulation.

The binding specificities of some of the expressed cohesins and dockerins remain unknown (data not shown). Possible reasons could be inappropriate folding of the recombinant fusion proteins or the fact that a relevant cohesin partner was not among those selected in our study. Intriguingly, none of the selected X60-linked dockerins (both scaffoldin- and enzyme-associated) bound to any of the cohesins. Since the X60 module is widely represented in the *B. cellulosolvens* genome, we expected to observe positive interactions for X60-linked dockerins. Indeed, a previous study reported that X60-dockerin modular dyads from other bacteria did in fact exhibit binding interactions with appropriate type II cohesins [26]. For some cohesins and dockerins, the expression of longer scaffoldin sequences that included linkers or additional cohesin(s) improved significantly the binding capacities. This suggests, that not only the specific cohesin sequence is important for dockerin binding, but the adjacent protein sequence and modular structure could impact the stabilization of the interaction. Previous research also emphasized the importance of linker length and specific position of a module in a given scaffoldin [72].

Interestingly, the ScaL1 dockerin, that failed to show any binding activity for the selected cohesins, exhibited high-affinity binding with the lysate of *B. cellulosolvens* grown on cellobiose (Additional file 7: Figure S6). Despite the fact that we failed to demonstrate an in vitro interaction (for the reasons stated above) between the ScaL1 dockerin and any of the cohesins tested, this result confirms that the module presumably interacts with its partner in vivo.

In addition to the intraspecies interactions described in this work, we revealed inter-species interaction between the type II *B. cellulosolvens* cellulosomal components and those derived from two other cellulosomal bacteria: *A. cellulolyticus* and *C. clariflavum* (Additional file 8: Figure S7). These results raise the possibility of inter-species cross-reactivity, which may reflect diversification and increased cellulosomal degradation capacities for efficient carbohydrate degradation in nature. It was particularly interesting to examine the interaction between the same types of cohesins and dockerins, which are reversed in all other known bacteria in comparison to *B. cellulosolvens*. Thus, in the case of inter-species interaction, the cohesins from primary scaffoldin ScaA1 of *B. cellulosolvens* (type II) successfully bound a dockerin harbored by an adaptor scaffoldin (type II in other species). It is interesting to note that both *B. cellulosolvens* and *A. cellulolyticus* were originally isolated from sewage sludge, and *C. clariflavum* was first isolated from an anaerobic thermophilic methanogenic sludge. In this context, cross-species interaction among the different type II components has indeed been observed previously [26], and the ability of the *B. cellulosolvens* primary scaffoldin ScaA1 to bind strongly to the primary ScaA scaffoldins of either the thermophilic *C. clariflavum* or the mesophilic *A. cellulolyticus* via type II cohesin–dockerin interactions may indicate its complex relationship with other cellulose-degrading microbes within a specific ecological niche.

## Conclusions

The present work has revealed the binding properties of a large number of cellulosomal elements and described a multiplicity of potential cell-free or cell-associated elaborate cellulosomal arrangements in *B. cellulosolvens.* These cell-free or cell-associated cellulosome complexes could be targeted to the polysaccharides substrate and include an extremely large variety of different plant cell wall-degrading enzymes and proteases via multiple scaffoldin assemblies. The accumulated knowledge of the cellulosomal components in newly discovered cellulosome-producing bacteria enables comparative evaluation of the variety of possible cellulosome architectures and/or cohesin-dockerin functions in as-yet-undescribed and/or uncharacterized cellulosome-producing bacteria. Moreover, the extensive cellulosomal system of *B. cellulosolvens* bears potential to provide a significant reservoir of novel components for subsequent cellulosomal research thus promoting future application of designer cellulosomes and other types of biotechnological assemblies [72, 73].

## Additional files

**Additional file 1: Table S1.** List of primers for the *Bacteroides cellulosolvens* cohesin and dockerin modules that were cloned in this study. Restriction enzyme sites are shown in bold.

**Additional file 2: Figure S1.** Hydrolysis of beechwood xylan by cellulosome fractions of *Bacteroides cellulosolvens*. The two cellulosomal complexes (high-molecular-weight complex and low-molecular-weight complex) isolated from two different growth media (CB and MCC) were tested for their catalytic activity on beechwood xylan in order to demonstrate its ability to degrade it. The *Clostridium* *thermocellum* cellulosome (kindly provided by CelDezyner LTD, Rehovot, Israel) was also tested for catalytic activity as a positive control of the catalytic activity of the *B. cellulosolvens* cellulosomes. CB, cellobiose; MCC, microcrystalline cellulose; Ct, *Clostridium* *thermocellum*.

**Additional file 3: Figure S2.** Multiple sequence alignment of 87 cohesin sequences, originating from the genomes of *Bacteroides cellulosolvens*, *Acetivibrio cellulolyticus* (Ac), *Clostridium thermocellum* (Ct), *Ruminococcus flavefaciens* (Rf) *and Ruminococcus champanellensis* (Rc). Alignment length: 175; Strongly similar (:): 1 residue =0.57%; Weakly similar (.): 0 residue =0.57%.

**Additional file 4: Figure S3.** Multiple sequence alignment of the five *Bacteroides cellulosolvens* type I and miscellaneous dockerin modules. The alignment shows two internal dockerin repeats of *B. cellulosolvens* type I and miscellaneous dockerins that contain unique sequences. The left part of the sequence represents duplicated sequence 1, and the right sequence part represents duplicated sequence 2. Cyan highlight indicates putative calcium-binding residues. Yellow highlight indicates putative recognition residues. **A**: Alignment length: 71. Identity (*): 30 residues = 42.3 %. Strongly similar (:): 11 residues = 15.5 %. Weakly similar (.): 9 residues = 12.7 %. **C**: Alignment length: 65. Identity (*): 28 residues = 43.1 %. Strongly similar (:): 17 residues = 26.2 %. Weakly similar (.): 7 residues = 10.8 %. **D**: Alignment length: 78. Identity (*): 7 identical residue = 9 %. Strongly similar (:): 13 residues = 16.7 %. Weakly similar (.): 5 residues = 6.4 %. **E**: Fragmented dockerins: Two ORFs that resemble a dockerin sequence were found in the genome. One of the two ORFs was not annotated and presented with its ordinal number – ORF1413.

**Additional file 5: Figure S4.** Multiple sequence alignment of the 146* Bacteroides cellulosolvens* type II dockerin modules. The alignment shows two internal dockerin repeats of* B. cellulosolvens* and was used to create Figure 6 representing the Weblogo of the dockerin repeats. The left part of the sequence (before the hyphen) represents duplicated sequence 1 and the right part (after the hyphen) represents duplicated sequence 2. Cyan highlight indicates putative calcium-binding residues. Yellow highlight indicates putative recognition residues. Alignment length: 65. Identity (*): 3 residues = 4.6 %. Strongly similar (:): 3 residues = 4.6 %. Weakly similar (.): 4 residues = 6.2 %.

**Additional file 6: Figure S5.** Multiple sequence alignment of the 49 miscellaneous* Bacteroides cellulosolvens* type II dockerin modules. The alignment shows two internal dockerin repeats of the* B. cellulosolvens* type II dockerins that contain unique sequences (particularly in the first calcium-binding loop) but remain type II dockerins. The left part of the sequence (before the internal hyphens) represents duplicated sequence 1 and the right part (after the internal hyphens) represents duplicated sequence 2. Cyan highlight indicates putative calcium-binding residues. Yellow highlight indicates putative recognition residues. Alignment length: 77. Identity (*): 5 residues = 6.5%. Strongly similar (:): 9 residues = 11.7%. Weakly similar (.): 3 residues = 3.9%.

**Additional file 7: Figure S6.** Determination of dockerin specificity to *Bacteroides cellulosolvens* cell lysate components by affinity-based ELISA. The 96-well ELISA plates were coated with *B. cellulosolvens* cell lysate (grown on cellobiose), and various concentrations of Xyn-Docs were used to detect cohesin-dockerin interactions. Abbreviations: Doc, dockerin.

**Additional file 8: Figure S7.** Determination of the inter-species interactions of *Bacteroides cellulosolvens* cell lysate by affinity-based ELISA. The 96-well ELISA plates were coated with *B. cellulosolvens* cell lysate (grown on cellobiose) and various concentrations of Xyn-Docs from three different bacteria were used to detect cohesin-dockerin interactions. Abbreviations: Doc, dockerin; Cc, *Clostridium clariflavum*; Rf, *Ruminococcus flavefaciens*; Ac, *Acetivibrio cellulolyticus*; GH9, Glycoside hydrolases of family 9. The dockerins were chosen to include the three previously defined types: Ac-GH9-Doc is a representative of type I dockerins; Cc-ScaA-Doc and Ac-ScaA-Doc represent type II dockerins; and Rf-ScaA-Doc represents type III dockerins. Here we show that *B. cellulosolvens* cell lysate is capable of crossreaction with type II dockerins from the primary scaffoldin ScaA from two different bacteria but not type I or III.

## Abbreviations

Cadh: cadherin
CBM: carbohydrate-binding module
CE: carbohydrate esterase
Coh: cohesin
CSBM: cell surface-binding module
Doc: dockerin
FN3: fibronectin type III domain
ELISA: enzyme-linked immunosorbent assay
GH: glycoside hydrolase
HintN: splicing and auto-cleavage bacterial intein-like domain
LRR: leucine-rich repeat
ORF: open reading frame
PA14: anthrax protective antigen domain
PL: polysaccharide lyase
PHP: polymerase and histidinol phosphatase domain
PPC: bacterial pre-peptidase C-terminal domain
Sca: scaffoldin
SLH: S-layer homology
VCBS: repeat domain in *Vibrio, Colwellia, Bradyrhizobium, and Shewanella*
XDoc: X-module coupled with a type II dockerin
Xyn: xylanase
