# Supplementary material for: Unique organization and unprecedented diversity of the Bacteroides (Pseudobacteroides) cellulosolvens cellulosome system
Source: Biotechnol Biofuels. 2017 Sep 7;10:211. doi: 10.1186/s13068-017-0898-6 (PMC5590126; doi:10.1186/s13068-017-0898-6)
Supplement: Supplementary file 1 — Additional file 1: Table S1. List of primers for the Bacteroides cellulosolvens cohesin and dockerin modules that were cloned in this study. Restriction enzyme sites are shown in bold. [file 13068_2017_898_MOESM1_ESM.docx]

**Additional File 1:**

**Table S1**: **List of primers for the *Bacteroides cellulosolvens* cohesin and dockerin modules that were cloned in this study.** Restriction enzyme sites are shown in bold.

| **Primer name** | **Nucleotide sequence** |
| --- | --- |
| ScaA.c5_F.BamHI | ATCATC**GGATCC**GGTTCAGGAGTAGTAGCAAC |
| ScaA.c5_R.XhoI | AGTATC**CTCGAG**TTATCCGTTTATTGAAGAAGCCTG |
| ScaA_c9_F_BamHI | ATCATC**GGATCC**GGTGAAGTGTTTGTAACATTTG |
| ScaA_c9_R_XhoI | ATCATC**CTCGAG**TTAACCATTAACTGCTGGTGCCTG |
| ScaA_c11_F_BamHI | GTCGTC**GGATCC**GGATCTGTATTGACAGCTATTG |
| ScaA_c11_R_XhoI | GTCGTC**CTCGAG**TTATGTGCCTTTTGGATAGATGAG |
| ScaB_c1_F_BamHI | ATCATC**GGATCC**GCAACGCCTCAAGTTAATATC |
| ScaB_c1_R_XhoI | ATCATC**CTCGAG**TTAACTTGTGCTAACATCTGAAAC |
| ScaB_c2_R_XhoI | ATCATC**CTCGAG**TTAACTGCTTTCTATATCTTTTAC |
| ScaB_c3_F_BamHI | ATCATC**GGATCC**GGGAAAAGTTCACCAGGAAAT |
| ScaB.c3_R_XhoI | GTCGTC**CTCGAG**TTAAGTTACAGTAATGCTTCCATC |
| ScaB_c7_F_BamHI | ATCATC**GGATCC**ATGTACTGGATGAATGTAGTG |
| ScaB_c7_R_XhoI | ATCATC**CTCGAG**TTATGGTGCTGTAGCTGCAACTG |
| ScaA2_c7_F_BamHI | ATCATC**GGATCC**AGCAAAGGTGAAGTATACATG |
| ScaA2_c7_R_XhoI | ATCATC**CTCGAG**TTATCCAATATTTATTGATTGTGG |
| ScaA2_c10_F_BamHI | ATCATC**GGATCC**GACGGATCTGTAAAGATGAC |
| ScaA2_c10_R_XhoI | ATCATC**CTCGAG**TTAATAAATACTTGCAGGAGATATA |
| ScaD_c1+c2_F_BamHI | ATCATC**GGATCC**GTAATTTTGTCTTTGGCAAAG |
| ScaD_c1+c2_R_XhoI | ATCATC**CTCGAG**TTACACTTCATAATCTGAGTAAAAG |
| ScaD_c3_F_BamHI | ATCATC**GGATCC**AATGCCATGAAGATATCTTTAAG |
| ScaD_c3_R_XhoI | ATCATC**CTCGAG**TTAATTTGAAACTGTTATCTTCCC |
| ScaE_c1_F_BamHI | ATCATC**GGATCC**GATTCATCGGTTAATGTAATC |
| ScaE_c1_R_XhoI | ATCATC**CTCGAG**TTATGCTTTCACATTACCGTTCAC |
| ScaE_c6_F_BamHI | ATCATC**GGATCC**CAGGAGCATAAGCTGATTGTAG |
| ScaE_c6_R_XhoI | ATCATC**CTCGAG**TTAAGCAACCGCTTCAACAGTAATT |
| ScaF1_c1_F_BamHI | ATCATC**GGATCC**TCATTAGTTGCCTTTTCAGC |
| ScaF1_c1_R_XhoI | ATCATC**CTCGAG**TTAATTTTGACCTGGTTTAGATGG |
| ScaF2_c1_F_BamHI | ATCATC**GGATCC**GTAAGTATAAGCATAGGTTCAG |
| ScaF2_c1_R_XhoI | ATCATC**CTCGAG**TTAATTTGTAACCGTTATGGCACC |
| ScaH1_c1_F_BamHI | ATCATC**GGATCC**ATCATGCCTACAGGAAAAATC |
| ScaH1_c1_R_XhoI | ATCATC**CTCGAG**GTTAATTCTCTGTGGCTGTA |
| ScaH2_c1_F_BamHI | ATCATC**GGATCC**GCTGATACAGCTGAAAAAGG |
| ScaH2_c1_R_XhoI | ATCATC**CTCGAG**TTATATTTTTCCAGGTTGAATTAT |
| ScaH3_c_F_BamHI | ATCATC**GGATCC**GCAATTAATCCGTCAATCTC |
| ScaH3_c_R_XhoI | ATCATC**CTCGAG**TTATGTTGATGAATTTTCAAAAG |
| ScaI_c1_F_BamHI | ATCATC**GGATCC**ATTTATCTTTCTTATAATAGG |
| ScaIc1_R_XhoI | ATCATC**CTCGAG**TTAAGGTGTTATGGATGTTACC |
| ScaJ_c1_F_BamHI | ATCATC**GGATCC**TTTATACTTGAGGCGGACC |
| ScaJ_c1_R_XhoI | ATCATC**CTCGAG**TTATCCGATCAATTCTACAGTTCC |
| ScaL1_c1_F_BamHI | ATCATC**GGATCC**ATTTCTTCTGGACCTGAATT |
| ScaL1_c1_R_XhoI | ATCATC**CTCGAG**TGTATTTGAAGGCACTGATG |
| ScaL1_c2_F_BamHI | ATCATC**GGATCC**CTTCTAAAAGTAACATTAAACG |
| ScaL1_c2_R_XhoI | ATCATC**CTCGAG**TTATATTGTCATAGAAGAAGGTTC |
| ScaL2_c1_F_BamHI | ATCATC**GGATCC**GCTGATATAAAGGGTAGTCTTAC |
| ScaL2_c1_R_XhoI | ATCATC**CTCGAG**TTACTGAATAATATCATAGTTCAT |
| ScaL2_c3_F_BamHI | ATCATC**GGATCC**AAGCTTGGTGTATTCAATTTGG |
| ScaL2_c3_R_XhoI | ATCATC**CTCGAG**TTAATATTGTGCTACATTATAGTC |
| ScaM1_c4_F_BamHI | ATCATC**GGATCC**GGAAAAGGTGAAGTATATCT |
| ScaM1_c4_R_XhoI | ATCATC**CTCGAG**TTACTAATTGATTGACGCTGCTTG |
| ScaM2_c2_F_BamHI | ATCATC**GGATCC**GCAACACCCACTCCAACAAAG |
| ScaM2_c2_R_XhoI | ATCATC**CTCGAG**TCAATTGATTGATTGTGCTTGAG |
| ScaO_c1_F_BamHI | ATCATC**GGATCC**TACGGCAGCTCAGAAAGCCA |
| ScaO_c1_R_XhoI | ATCATC**CTCGAG**TTACAAAGTCTGATTCAAATTACAC |
| ScaP_c1_F_BamHI | ATCATC**GGATCC**TCTGATGACTTTTTAACTTCAC |
| ScaP_c1_R_XhoI | ATCATC**CTCGAG**TTATAACTTAAGCCCATCATATTGG |
| ScaO_c1+c2_F_BamHI | ATCATC**GGATCC**TTAAAGGTATCTATAGCAGAC |
| ScaO_c1+c2_R_XhoI | ATCATC**CTCGAG**TTAAACACTTGCATCCGAGAACTG |
| ScaO_c3_F_BamHI | ATCATC**GGATCC**AACATTAAGCCTGTTATAACAC |
| ScaO_c3_R_XhoI | ATCATC**CTCGAG**TTATTGCATAAAAGCTTCACCGC |
| ScaR1_c1_F_BamHI | ATCATC**GGATCC**ATTGTAAAAGATACTTTCC |
| ScaR1_c1_R_XhoI | ATCATC**CTCGAG**TTAAACAATTGTTATTGTACCATCC |
| ScaR2_c1_F_BamHI | ATCATC**GGATCC**GCAATAATAAAAGATACCTTTC |
| ScaR2_c1_R_XhoI | ATCATC**CTCGAG**TTAATCTGATGCAACAGGTAGCAC |
| ScaR3_c1_F_BamHI | ATCATC**GGATCC**AAACCTTGCCTATATGTAAGTTC |
| ScaR3_c1_R_XhoI | ATCATC**CTCGAG**TTATCCTGTGAAGGTTACATTGTCG |
| ScaS_c1_F_BamHI | ATCATC**GGATCC**TTGCAGGTAACTATCGGTAGAG |
| ScaS_c1_R_XhoI | ATCATC**CTCGAG**TTATTGTGCTATTATATATCCGTC |
| ScaS_c2_F_BamHI | ATCATC**GGATCC**TACAGCGTGAATATTGAATTG |
| ScaS_c3_F_BamHI | ATCATC**GGATCC**CAATATGATTTGAATATTAAAA |
| ScaS_c3_R_XhoI | ATCATC**CTCGAG**CTCGAGTTATTTTCTTATCTTTACATACC |
| ScaT_c1_F_BamHI | ATCATC**GGATCC**GCTGAACTGAAAGTGGAAATAGG |
| ScaT_c1_R_XhoI | ATCATC**CTCGAG**TTAAATTTCCAGGCTTCCTTCGTC |
| ScaU_c1_F_BamHI | ATCATC**GGATCC**AGTTTAAATGTTGGTTCAGC |
| ScaU_c1_R_XhoI | ATCATC**CTCGAG**TTAAACTTTCCCACTATTCCCTAC |
| ScaVc1_F_XhoI | ATCATC**GGATCC**GTTCAACCTGAAGGATTATAC |
| ScaVc1_R_BamHI | ATCATC**CTCGAG**TTATAACACATAAGCTGGTATTG |
| ScaX1_c1_F_BamHI | ATCATC**GGATCC**GTTTCAGTTTCGATTAATAGCG |
| ScaX1_c1_R_XhoI | ATCATC**CTCGAG**TTATACATCAAAAGTTAACTCAAG |
| ScaX2_c1_F_BamHI | ATCATC**GGATCC**GTTAAAGTAACCGCAGGCTCAG |
| ScaX2_c1_R_XhoI | ATCATC**CTCGAG**TTAACCATCCTCTGTATATATTGG |
| WP_036940956.1_doc_F_KpnI | ATCATC**GGTACC**CGTAACAATCGGTGATGTAAATC |
| WP_036940956.1_doc_R_ BamHI | ATCATC**GGATCC**TTAACCTGCAGGGAAAGATGTTA |
| ScaI_Xdoc_F_KpnI | ATCATC**GGTACC**TGCCGGATTATTCAAACGTAGG |
| ScaI_Xdoc_R_BamHI | ATCATC**GGATCC**TTAGGGTATCTTCGGGTAAACGG |
| ScaV_doc_F_KpnI | ATCATC**GGTACC**CACTGTTTACGGAGATTTTGAC |
| ScaV_doc_R_ BamHI | ATCATC**GGATCC**TTAAGACTGCACTGGAAATATTTTT |
| WP_036945116.1_doc_F_KpnI | ATCATC**GGTACC**CAAAATAGGAGATTTAAACAAGG |
| WP_036945116.1_doc_R_ BamHI | ATCATC**GGATCC**TTAGCCTGCTGGGAAAACAGTAATTG |
| [KNY25939.1](https://www.ncbi.nlm.nih.gov/protein/913265740?report=genbank&log$=prottop&blast_rank=1&RID=6CX4NGUT014)_ Xdoc_F_KpnI | ATCATC**GGTACC**CCAGGGTTTTAAAATTACCGG |
| [KNY25939.1](https://www.ncbi.nlm.nih.gov/protein/913265740?report=genbank&log$=prottop&blast_rank=1&RID=6CX4NGUT014)_ Xdoc_R_ BamHI | ATCATC**GGATCC**TTATGCCGGGTAGTCCGCACTTG |
| [WP_050753119.1](https://www.ncbi.nlm.nih.gov/protein/915007466?report=genbank&log$=prottop&blast_rank=1&RID=6CVAT0RS015)_ Xdoc_F_KpnI | ATCATC**GGTACC**CAGTGGAGCAGGTAATAAGGC |
| [WP_050753119.1](https://www.ncbi.nlm.nih.gov/protein/915007466?report=genbank&log$=prottop&blast_rank=1&RID=6CVAT0RS015)_ Xdoc_R_ BamHI | ATCATC**GGATCC**TTAATAATCTGCTGATGTCTTG |
| ScaL1_doc_F_KpnI | ATCATC**GGTACC**CGTAGTTTACGGTGATTTTGATC |
| ScaL1_doc_R_ BamHI | ATCATC**GGATCC**TTATGCTGATTTCTGGGCAGGAA |
| [KNY27224.1](https://www.ncbi.nlm.nih.gov/protein/913267025?report=genbank&log$=prottop&blast_rank=1&RID=6CP27VU6014)_doc_F_KpnI | ATCATC**GGTACC**CCTACAATACCTAGCTATATG |
| [KNY27224.1](https://www.ncbi.nlm.nih.gov/protein/913267025?report=genbank&log$=prottop&blast_rank=1&RID=6CP27VU6014)_doc_R_ BamHI | ATCATC**GGATCC**TTAATAGGTTTGACCAAACTTC |
| Bc_ScaH1_doc_F_KpnI | ATCATC**GGTACC**CGGAGATGTGCCTGTAAGTGGAG |
| Bc_ScaH1_doc_R_BamHI | ATCATC**GGATCC**TTAAGGTATATAACTTCCGGGCGTAG |
| [WP_050752989.1](https://www.ncbi.nlm.nih.gov/protein/915006705?report=genbank&log$=prottop&blast_rank=1&RID=6CNVDJ0A015)_doc_F_KpnI | ATCATC**GGTACC**CCTTCAAATTCGACAGTGC |
| [WP_050752989.1](https://www.ncbi.nlm.nih.gov/protein/915006705?report=genbank&log$=prottop&blast_rank=1&RID=6CNVDJ0A015)_doc_R_ BamHI | ATCATC**GGATCC**CTAATATGTAAAGCCAAACT |
| [WP_050754083.1](https://www.ncbi.nlm.nih.gov/protein/915013031?report=genbank&log$=prottop&blast_rank=1&RID=6CREC5VK014)_doc_F_KpnI | GTCATC**GGTACC**TGTCCATTCAAGCGGTGA |
| [WP_050754083.1](https://www.ncbi.nlm.nih.gov/protein/915013031?report=genbank&log$=prottop&blast_rank=1&RID=6CREC5VK014)_doc_R_ BamHI | GTCATC**GGATCC**TTATACTGTTGAATTGAATT |
| ScaR3_doc_F_KpnI | ATCATC**GGTACC**TAACAGTAAATTTAATACTG |
| ScaR3_doc_R_BamHI | ATCATC**GGATCC**TTAATTTAATGAGACCAAATC |
| ScaA2_doc_F_KpnI | ATCATC**GGTACC**AGAAGCTGAACAAAGC |
| ScaA2_doc_R_BamHI | ATCATC**GGATCC**TCATTTTTCCTCAGCCGG |
| [WP_050753192.1](https://www.ncbi.nlm.nih.gov/protein/915007896?report=genbank&log$=prottop&blast_rank=1&RID=6CPZNX91014)_Xdoc_F_KpnI | ATCATC**GGTACC**CATTAGCGGGTATGTGAACGG |
| [WP_050753192.1](https://www.ncbi.nlm.nih.gov/protein/915007896?report=genbank&log$=prottop&blast_rank=1&RID=6CPZNX91014)_Xdoc_R_ BamHI | ATCATC**GGATCC**TCATCCCTGTGGAAGAGGAAC |
| ScaL2_d oc_F_KpnI | ATCATC**GGTACC**TACAACGGCATCATCACAAGTG |
| ScaL2_doc_R_BamHI | ATCATC**GGATCC**TTAGAGTAATCATGCTTTCTTC |
| ScaR2_doc_F_KpnI | ATCATC**GGTACC**TAACGGCAGAAATATTGTCATC |
| ScaR2_doc_R_BamHI | ATCATC**GGATCC**TTATTCTATAGTAATTAGTCC |
| ScaR1_doc_F_KpnI | ATCATC**GGTACC**TGAGATTGGGCCAATATATACAG |
| ScaR1_doc_R_BamHI | ATCATC**GGATCC**TTATTCCAAAGTTATTGATCC |
| ScaH3_doc_F_KpnI | ATCATC**GGTACC**CACAGGACCAATTACTGGGG |
| ScaH3_doc_R_BamHI | ATCATC**GGATCC**TTATCCATTAATTGAGTTTGATATAG |
| ScaT_Xdoc_F_KpnI | ATCATC**GGTACC**TATGATGAAACCGGAATTTAAA |
| ScaT_Xdoc_R_BamHI | ATCATC**GGATCC**TTATATATAATCAACTGAAACAC |
| ScaP_doc_F_KpnI | ATCATC**GGTACC**TAACGGATCTGTTGATATATTC |
| ScaP_doc_R_BamHI | ATCATC**GGATCC**TTACTACTGTTGCACAGGGAACT |
| [KNY28903.1](https://www.ncbi.nlm.nih.gov/protein/913268704?report=genbank&log$=prottop&blast_rank=1&RID=6CUFS3A0015)_Xdoc_F | ATCATC**GGTACC**CTCGGTAACTACATTTGTTGG |
| [KNY28903.1](https://www.ncbi.nlm.nih.gov/protein/913268704?report=genbank&log$=prottop&blast_rank=1&RID=6CUFS3A0015)_Xdoc_R | ATCATC**GGATCC**TTACAATGCTGGGTAACTATC |
| [KNY28903.1](https://www.ncbi.nlm.nih.gov/protein/913268704?report=genbank&log$=prottop&blast_rank=1&RID=6CUFS3A0015)_Xdoc_F | ATCATC**GGTACC**CTCGGTAACTACATTTGTTGG |
| [KNY28903.1](https://www.ncbi.nlm.nih.gov/protein/913268704?report=genbank&log$=prottop&blast_rank=1&RID=6CUFS3A0015)_Xdoc_R | ATCATC**GGATCC**TTACAATGCTGGGTAACTATC |
| ScaA1_doc_F_KpnI | ATCATA**GGTACC**AAAAGGCACAGCTACAGTATTA |
| ScaA1_doc_R_BamHI | ATCATC**GGATCC**TTATTTTTGTTCTGCTGGGAAC |
| [KNY25430.1](https://www.ncbi.nlm.nih.gov/protein/913265231?report=genbank&log$=prottop&blast_rank=1&RID=6CREC5VK014)_F_ KpnI | GTCATC**GGTACC**CACTTCAGGTCCAGCAGTTAAAGG |
| [KNY25430.1](https://www.ncbi.nlm.nih.gov/protein/913265231?report=genbank&log$=prottop&blast_rank=1&RID=6CREC5VK014)_R_ BamHI | GTCATC**GGATCC**TTAGGATTTGTTGATACCTGAGTGAC |
| [KNY28540.1](https://www.ncbi.nlm.nih.gov/protein/913268341?report=genbank&log$=prottop&blast_rank=1&RID=6CSA4TKF014)_F_ KpnI | GTCATC**GGTACC**TGCAGAAAAAACACCTGATC |
| [KNY28540.1](https://www.ncbi.nlm.nih.gov/protein/913268341?report=genbank&log$=prottop&blast_rank=1&RID=6CSA4TKF014)_R_ BamHI | GTCATC**GGATCC**CTAGATTGATTTGTTAAACATC |
| [KNY27843.1](https://www.ncbi.nlm.nih.gov/protein/913267644?report=genbank&log$=prottop&blast_rank=1&RID=6CRR57KJ014)_F_ KpnI | GTCATC**GGTACC**TACATCTATATCTACATCTACG |
| [KNY27843.1](https://www.ncbi.nlm.nih.gov/protein/913267644?report=genbank&log$=prottop&blast_rank=1&RID=6CRR57KJ014)_R_ BamHI | GTCATC**GGATCC**CTAGACAATAGCGTTAAATTTTGC |
| KNY26505.1_Xdoc_F_ KpnI | ATCATC**GGTACC**CCCTACCAACGGAATAAAGTAT |
| KNY26505.1_Xdoc_R_ BamHI | ATCATC**GGATCC**TTAATAATCTGAGGAAGTCTTGTT |
| WP_050753227.1_doc_F_KpnI | ATCATA**GGTACC**ATTTGTTAAATTAAAAGGTGAC |
| WP_050753227.1_doc_R_BamHI | ATCATC**GGATCC**TTAAAACATTACGACCTCTGCTGAT |
