# Supplementary material for: Unique organization and unprecedented diversity of the Bacteroides (Pseudobacteroides) cellulosolvens cellulosome system
Source: Biotechnol Biofuels. 2017 Sep 7;10:211. doi: 10.1186/s13068-017-0898-6 (PMC5590126; doi:10.1186/s13068-017-0898-6)
Supplement: Supplementary file 2 — Additional file 2: Figure S1. Hydrolysis of beechwood xylan by cellulosome fractions of Bacteroides cellulosolvens. The two cellulosomal complexes (high-molecular-weight complex and low-molecular-weight complex) isolated from two different growth media (CB and MCC) were tested for their catalytic activity on beechwood xylan in order to demonstrate its ability to degrade it. The Clostridium thermocellum cellulosome (kindly provided by CelDezyner LTD, Rehovot, Israel) was also tested for catalytic activity as a positive control of the catalytic activity of the B. cellulosolvens cellulosomes. CB, cellobiose; MCC, microcrystalline cellulose; Ct, Clostridium thermocellum. [file 13068_2017_898_MOESM2_ESM.docx]

**Additional File 2:**

**Figure S1. Hydrolysis of beechwood xylan by cellulosome fractions of *Bacteroides cellulosolvens***. The two cellulosomal complexes (high-molecular-weight complex and low-molecular-weight complex) isolated from two different growth media (CB and MCC) were tested for their catalytic activity on beechwood xylan in order to demonstrate its ability to degrade it. The *Clostridium thermocellum* cellulosome (kindly provided by CelDezyner LTD, Rehovot, Israel) was also tested for catalytic activity as a positive control of the catalytic activity of the *B. cellulosolvens* cellulosomes. CB, cellobiose; MCC, microcrystalline cellulose; Ct, *Clostridium thermocellum*.
