# Supplementary material for: Unique organization and unprecedented diversity of the Bacteroides (Pseudobacteroides) cellulosolvens cellulosome system
Source: Biotechnol Biofuels. 2017 Sep 7;10:211. doi: 10.1186/s13068-017-0898-6 (PMC5590126; doi:10.1186/s13068-017-0898-6)
Supplement: Supplementary file 3 — Additional file 3: Figure S2. Multiple sequence alignment of 87 cohesin sequences, originating from the genomes of Bacteroides cellulosolvens, Acetivibrio cellulolyticus (Ac), Clostridium thermocellum (Ct), Ruminococcus flavefaciens (Rf) and Ruminococcus champanellensis (Rc). Alignment length: 175; Strongly similar (:): 1 residue =0.57%; Weakly similar (.): 0 residue =0.57%. [file 13068_2017_898_MOESM3_ESM.docx]

**Additional File 3:**

**Figure S2**: **Multiple sequence alignment of 87 cohesin sequences originating from the genomes of *Bacteroides cellulosolvens*, *Acetivibrio cellulolyticus* (Ac), *Clostridium thermocellum* (Ct), Ruminococcus flavefaciens (Rf) and Ruminococcus champanellensis (Rc).** Alignment length: 175; Strongly similar (:): 1 residue =0.57%; Weakly similar (.): 0 residue =0.57%.

**Cohesin name Alignment**

Bc-ScaR3 ---------------LYVSSVTANPGEDVTLKLTMKDMKNV----AGIKANLKYD--SSKLSIK--SIV---------------LDSDLSLNALNK------------------------

Bc-ScaR1 -------------VKVTAGSATTNPNKNVTIKVKMNQMDKV----GGLKIKLTYD--AANLTVN--NVT---------------LAQEFSLANVNT------------------------

Bc-ScaR2 -----------KDVKVSAGSATTSPSKNVTVKVRMNQMENI----GGLQMKLTFD--ANNLAVE--NVL---------------LAPEFSSTAVNS------------------------

Bc-ScaQ_3 -----------------------VAGDVVKLQMAFSGVNTGNDKTNNLMIEFGYD--GSKFELGKG--T---------------ADASLENTGINF-----------NYKYDL-------

Rc-ScaA1 -----KSEAPVGGVVYEIATVEGEAGADVDVPITIKGDTGTA-GM---VLEMSA---DSNLKLK--RRL---------------NGDAYEG-APTW------------------------

Rc-ScaB7 -SETISTTMTTGRVNYQIAEVEAQPGEKVPVPVYVVNDTGTL-GL---TVQFTA---DSRLKYQ--SNA---------------AGNAYTG-MPVW------------------------

Bc-ScaQ_2 -------------------DAAGYPKQKVTVNVDLSNVPSQ--GLTSGELYFEYN--KNVLKLDSF--E---------------KGSVVS-SIEDI-----------DMA----------

Bc-ScaX2 ------------SISVNVSSTSGNPGDEVSVSISLSNIPST--GVNSATLVLEYD--KSKLEFVKF--N---------------SGEIIGDKVRDI-----------SYENIDINAVYTQ

Bc-ScaX1 -------------VSVSINSVKANPGSQVKMEIKFDNIPDK--GLTAAQFNIEYD--KSKVTLKKDNIK---------------SGSIVHNPMFDI-----------I------------

Bc-ScaQ_1 -------------LKVSIADIKTDAGQTISVPLTVENVPAD--GLVAGTVIVNFD--TTKMKFVSA--L---------------NGPIT-NSAKDI-----------GTN----------

Bc-ScaD_2 -------------------DVFCDKGSIVKMIINIEDLPKE--GVKSGQFNIKFD--TNNFKVSKI--T---------------AGEIINDGDKDL-----------SYS----------

Bc-ScaD_3 -----------NAMKISLSKVSCNKGANIKIDVNLSKVPQK--GITSGQFNVEFD--KSKFTVKQI--T---------------VGDIVNDKAKDI-----------SYN----------

Bc-ScaU --------------SLNVGSATGKKLDIIDIPIKYSNIPSS--GVGYFSFTLKFD--KYCLKKK--EII---------------KNSRIINNLAAI-----------DLKDED-------

Bc-ScaD_1 -------------VILSLAKVHGSPGSYINFDVCLQNVPQT--GIATGQLCIYYD--SKNLLLT--PVL---------------GIGVFSGPIIKWEKKGIIVE-NIVFNKKI-------

Ac-ScaH ------------------GSSDAKYKDQVNIPITFKGSPER---ISTLKMTITYD--PTQLEYV--SVS---------------PGEIIPSPVSSF-----------GTQLKS-------

Bc-ScaP -----------------------------SIPLTINNVPTD--GIYNAEAEVSYD--PAKLTVS--NIT---------------AGNAISEGCHLT-----------YTVDSI-------

Ct-CipA3 ------------------DTVNAKPGDTVNIPVRFSGIPSK--GIANCDFVYSYD--PNVLEII--EIK---------------PGELIVDPNPDK-----------SFDTAV-------

Bc-ScaF2 -------------VSISIGSGTGDPEASVKIPVNISGIPSL--GINNIDFELSYD--SNNLEFI--SAI---------------PGAIYDVPTDFS-----------YFNTSE-------

Bc-ScaT -----------AELKVEIGSAGGYPGALVTVPVSFSNVPSS--GINNCDFVLKYD--KSILEAVDDGVE---------------AGPITRNNPVTF-----------DYIIDK-------

Ac-ScaA1 ------------------DSVNGNVGEQIVVPVSFANVPSN--GVSTADMTITYD--SSKLEYV--SGA---------------AGSIVTNPTVNF-----------GINKEA-------

Bc-ScaE_3 -------------VQVSIGSATGEAGSEISIPVSFEDVPNS--GINNCDFKVSYD--SNVLQIK--GIE---------------AGKIVTSPLTDF-----------VANYNK-------

Bc-ScaE_4 -------------IDVAISDVHGEPGSVVTIPVKFSDVPSTG-GINNCDFRLSYD--SSIMDII--EIK---------------AGSIVKSPITDF-----------VVNKSE-------

Bc-ScaF1 -----------GGLKIVVGQVSGNVGDTITVPVTFENVS-M--SVNNCDFLVDFD--DNVLEYV--SVE---------------AGGIVQFPAFNF-----------SCNYAV-------

Bc-ScaE_5 -------------LKVDIENVE-VEGQEAIVPIKFTNVPEL--GINNCDFTLSFD--DKALSVV--SVE---------------AGEIVPFSNVNF-----------SAKGS--------

Bc-ScaE_2 ------------GLKIAVEKVDAVAGQSVTVPIKFENVPSG--GINNCDFKLQYD--NSIFNME--SVI---------------PGSIIANSPSNF-----------YSYTRN-------

Bc-ScaE ----------DSSVNVIIGTVEGSEGDSVTIPVSFQGIT-A--GINNCDFRLSYE--SSFLELE--EIT---------------PGIIVPNAVANF-----------SAYSGR-------

Bc-ScaE_6 -------------LIVDITSIKGVPGDTMDIPVYFRGVPAE--GINNCDFKLMFQ--KGALEIV--SVK---------------AGSIVTLPVANF-----------TSNINA-------

Bc-ScaE_7 ---------IAEGFKVVVGRLNGTAGSTVTVPVTFKGLKGD--GINNCDFRLSYD--TNALEVV--SVD---------------AGLITTLPIVNF-----------TAYTGV-------

Bc-ScaS_1 -------------LQVTIGRVSGKAGQEMVVPITFYNVPKS--GINNCNFTLEYD--TNALEFK--SME---------------AGPIVSLPIANF-----------AYHRR--------

Bc-ScaB_4 -------------VNIEIGKVKVKAGDKIKVPVEIKDIPSI--GINNCNFTLKYN--SNVLKYV--SNE---------------AGTIVPAPLANL-----------SINKPD-------

Bc-ScaB_3 ----------GNKMKIQIGDVKANQGDTVIVPITFNEVPVM--GVNNCNFTLAYD--KNIMEFI--SAD---------------AGDIVTLPMANY-----------SYNMPS-------

Bc-ScaB_2 -------------------------GTEVNVPITFENVPDN--GINNCNFTLSYD--SNALEFL--TTE---------------AGNIIPLAIADY-----------SSYRSM-------

Bc-ScaB_5 ----------LYNLNVNIGEISGEAGGVIEVPIEFKNVPDF--GINNCDFSVKYD--KSIFEYV--TYE---------------AGSIVKDSIVNL-----------ACMEN--------

Bc-ScaB_1 -------------VNIIIGSAQGIPGSTVKVPINLQNVPEI--GINNCDFTIKFD--SDILDFN--SVE---------------AGDIVPLPVASF-----------SSNNS--------

Bc-ScaS_2 -----------YSVNIELGKVNGEAGSEITVPVKFNNLPPM--GINNCDFIIGYD--TEGLEFK--SVQ---------------PGDIVTYPLGCF-----------NYNKSQ-------

Bc-ScaS_3 ----------QYDLNIKIGKVAGAAGREVVVPITFDKIPSI--GINNCDFILDYD--STALELK--RIE---------------AGDIVPDSSRNF-----------SYNKPF-------

Bc-ScaB_9 ----------VYKMNVVIGRVNVVAGEEVVVPVEFKNIPAI--GVNNCNFVLEYD--ANVLEVK--KVD---------------AGEIVPDALINF-----------GSNNSD-------

Bc-ScaB_10 ----------LYSMNVIIGKVNAEASGEVVVPVEFKDVPSI--GINNCNFILEYD--ASALELD--SAE---------------AGEIVPVPLGNF-----------SSNNKD-------

Bc-ScaB_6 -----------YWMNVLIGNMNAAIGEEVVVPIEFKNVPPF--GINNCDFKLVYD--SNALELK--KVE---------------AGDIVPEPLANL-----------SSNKS--------

Bc-ScaB_7 ----------MYWMNVVIGKMNAEVGGEVVVPIEFNNVPSF--GINNCDFKLVYD--ATALELK--NVE---------------AGDIIKTPLANF-----------SNNKSE-------

Bc-ScaB_8 ----------MYWMNVVIGKMNAEVGGEVVVPIEFKNVPSF--GINNCDFKLVYD--ATALELK--NVE---------------AGDIIKTPLANF-----------SNNKSE-------

Rf-FD1-ScaA1 ---TSAQPVANADVIFDFGNYEAKAGEEVQVDVTVDSKNKA---ISAMDVVFAIDS-PLTIDEIDK--ESLAFKTTAMTNMAILGANFKSLDDK-----GEPLVPTK---DP----VFTL

Bc-ScaI ----------------SYNRT-------PIFDINGYYL-KN---IASYSFKLNYRYTAQIIGK----------------------------ELTGSP-NDNLLA------NL----QYDP

Bc-ScaA2_10 ----------DGSVKMTFDKEKVKVGDMVKATVSIDGI-NK---LTGFQFNIKYD--KNYLQPWDT--Q---------------SDS--PYKTATIPDLSTILT------NE----KYIP

Bc-ScaA1_11 ---------VDGSVLTAIDNDKVAVGDKVTLTINVDKI-TN---FSGYQFNIKYN--TTYLQPWDT--I---------------ADE--AYTDSTMPDYGTLLQ-------G----RFNA

Bc-ScaG --------IDTPEVYITLDKNNAEVGDIVKATVNIKNF-YG---FAGFQVNLKYD--PEVIQPIDT--A---------------SNA--AFTDTSLPDEGTLLK-------G----KFSP

Bc-ScaA2_2 ---------NKAEVYMTVDKTNAAVEDIITATLNVKGF-IG---IVGYQACVKYD--PAVLMPVDE--N----------------GV--PYDNTTLPGYGTIFK-------K----GSKL

Bc-ScaJ ---------VNSQFILEADQTNIAKKDVIEVSLSVKDM-PN---FAGYQANIKFD--PKVLRPIYS--D----------------AT--PFDNNSAPETGELLL-------K----RYSP

Bc-ScaL2_3 ---------KLGVFNLVVDKTDAPVGDIITTTLSIKEI-PN---FSGYQATLKYD--PDCLQPVYS--D----------------GT--PYDKTSAAETGSLLC-------G----RYSP

Bc-ScaA2_6 ---------GSGEVYVTLDKTNQSVGDVITASINLKGF-KT---IAGFEANIKYD--PSALMPVYG--D----------------GT--PYDSASMPESGTLFN------NK----KYMP

Bc-ScaA1_1 ----------APSISVTLDKTTASVGDIITATISANGI-QN---FAGYQANVKYD--PAVLQPVYS--A----------------TE--PYDGSSVPEYGTLLQ-------K----RYSP

Bc-ScaA1_6 ---------IDGTVELSLDKTTAGVGDIITATLKINKI-PG---FAGYQANIKYD--PAALTPVYS--D----------------GT--AYDDAAVPEYGDLLQ-------K----RYSP

Bc-ScaA1_2 ----------DSGVTVEVDKTDAKVGDIITATINVKNI-NG---FAGYQANLKYN--PAVLQPVYE--D----------------GT--AYDNASAPEYGKLLQ-------K----RYSP

Bc-ScaA1_3 --------LTGSGVEATVDKTTASVGDIITYTISVKDI-AG---FAGYQANVKYD--PSVLKPVYE--D----------------GT--EYDNAAVPDYGKLLQ-------K----RYSP

Bc-ScaA1_4 ---------IGSGVTATVDKTTASVGDIITYTINVKDV-AG---FAGYQANVKYD--PSVLQPVYD--D----------------GS--AYDSAAVPEYGTLLQ-------K----RYSP

Bc-ScaA1_5 -------QIPGSGVVATVDKTTASVGDIITYTINVKDV-AG---FAGYQANVKYD--PAVLQPVYD--D----------------GT--AYDSAAVPEYGTLLQ-------K----RYSP

Bc-ScaA2_4 ---------YKAEVYMTVDKTTADAGDIIKATLNIKGFSDG---ISGYQANIKYD--PTVLMPIYS--D----------------GS--LYDEKAPAEYGTLLQ-------Y----RFGP

Bc-ScaA2_3 ---------CKGELYVSVDKTTATVGDIITATLNVKGF-DS---IAGYQVCMKYD--RSVLVPVYE--D----------------GV--RYEKDSVPEYGTLMQ-------K----KFSP

Bc-ScaA2_7 ---------SKGEVYMTLDKNNAAVGDIITATVGVKDI-AC---LAAYQVNIKYN--PSAFQPVYE--D----------------GT--PYDNKSVPEYGTLLQ-------K----RFSP

Bc-ScaA2_5 ---------KDAGVYLLLDKTNVSVGDIIKATIYADGF-EI---IAGYQATVKYD--PSVLQPVYP--D----------------GS--SYDGYSVPEYGTLLQ-------K----RFSP

Bc-ScaA2_8 ---------KKGTIYLTLDKSNAVVGDIVKATVNVKDF-DC---IAAYQANIKYD--PTMLQPVYF--D----------------GT--VYDETSVPEYGNLLQ-------K----RYSP

Bc-ScaA2_9 ---------CKGSISLQLDRSYAAVGDIIKATIDVKDF-DC---VAGYQASVKYD--PTVLQPVYS--D----------------GT--PYDNSSVPEYGNLLQ-------K----SYSP

Bc-ScaA2_1 ---------SKGEVYLTVDKTFAAVGDVITATLNIRYF-EA---VAGYQANIKFD--PTVLVPIYA--D----------------GT--PYDRKSFPEYGTLLQ-------K----RYNA

Bc-ScaA1_8 ---------TKGEVFVTLDKTKAAVGDIITATINVKDF-DS---VAGYQASVKYD--PAVLQPVYS--D----------------GT--AYDNSSVPEYGKLLQ-------K----RYSP

Bc-ScaA1_9 ---------TKGEVFVTFDKTTAAVGDIITATISVKDF-DS---VAGYQASIKYD--PAVLQPVYS--D----------------GT--AYDNSSVPEYGKLLQ-------K----RYSP

Bc-ScaA1_10 ---------TKGEVFVTLDKTTAAVGDIITATINVKDF-DS---VAGYQANIKFD--PAVLKPVYS--D----------------GT--AYDNSSVPEYGKLLQ-------K----RYSP

Bc-ScaA1_7 ---------TKGEVFVTLDKSTAAAGDIVTATINVKEF-DV---ISGYQANIKYD--PSALQPVYS--D----------------GT--AYDSSSVPESGDLLQ-------K----RYSP

Bc-ScaM1_1 ---------SKGEVFVTVDKTTAAVGNIIKATINVKDF-DV---IAGYQASLKYD--PTVLQPVYL--D----------------GT--QYDNSSTPEYGTLLQ-------K----RYSG

Bc-ScaM1_2 -----------AEVYVTVDKTTAAVGDIIKTTISVKGF-DV---VAGYQANIKYD--PTVLQPVYL--D----------------GT--PYDNSSSPEYGTLLQ-------K----RYSG

Bc-ScaM1_3 -----------AEVYVTVDKTTAAVGDIIKATISVKGF-DV---VAGYQANIKYD--TTVLQPVYL--D----------------GT--PYDNSSSPEYGTLLQ-------K----RYSG

Bc-ScaM2_1 ------GTTSKGEVYLSVDKVKASVGDIIAATLSAKNF-DN---VSGYQANIKYD--PSVLQPVYL--D----------------GT--PYDNTSSPELGTLLN-------K----RYNG

Bc-ScaM1_4 ----------KGEVYLTLDKTTAAVGDIIAATLNVKGF-DV---VSGYQANIKYD--PSVLQPVYL--D----------------GT--PYDSSSAPDYGTLLQ-------K----RYSG

Bc-ScaM2_2 ------GQTSKGEVYLSVDKTKAAVGDIIAATLNVKGF-DN---VSGYQANIKYD--PSVLQPVYL--D----------------GT--PYDNASSPELGTLLN-------K----RYSG

Bc-ScaH3 --------AINPSISMEHSTICPIAGQYIQTKIKVKDI-YG---FAGYQVRIKYD--KTLLEPYVQ--Y---------NDSAIYTGNYEPMDNYTKPNPGDILLHSRECKKS----DFIP

Bc-ScaL1_2 ---------AAPGLFMNSEKVPS-SNGLLKVTLNVSNI-KN---LAGYQVNLKYN--PEILLPVNM--D---------------GSE---FNDSSNIEPGTLFK------NN----DYSP

Bc-ScaV --------VQPEGLYISYSKEP---LNMLKVTLNIKNI-AN---FSGYQANLVYD--PQVLRPVYS--D---------------GSE---YDSQSPVEIGSLLT-------K----RYIP

Bc-ScaL1_1 -------ISSGPELYMIASNES---SNIYKVTLNIKNI-ER---FSGYQANLVYD--PKVLKPINL--D---------------NSE---FTSQSPVEAGSLLT-------K----KYSP

Bc-ScaW1 --------DSLPSIGIELDKTEAKVGDIIKATIKANDI-KN---LNGFQLNMRFN--QEVLKIVSE--D---------------GSE--SFDDKTCPENGELIM------NK----NYSL

Bc-ScaO TYKLSVYDENVGTASMESDKSTANVGDYITLSIKADTI-TN---LAGYQVNLKYD--PSVVMPVDY------------------TNT--PFDTSTYPMCGNILV------NP----KYSP

Bc-ScaW2 --------DNQPSIFIEFDKTKASVGETIKASVKINNI-KD---FGGYQVRLRYN--PKVLQAVDA--D---------------NGT--PLENKTPPTKGDLLQ------NK----DYSP

Ac-ScaE2 ------------------DKTSAAVGDIVKATLSVNNI-EN---FAGYQVNLKYD--PAVLQPVTA------------------SGT--PFSKSTVPSGATILT------NE----DYYK

Bc-ScaH1 -------IMPTGKIKMQVDKTIANVGDIVTATVSIQDI-SN---LIGYQINIKYD--PAVLQPVI-------------------DGI--PYTNSTFPTKGTILS------NQ----TYSP

Ac-ScaB1 ------------------DKTTASVGEIVTASINIKNI-TN---FSGCQLNMKYD--PAVLQPVTS------------------SGV--AYTKSTMPGAGTILN------S-----DFNL

Ct-Orf2p1 ------------------DKTKANIGDIIIATIRIDNI-NN---FSGYQLNIKYD--PSYLQAVNP--L---------------TGEPIKKRTMPAVNGTVLLK------GD----QYSI

Bc-ScaH2 -----ADTAEKGTLTLSVDKTSAIQGDIITATLSIKNI-KN---FAGYQVNMMYD--PKVLQPIIP--F---------------GDDFLPYLNLTAVEPGTLLA------NS----KYSP

Bc-ScaL2_1 -------ADIKGSLTMSFNKNHADQGDIIIASLNIKDI-KD---FAGYQVNIKYD--ATVLQPIIP--F---------------GDDYLPYGNLTPAEPGTLLA------KE----EFKP

Bc-ScaL2_2 -------ADINGSLTMSFNKNHADQGDIIIASLNIKDI-KD---FAGYQVNIKYD--ATVLQPIIP--F---------------GDDYLPYGNLTPAEPGTLLA------KE----EFKP

: .

Bc-ScaR3 ---------------NLSGEI-----YFNGLNSEG----ITKDLLDIATVTFTVNSRIPA-KLMPI---DLPIKI---------ASIDA-------CDVNAQNIS-ISCNDGKITVVPPV

Bc-ScaR1 ---------------NIPGEI-----LFNGINSDG----ITNSSIDIATITFKARSDIDV-STLPM---NISLKV---------ASAEA-------CNLDFQEIG-PIYTEDG-------

Bc-ScaR2 ---------------NIPGEI-----LFNGINSEG----LTNSSMEIAAITFKVNSNLDV-NIL--------------------------------------------------------

Bc-ScaQ_3 -----------GTGNDKKLKA-----IYLDMD--KE--TALTDGKVIYTAYLKIKDTTPV-DTYKL---AFSAIK---------MIDRTTG--------KVYTVN-----SGEAFMQ---

Rc-ScaA1 --------------NKDTLTY-----VWNAGD--GR-NLVAADGAVLTTLKFTVPADAQPGDEYPI---SFRSDL---------CKVIDQ---------EGVELN-ITYVDGMIKIPGEA

Rc-ScaB7 --------------DAASYTL-----NWSLDT--AE---TAKSGAVLTTLTYKVPDDAQPGDRFPV---EFVVEN---------CSTVDE---------NGNALT-VGYFNGAIYIPDPD

Bc-ScaQ_2 ------------DDYSKGAVL-----LYTDSQNTGASNTHILANGRLVSVTFLIQDGSPK-GAYSI---KISAPP---VSVGGIPFYSMQ----------TYPVK-AQFSDASV------

Bc-ScaX2 EQLAMIRNDSTKPPPQDGVKI-----LYNDDAQTGD--SHIKRNGIFGEAVFKIKSSFTS-G----------------------------------------------------------

Bc-ScaX1 ------S-----NDIDTGTTI-----IYTDYDMTGN--SHIKSSGTFLELTFDV------------------------------------------------------------------

Bc-ScaQ_1 -------------FDGKSLIV-----LYADDAQSGT--SHIKTNGVLATLTFTVNADCPN-GAYPI---SLANDG-------ENPTF---------------------------------

Bc-ScaD_2 -------------LISNGLTV-----LYSDSKQSGN--SFINKSGVFCIINLEVSASCPY-GEYDF---KFYKDD-------NSCFYSDY----------EV------------------

Bc-ScaD_3 -------------TIENGITV-----LYSDDKQSGD--SYIKKDGVLCTIEFDVSASCPA-GEHDI---KFYKDE-------SSVFYSEM----------ISLVE-VLYEDGKITVSN--

Bc-ScaU -------------GDVCGIEF-----SFFS---LQE--DVLNNNEEFAKIRYEIIDNSNS-DLTI------EDLK---------VWTYIYNSSK--RRYELSKID-CVGNSGKV------

Bc-ScaD_1 ----------------VGLRI-----LYDG----RE--NFIRDNGVFLTLKFKVNDECLEKQIITV---DSEGEI---------PFYTKSNNES--NPLELRPINNVEYRAGEITIG---

Ac-ScaH ---------------PGEIEL-----FFMNYTCCTD--YDIRAEGMFANLTFNVVSSSNVTAAVNA----KDVLI---------ADG------------CINPVP-TTIYPG--------

Bc-ScaP ---------------IGRIKV-----IFTGDM-VFQ--KLIKTNGIFANIEFDVFDSVNVIGSTPL---TLER-----------------------------------------------

Ct-CipA3 ------------YPDRKIIVF-----LFAEDSGTGA--YAITKDGVFATIVAKVKSGAPN-GLSVI---KFVEVG---------GFANND----------LVEQR-TQFFDGG-------

Bc-ScaF2 -------------NGVGKIKF-----LFSDTT-QGS--QPLKKEGVMVELNFKVKKSAVQ-GVYKI---QKYEIG---------SCSSIDSA-----NKKLVPVN-VNFNDGAITVTN--

Bc-ScaT --------------NNGTIKF-----LYCDETGLGN--EAIKSNGVFANIRFLIKQNAYA-STYKI---KPTGEF---------AFGEN----------GLEYIP-ASIDEGSLEI----

Ac-ScaA1 ---------------DGKLKV-----LFLDYT-MST--GYISTNGVFANVTFKVLNSAPT-TVGIT--------G---------ATFGD--K-------NLGNIS-ATINAG--------

Bc-ScaE_3 ---------------AGIVQF-----LYNDNT-QGS--NLISSNGVFANIKAVIKNDAKA-GSYDI---KVTSLG---------AFSSKN----------MVSVK-VGTGNG--------

Bc-ScaE_4 ----------------DTISF-----LYNDIT-QGS--NSIVDDGVFANITLKIKENAPA-GNYSF---KFKRIG---------SVSGKN----------MVKVN-VTTKEGVLTVD---

Bc-ScaF1 ----------KDFADNKTINF-----LFSDLT-QGT--MNIIASGVFANIMFKIKANAKE-VISPI---EVVKIG---------AFSGKPSK----PGQN--------------------

Bc-ScaE_5 ---------------NGTVKF-----LFNDNT-QGQ--FLITKDGVFANIKFKVLNPSVT-GNYPI---SLNRVG---------AFSCAG----------MVRLE-PVFTDGKIIVTAIK

Bc-ScaE_2 ---------------DGTVYF-----LFSDST-QGS--NQIKNDGVFASVKLKVKESAAV-KEYKL---VSSKVG---------AISGLKED----NSYKMMPID-AFIDGGVIAVTKSG

Bc-ScaE ---------------PGVISF-----LFNDST-QGL--LPITINGPFANLKFKIKDNAVS-GNYEV---KLNSSG---------SFSNSRGG----------SIN-EKFSSGSIKVNGNV

Bc-ScaE_6 --------------EGGYVSL-----LFSDAT-QGQ--MPIINDGEFAVIKVKIKDSAAS-GNYKF---ALSHVG---------SFSSLKDD-------KMSAIL-PSFNEGKIT-----

Bc-ScaE_7 ---------------KGVISF-----LFNDAT-QGS--MPIKENGVFANITFKLKSDISN-GSDII---KLNKMG---------SFTGTDRK-------AIDTT--ISFE*---------

Bc-ScaS_1 ---------------DNIINT-----VFSDES-LGS--LQIVSDGAFAYIKFMIKKDIPG-GIYDI---KLSGIA---------TFSCLNGM-------KLKSIP-TVFTDGYIIAQ---

Bc-ScaB_4 ---------------EGIIKL-----LFSDAS-QGG--MPIKDNGIFVNLEFQAVNDANI-GVYGL---ELDTIG---------AFSGISSA-------KMTSIE-PQFNNGSIEIFNSA

Bc-ScaB_3 ---------------DGLVKF-----LYNDQA-QGA--MSIKEDGTFANVKFKIKQSAAF-GKYSV---GIKAIG---------SISALSNS-------KLIPIE-SIFKDGSITVTNKP

Bc-ScaB_2 ---------------EGKIKF-----LFSDSS-QGT--RSIKNDGVFANIKFKIKGNAIR-DTYRI---DLSELG---------SFSSKQNN-------NLKSIA-TQFLSGSVNVKDIE

Bc-ScaB_5 ---------------SGIINL-----LFNDAT-QSS--SPIKNNGVFAKLKFKINSNAAS-GTYQI---NAEGYG---------KFSGNLNG-------KLTSIN-PIFENGIINIGNVT

Bc-ScaB_1 ---------------KDIIKF-----LFSDAT-QGN--MPINENGLFAVISFKIKDNAQK-GISNI---KVSSYG---------SFSGMSGK-------EMQSLS-PTFFSGSIDVSDVS

Bc-ScaS_2 ---------------DGSINF-----LYNDET-QGA--MPIVKNGVFAEITFKIIDNAKT-GIYGV---SKISVG---------LFSGFVNE-------KLR------------------

Bc-ScaS_3 ---------------DGQIYM-----LFVDQS-QSN--MPIKRNGVFAKVIFKVKDNAPS-GTYYV---NKQHVG---------SFSGSLGS-------KLAPIN-AKFSNGYVKIRK*-

Bc-ScaB_9 ---------------EGKVYF-----LFNDAL-QGR--MQIANDGIFANITFKVKSSAAA-GIYNI---RKDSVG---------AFSGLVDK--------LVPIS-AEFTDGSISVESAK

Bc-ScaB_10 ---------------EGKIYF-----LFSDGT-QGR--MQIVNDGIFAKIKFKVKSTASD-GTYYI---RKDSVG---------AFSGLIEK-------KIIKIG-AEFTDGSITVRSLT

Bc-ScaB_6 ---------------EGKIQF-----LFNDAS-QGS--MQIENGGVFAKITFKVKSTAAS-GIYNI---RKDSVG---------SFSGLIDN-------KMTSIG-PKFTDGS-------

Bc-ScaB_7 ---------------EGKISF-----LFNDAS-QGS--MQIENGGVFAKITFKVKSTTAT-GVYDL---RKDLVG---------SFSGLKDN-------KMTSIG-AEFTNGS-------

Bc-ScaB_8 ---------------EGKISF-----LFNDAS-QGS--MQIENGGVFAKITFKVKSTTAI-GVYDI---RKDLIG---------SFSGLKDS-------KMTSIG-AEFTNGSITVA---

Rf-FD1-ScaA1 --YVTVPA----TTPDGVYNVGFGKKCEVHKSND----GSKYSS-TAINGKIKVGNPVDD-PTTSA------------------------------------------------------

Bc-ScaI --VVETNN----DTDNKILSINKSYTKLADYKNS----GAGEESGRLISIQADIPFVNR-----AI--------GSDIFQINEFTCKDWDNNVIKVEVLK--------------------

Bc-ScaA2_10 --LSIAAN----DIASGILNFGKTYLDISSYKGD----E---TSNQSVVITFKVINKIP---EGGAKLAWFEHSDTMGNDVNGTLVFAADSKPLTSDNYKV--ISPASIY----------

Bc-ScaA1_11 --TDMSKH----NLSQGVLNFGRLYMNLSAYRAS----GKPESTGAVAKVTFKVIKEIP---AEGIKLATFENGSSMNNAVDGTMLFDWDGNMYSSSAYKV--VQPGLIY----------

Bc-ScaG --TAMANN----DVANGTLTYGSAYINMEAYKKS----GKSESTGSVAVFNFKVLKKQQ----TTL---NFQNSSAMTNAKNGTMLFNWDGTSL--TNYKV--KGAAELNK---------

Bc-ScaA2_2 --SELASN----DLVKGLLNFGRCYFPMNTLVAS----DY-GSTGSIAVIRFKVLKNAS----TAI---TLVNVPSLTNPIDGTMIFDWDGAQL--SNYKV--SQAPSINS---------

Bc-ScaJ --TDMAAN----DLAKGTLTFGRTYMNLTGYKAN----NTPESTGTIAYIYFEVLESKN----TKI---ELQDSASLTNAVSGTMAFDWDGAQQ--SNYTV--VGTVELIGQAQEQS---

Bc-ScaL2_3 --TDLASN----DLESGLLNFGRAYMALNTYRNS----KAPESSGILAKVHFKVLKPYT-----KP---ALVDNSLLDNDIEGTLVFDWNGNIV--TDYNV--AQY*-------------

Bc-ScaA2_6 --SDAADN----DLSKGTLTFGKSYMDISTYRKS----GNAENDGTVAIVRFKVLKSSP----TAI---KLEDAAAMTNSISGTMVFDWDAKQL--SGYKV--TQAPLINN---------

Bc-ScaA1_1 --TDMGAN----DLANGSLTFGRTYMNLSGYMNS----GSSESTGTLAIIKFKVLKKAS----TQI---KLQNAASLTNAVDGTMVFDWTGAQL--SNYSV--EQAPVLND---------

Bc-ScaA1_6 --TDMAAN----DVAKGTLTFGRTYMALDSYKAS----GSAETTGSIAVIRFKVLKSTG---STSI---KLENAASLTNAVDGTMLFDWTGAQL--GGYTV--KQASTIKL---------

Bc-ScaA1_2 --TDMASN----DITKGTLTFGRTYMNLDSYKNS----GAAEKEGSIAVIRFKVLKVEA----TTI---TLQNAASLTNAVDGTMLFDWTGAQL--AGYRV--QQAPSING---------

Bc-ScaA1_3 --TDMASN----DLSKGTLTFGRTYMNLDSYKAS----GSAETSGTIAVIRFKVLKNTA----TTI---KLQNAASLTNAVDGTMLFDWSGAQL--AGYKV--AQAPSING---------

Bc-ScaA1_4 --TDMASN----DLSKGTLTFGRTYMNLDSYKAS----GSAETTGSIAVIRFKVLKNTA----TTI---KLQNAASLTNAVDGTMLFDWSGAQL--AGYKV--AQAPSING---------

Bc-ScaA1_5 --TDMASN----DLSKGTLTFGRTYMALDSYKAS----GSAETTGSIAVIRFKVLRSTA----TAI---LLQNAASLTNAVDGTMLFDWTGAQL--KGYKV--AQASSING---------

Bc-ScaA2_4 --IDVASN----DLVNGTLTFGRGYMNMPEYRNS----GIIESEGSLAVIRFKVLKAAP----VSI---TMENGPACLDAISGTMIFGRNNLRN--NDYTV--TQAPAINS---------

Bc-ScaA2_3 --VDSAFN----DFENGCLNFSRVYMNIEKYRNS----GVPDQEGSLAVVRFKVIRAAS----TKI---SLVDSPALTNAVDGTMVFDWNAAQL--SNYKV--TQAPEISS---------

Bc-ScaA2_7 --TDIVNN----DLKNGILNFGRAYMSMSQYKNS----GTAETTGSLAVIRFKVLKADK----PVI---VFEDSSTMSNQVTGTMVFDWDGNQL--TGYKV--SQPQSINI---------

Bc-ScaA2_5 --VDMALN----NVSKGILTFGRSYMDMASYRKS----GVPENKGSLAVIQFKVLKSAY----TNI---ALENALGFPNTESGTMMFDWNSTQL--KGYKV--INQPIIAI---------

Bc-ScaA2_8 --IDMAVN----DLNKGILNFGRSYMALSLYKNS----GVPEREGKLAIVGFKVLKAGK----TNI---LLDNTSFMQNAISGTMVFDWDGYQL--SGYKV--LQAPALNI---------

Bc-ScaA2_9 --TDMGAN----DLKKGILSFGRTYMNLDAYKKS----GVPEQNGTLAVICFKVLKASP----ANV---IFYKSPSMPNAVEGTMIFDWDGYQI--LGYRV--IQSMAVIP---------

Bc-ScaA2_1 --IDLAAN----DLTKGILSFGREYMSMDIYRNS----GVAENTGSIAVIKFKVLKNTP----TQI---KFEDSPILTNAVNGTMMFDWYGKQL--SNYIV--TQAPEINS---------

Bc-ScaA1_8 --TDMGAN----DTANGILTFGRTYMNLSGYKAS----GVAEKEGSIAVISFKVLKATE----TKV---LLKNVPSLTSPVDGTMVFDWDGVQL--ANYKV--TQAPVVNG---------

Bc-ScaA1_9 --TDMGAN----DTANGILTFGRTYMNLSGYKAS----GVAEKEGSIAVISFKVLKATE----TKV---LLKNVPSLTSPVDGTMVFDWDGVQL--ADYKV--TQAPAVNG---------

Bc-ScaA1_10 --TDMGAN----NLENGILTFGRTYMNMAGYKAS----GVAEKEGSIAVISFKVLKATE----TKI---ELTNVPSLTNPVDGTMVFDWDGTQL--ANYKV--TQAPAVNA---------

Bc-ScaA1_7 --TDMASN----DLTKGTLTFGRTYMNLASYKTA----GVKENTGSIAKISFKVLKSGP----VSI---LLADSPSLTNAITGTMMFDWDGVQL--DNYKV--TQAGGSTT---------

Bc-ScaM1_1 --TDMAAN----DLSKGILTFGRTYMALDSYKAS----GVKENNGSLAIIGFKVLKTTS----TRI---TLENTPSLTNPVVGTMVFDWNGTQL--NGYTV--TQAA-------------

Bc-ScaM1_2 --TDMASN----DITKGILTFGRTYMALESYKAS----GVKENTGSLAIIGFKVLKVSS----TKI---MLENASSLTNPVSGTMVFDWNGTQL--NGYTV--TQASTINS---------

Bc-ScaM1_3 --TDMASN----DITKGTLTFGRTYMALESYKAS----GVKENAGSLAIIGFKVLKVSS----TKI---MLENASSLTNPVSGTMVFDWNGTQL--SGYAV--TQAAAINS---------

Bc-ScaM2_1 --TDMASN----DLNNGKLTFGRTYLSLDAYKAS----GVKENTGSLAVIGFKVLKAVS----TKL---TLEDAPTLTNSVGGTMVFDWDGKQL--AGYAV--TQNVEINSGN-------

Bc-ScaM1_4 --TDMAAN----DLSKGSLTFGRTYMNLASYKNS----GVPENTGS--------------------------------------------------------------------------

Bc-ScaM2_2 --TDMASN----DLSNGKLTFGRTYMALASYKAS----GVKENTGSLATIGFKVLKVTS----TKI---TLENSPSLTSPVVGTMLFDWDGQQL--AGYAV--TQAQSIN*---------

Bc-ScaH3 --VSIAKN----DISNGILNFGRYYIKASAYKSS----GISESTGTIAVIYFKVLKNSVN-PLTSI---SFENSST--------------------------------------------

Bc-ScaL1_2 --LKLANH----DLANGTLNFGTAYIDVNSYKKS----TTLKTSGTIAVIYFTVLKHVP----TEI---ELTDCETMPLAVSGTILYNWDCSQV-FE-YSV--VKSLKIEPSSMTIPVPA

Bc-ScaV --TDFAKH----DLQNGVLNFGRTYLAVDSYKNS----GVAESSGSIAVIYFKILKYET----TKI---RLENCDTMPGALKGTIITDWDGNMI-LD-YNV--GETSTIPAYVL------

Bc-ScaL1_1 --IDFAMH----DLQNGVLNFGRAYIAISSYKSS----GVSESSGTIGVIYFKMLKSEA----TQI---LLENCSTMPEAVSGTIISDWNGDLV-LN-YSV--TSKISLVPSNTSVPSNT

Bc-ScaW1 --FPAAEN----IISAGVLNFGRTYIDMGSYRQS----NKPESTGSLAVLYFKVIKDAA----TEI---VFEELGTMPGSNKGTLLFDWDGNTI-KE-YSV--NDSTKIN----------

Bc-ScaO --FPSAMN----NINSGILNFGTCYMNMDDYRTA----GMAENTGTLAVVRFKVLRANQ----IKI---QFENTPSMPNSPNGIAFYDWYGNAL-KCNLNQ--TL---------------

Bc-ScaW2 --MGLTTN----IIEDGILDFGNLYMNMIDYKKS----GKAETTGTLAVIGFKVLQDAA----TEV---IFDDVNGNPGAINGVMLFDWTGAQL-ASGYSI--VNAP-------------

Ac-ScaE2 --YPMASH----NISEGILCFSNTYTNLEDYKAS----GIAETTGTLAVISFKVLQGKA----TTV---SFEDSRTMPNGITGTILCNWNAERI-SG-----------------------

Bc-ScaH1 --FDLVDN----KLINGVLNFSSAYLCMAKYRQN----AQPETSGTLAVINFKVLNNTP----THI---KFEGYKSMPRAILGTYLYDWNGATY-NSGYSV--IQPQRIN*---------

Ac-ScaB1 --RQVADN----DLEKGILNFSKAYVSLDDYRTA----AAPEQTGTVAVVKFKVLKEET----SSI---SFEDTTSVPNAIDGTVLFDWNGDRI-QSG----------------------

Ct-Orf2p1 --TEVVEN----NVDEGILNFGKGYANLTEYRKS----GKPETTGIIGKIGFKALKLGK----TEI---KFENTPVMPGAKEGTLLFDWDAETI-TE-----------------------

Bc-ScaH2 --VDLVFH----DVEIGLLSFGRSYVQLASYKNS----ANVESTGSIGVIRFRVLKVLP----TEI---YFKDTKVLPNGFVGTSIFDYNGAQT-TY-YDI--IQPGKIFTGSSS-----

Bc-ScaL2_1 --VDIVFH----DTEIGLLSFGRSYLKLDAYKNS----GNIETTGSVGIIRFRVLRVIP----TKV---YFKGTKILPSGIQGTALYDFNANQI-MN-YDI--IQ---------------

Bc-ScaL2_2 --VDIVFH----DTEIGLLSFGRSYLKLDAYKNS----GNIETTGSVGIIRFRVLRVIP----TKV---YFKGTKILPSGIQGTALYDFNANQI-MN-YDI--IQ---------------

**Accession numbers:**

>Bc-ScaA1_ [WP_050753331.1](https://www.ncbi.nlm.nih.gov/protein/915008729?report=genbank&log$=prottop&blast_rank=1&RID=6HW2KDYV014)

>Bc-ScaA2_[WP_036943826.1](https://www.ncbi.nlm.nih.gov/protein/739072496?report=genbank&log$=prottop&blast_rank=1&RID=6HWFFJBY014)

>Bc-ScaB_ [WP_050753330.1](https://www.ncbi.nlm.nih.gov/protein/915008724?report=genbank&log$=prottop&blast_rank=2&RID=6M7JHB1V014)

>Bc-ScaD_ [WP_036944733.1](https://www.ncbi.nlm.nih.gov/protein/739073418?report=genbank&log$=prottop&blast_rank=1&RID=6M93NX2M014)

>Bc-ScaE_ WP_050753599.1

>Bc-ScaF1_ [WP_050752983.1](https://www.ncbi.nlm.nih.gov/protein/915006674?report=genbank&log$=prottop&blast_rank=1&RID=6M91NUSC014)

>Bc-ScaF2_ [WP_036940409.1](https://www.ncbi.nlm.nih.gov/protein/739069020?report=genbank&log$=prottop&blast_rank=1&RID=6M95XZMF014)

| >Bc-ScaG_ | [WP_036938069.1](https://www.ncbi.nlm.nih.gov/protein/739066661?report=genbank&log$=prottop&blast_rank=1&RID=6MATC7DZ014) |
| --- | --- |

>Bc-ScaH1_ [WP_036937628.1](https://www.ncbi.nlm.nih.gov/protein/739066219?report=genbank&log$=prottop&blast_rank=1&RID=6MAET52S014)

| >Bc-ScaH2_ | [WP_036945220.1](https://www.ncbi.nlm.nih.gov/protein/739073916?report=genbank&log$=prottop&blast_rank=1&RID=6M9U8114014) |
| --- | --- |

>Bc-ScaH3_ [WP_050753563.1](https://www.ncbi.nlm.nih.gov/protein/915010015?report=genbank&log$=prottop&blast_rank=1&RID=6MA91417014)

>Bc-ScaI_ [WP_036937898.1](https://www.ncbi.nlm.nih.gov/protein/739066490?report=genbank&log$=prottop&blast_rank=1&RID=6M7GBDBM014)

>Bc-ScaJ_ [WP_050753121.1](https://www.ncbi.nlm.nih.gov/protein/915007477?report=genbank&log$=prottop&blast_rank=1&RID=6MAK0DD9014)

>Bc-ScaL1_ [WP_036947391.1](https://www.ncbi.nlm.nih.gov/protein/739076195?report=genbank&log$=prottop&blast_rank=1&RID=6M9ZZ4F7014)

| >Bc-ScaL2_ | [WP_050753370.1](https://www.ncbi.nlm.nih.gov/protein/915008949?report=genbank&log$=prottop&blast_rank=1&RID=6M73Z9MM014) |
| --- | --- |

>Bc-ScaM1_[WP_050753749.1](https://www.ncbi.nlm.nih.gov/protein/915011090?report=genbank&log$=prottop&blast_rank=1&RID=6HWWB4P6014)

>Bc-ScaM2_ [WP_050753639.1](https://www.ncbi.nlm.nih.gov/protein/915010452?report=genbank&log$=prottop&blast_rank=1&RID=6M7CB1JC015)

| >Bc-ScaO_ | [WP_036945658.1](https://www.ncbi.nlm.nih.gov/protein/739074374?report=genbank&log$=prottop&blast_rank=1&RID=6MA3428A014) |
| --- | --- |

>Bc-ScaP_ [WP_050753797.1](https://www.ncbi.nlm.nih.gov/protein/915011358?report=genbank&log$=prottop&blast_rank=1&RID=6M987RT7014)

>Bc-ScaQ_ [WP_036944738.1](https://www.ncbi.nlm.nih.gov/protein/739073423?report=genbank&log$=prottop&blast_rank=1&RID=6M9AEZWF014)

>Bc-ScaR1_ [WP_036945445.1](https://www.ncbi.nlm.nih.gov/protein/739074147?report=genbank&log$=prottop&blast_rank=1&RID=6M9FZEVF014)

>Bc-ScaR2_ [WP_036941772.1](https://www.ncbi.nlm.nih.gov/protein/739070405?report=genbank&log$=prottop&blast_rank=1&RID=6M9P7T97014)

>Bc-ScaR3_ [WP_050753288.1](https://www.ncbi.nlm.nih.gov/protein/915008466?report=genbank&log$=prottop&blast_rank=1&RID=6M9S6T3Y014)

>Bc-ScaS_ [WP_036937916.1](https://www.ncbi.nlm.nih.gov/protein/739066508?report=genbank&log$=prottop&blast_rank=1&RID=6M8RA5R1014)

>Bc-ScaT_ [WP_036945212.1](https://www.ncbi.nlm.nih.gov/protein/739073908?report=genbank&log$=prottop&blast_rank=1&RID=6M8WDNG1014)

>Bc-ScaU_ [WP_036942961.1](https://www.ncbi.nlm.nih.gov/protein/739071621?report=genbank&log$=prottop&blast_rank=1&RID=6M9J5MZW015)

>Bc-ScaV_ [WP_036941393.1](https://www.ncbi.nlm.nih.gov/protein/739070020?report=genbank&log$=prottop&blast_rank=1&RID=6M9XWDY7014)

>BC-ScaW1_ [WP_036940412.1](https://www.ncbi.nlm.nih.gov/protein/739069023?report=genbank&log$=prottop&blast_rank=1&RID=6MAN484M014)

>BC-ScaW2_ [WP_050753371.1](https://www.ncbi.nlm.nih.gov/protein/915008955?report=genbank&log$=prottop&blast_rank=1&RID=6MARDMU1014)

>Bc-ScaX1_ [WP_050753468.1](https://www.ncbi.nlm.nih.gov/protein/915009484?report=genbank&log$=prottop&blast_rank=1&RID=6M9KRTUG014)

| >Bc-ScaX2_ | [WP_036944734.1](https://www.ncbi.nlm.nih.gov/protein/739073419?report=genbank&log$=prottop&blast_rank=1&RID=6M9DSERH014) |
| --- | --- |

>Ac-ScaA1_ [WP_010247249.1](https://www.ncbi.nlm.nih.gov/protein/497933093?report=genbank&log$=prottop&blast_rank=2&RID=72307BMC015)

>Ac-ScaB1_ [3BWZ_A](https://www.ncbi.nlm.nih.gov/protein/220702354?report=genbank&log$=prottop&blast_rank=1&RID=7231TUVY014)

>Ac-ScaE2_ [WP_010250346.1](https://www.ncbi.nlm.nih.gov/protein/497936190?report=genbank&log$=prottop&blast_rank=1&RID=7239JP0G015)

>Ac-ScaH_ [WP_010245015.1](https://www.ncbi.nlm.nih.gov/protein/497930859?report=genbank&log$=prottop&blast_rank=1&RID=723EF363015)

>Ct-CipA3_ [AGK30455.1](https://www.ncbi.nlm.nih.gov/protein/482691278?report=genbank&log$=prottop&blast_rank=1&RID=722SYGRY015)

| >Ct-Orf2p1_WP_003519375.1  >Rf-FD1-ScaA1_ [WP_009986657.1](https://www.ncbi.nlm.nih.gov/protein/497672473?report=genbank&log$=prottop&blast_rank=1&RID=723R2U2P014) |
| --- |
| >Rc-ScaA1_ [WP_054685087.1](https://www.ncbi.nlm.nih.gov/protein/938901156?report=genbank&log$=prottop&blast_rank=1&RID=723GB4KY014)  >Rc-ScaB7_ [WP_054685074.1](https://www.ncbi.nlm.nih.gov/protein/938901143?report=genbank&log$=prottop&blast_rank=1&RID=723MTVFR014) |
