# Supplementary material for: Unique organization and unprecedented diversity of the Bacteroides (Pseudobacteroides) cellulosolvens cellulosome system
Source: Biotechnol Biofuels. 2017 Sep 7;10:211. doi: 10.1186/s13068-017-0898-6 (PMC5590126; doi:10.1186/s13068-017-0898-6)
Supplement: Supplementary file 4 — Additional file 4: Figure S3. Multiple sequence alignment of the five Bacteroides cellulosolvens type I and miscellaneous dockerin modules. The alignment shows two internal dockerin repeats of B. cellulosolvens type I and miscellaneous dockerins that contain unique sequences. The left part of the sequence represents duplicated sequence 1, and the right sequence part represents duplicated sequence 2. Cyan highlight indicates putative calcium-binding residues. Yellow highlight indicates putative recognition residues. A: Alignment length: 71. Identity (*): 30 residues = 42.3 %. Strongly similar (:): 11 residues = 15.5 %. Weakly similar (.): 9 residues = 12.7 %. C: Alignment length: 65. Identity (*): 28 residues = 43.1 %. Strongly similar (:): 17 residues = 26.2 %. Weakly similar (.): 7 residues = 10.8 %. D: Alignment length: 78. Identity (*): 7 identical residue = 9 %. Strongly similar (:): 13 residues = 16.7 %. Weakly similar (.): 5 residues = 6.4 %. E: Fragmented dockerins: Two ORFs that resemble a dockerin sequence were found in the genome. One of the two ORFs was not annotated and presented with its ordinal number – ORF1413. [file 13068_2017_898_MOESM4_ESM.docx]

**Additional File 4:**

**Figure S3. Multiple sequence alignment of the 5 *Bacteroides cellulosolvens* type I and miscellaneous dockerin modules.** The alignment shows two internal dockerin repeats of *B. cellulosolvens* type I and miscellaneous dockerins that contain unique sequences. The left part of the sequence represents duplicated sequence 1, and the right sequence part represents duplicated sequence 2. Cyan highlight indicates putative calcium-binding residues. Yellow highlight indicates putative recognition residues.

**A**: Alignment length: 71. Identity (*): 30 residues = 42.3 %. Strongly similar (:): 11 residues = 15.5 %. Weakly similar (.): 9 residues = 12.7 %.

**C**: Alignment length: 65. Identity (*): 28 residues = 43.1 %. Strongly similar (:): 17 residues = 26.2 %. Weakly similar (.): 7 residues = 10.8 %.

**D**: Alignment length: 78. Identity (*): 7 identical residue = 9 %. Strongly similar (:): 13 residues = 16.7 %. Weakly similar (.): 5 residues = 6.4 %.

**E**: Fragmented dockerins: Two ORFs that resemble a dockerin sequence were found in the genome. One of the two ORFs was not annotated and presented with its ordinal number – ORF1413.

**A**: Type I Dockerins


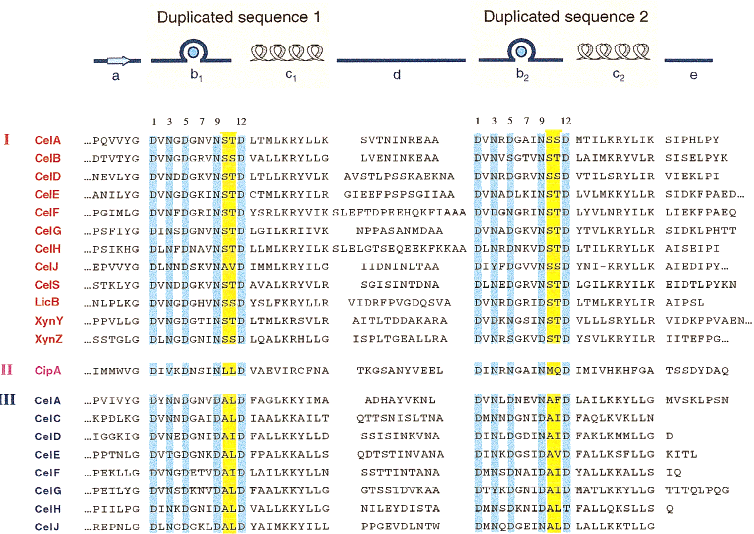


ScaL2 VLWGDVDGDGNFNSNDYALMRQCLLGMISKES----VAATADVDGDGKLTSDDYAYMKRHLLGMISVFPVE

ScaV TVYGDFDNDGNFDSDDYALFRQFMLNMIDKE----IVPATCDVDGNGLYNSDDYAYMRQHLLGMIKIFPVQ

ScaA2 FVIGDVDCSGSVNSDDYAYIRQYLLGMIENFPCVVNGLKAADVDGDGNIDSDDYAYMRRWLVGMIDKFPAE

ScaL1 VVYGDFDQDGNVDSDDYAYMRQYLLGMISDDK----IPKTADVDGDGNYDSDDYAYMRQYLLGMISVFPAQ

ScaA1 VLYGDVDNDGNVDSDDYAYMRQWLIGMIADFPGGDIGLANADVDGDGNVDSDDYAYMRQWLIGMISEFPAE

: **.* .*..:*:*** :** ::.** . .****:* *******:: *:***. **.:

**B**: ScaP Dockerin

ScaP FIYGDVNGDSTVSMNDTETFRSYMLGSNSGFTYQYGQLAADVDGNGLVNGLDMGHILMFAQGKISKFPVQ

**C**: ScaR Group Dockerins


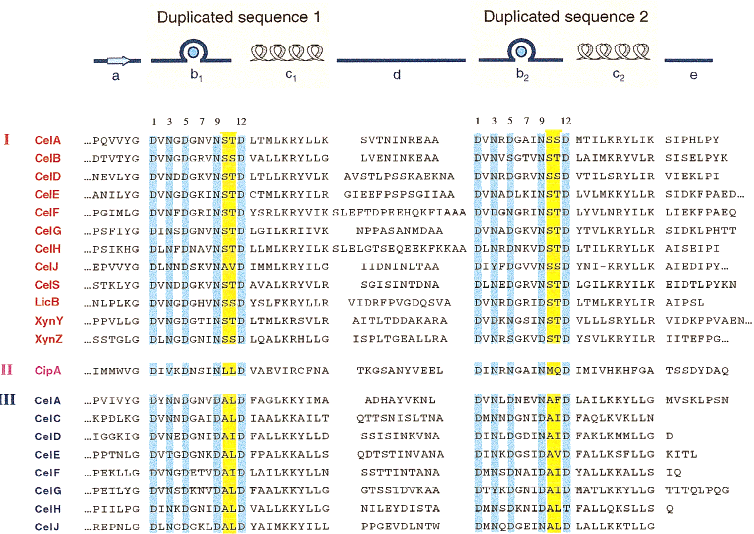


ScaR1 IRIGDVNKDNKVTYVDGLKTLQYITGKTTLDDQALIAADIIGMDGVNVNDVVTILKADVGSITLE

ScaR2 IKTGDVNRDNKVNYIDGLKTLQHITGKTKLDPQAMIAADVLDMDGVNINDAVTMLKADIGLITIE

ScaR3 ILIGDVNKDRKVNFIDALLILRSITGKIIFDQQQLISANVDGTEGVTVNDAVLVLKADIDLVSLN

* ****:*.**.::*.* *: **** :* * :*:*:: . :**.:**.* :****:. ::::

**D**: Miscellaneous Dockerins


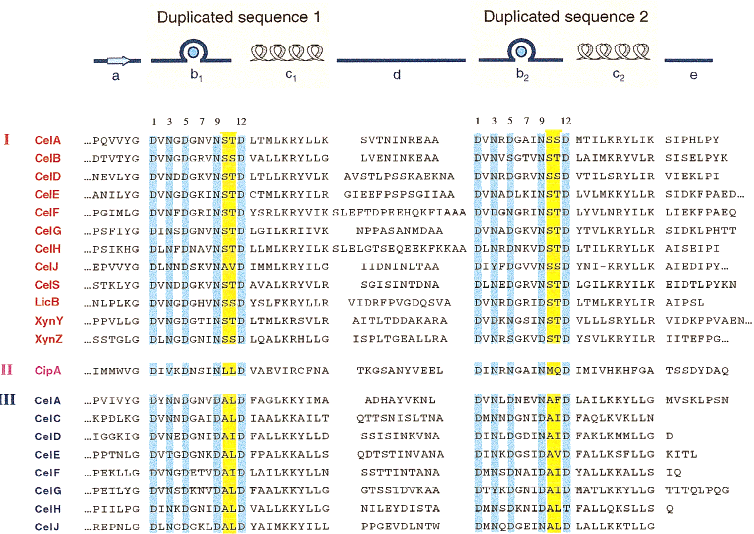


WP_036945201.1 IMVGDVNGDSYVNSGDSTAILSIISNSYNTTNISTKYLTLSADINRDGTIDIRDVNTLNQYINGSSSVKLYYVR----

WP_036940956.1 VTIGDVNLDGSVNAIDLLLLKQEIDCLVYFD----NLQCIAADVDYNAAITMADYNMLKDYLLGNITSFPAGSSY---

WP_036945116.1 VKIGDLNKDGYINNTDHNMLSKHLLGSTPLTY---AQQKVAADVNGDGFINALDNGLFQKYLAGTITVFPAGSNFTY-

WP_050753467.1 WKFGDVTGDGLVNAADYSNLRSYLLKKVVSLPAT--AWLFSADVSGDNLINAGDYSILQSYLLKKTTQYPTMQAENKY

WP_036945214.1 YVPGELNGDGEINSADRELLKKFIFGIEKTFPLAVGYESRLADLNGDGKVNSIDYAAFKKFLLQYITVFPNKNY----

WP_036943752.1 LRVGDVNGDGFINSNDFELLRSHLQNSKIKFPVD--DPIWVADLDGNGYINSIDLAYMNQYLQGKRTFFPKEMSL---

*::. *. :* * : . : **:. : : * ::.:: :

**E**: Fragmented (Incomplete) Dockerins

WP_036938446.1 AVYGDFDKDNDFDSDDYALLRQFLLGMIAFELAPVTADVDGNSRLDSDDYAYMKQHLLGMINKF---

ORF1413 -------------MADVIEIAKGFNSILGETNHSTNYDLNQDKSINMSDIIIIARHFGAIASNHPGL
