# Supplementary material for: Unique organization and unprecedented diversity of the Bacteroides (Pseudobacteroides) cellulosolvens cellulosome system
Source: Biotechnol Biofuels. 2017 Sep 7;10:211. doi: 10.1186/s13068-017-0898-6 (PMC5590126; doi:10.1186/s13068-017-0898-6)
Supplement: Supplementary file 5 — Additional file 5: Figure S4. Multiple sequence alignment of the 146 Bacteroides cellulosolvens type II dockerin modules. The alignment shows two internal dockerin repeats of B. cellulosolvens and was used to create Figure 6 representing the Weblogo of the dockerin repeats. The left part of the sequence (before the hyphen) represents duplicated sequence 1 and the right part (after the hyphen) represents duplicated sequence 2. Cyan highlight indicates putative calcium-binding residues. Yellow highlight indicates putative recognition residues. Alignment length: 65. Identity (*): 3 residues = 4.6 %. Strongly similar (:): 3 residues = 4.6 %. Weakly similar (.): 4 residues = 6.2 %. [file 13068_2017_898_MOESM5_ESM.pdf]

Additional File 5:

Figure S4. Multiple sequence alignment of the 146 *Bacteroides cellulosolvens* type II dockerin modules. The alignment shows two internal dockerin repeats of *B. cellulosolvens* and was used to create Figure 6 representing the Weblogo of the dockerin repeats. The left part of the sequence (before the hyphen) represents duplicated sequence 1 and the right part (after the hyphen) represents duplicated sequence 2. Cyan highlight indicates putative calcium-binding residues. Yellow highlight indicates putative recognition residues. Alignment length: 65. Identity (*): 3 residues = 4.6 %. Strongly similar (:): 3 residues = 4.6 %. Weakly similar (.): 4 residues = 6.2 %.

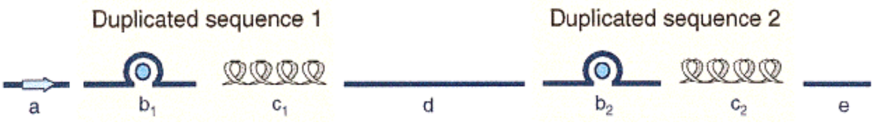


```
KNY28160.1       IWAGDLQQDGSINMADVLLLALSFNSIKTDK-SFVEHYDLNKDGIINMSDLIIMAAHFNKTPADY
WP_050753172.1   LWAGDSNSDGSINMSDVIQLAKIFGSVSGDG-NYKADVDFNKDKNINMLDVIIIAKRFNATSMDY
KNY30445.1       KISYDLTDDGYINMADVIVLATKFNSVKGDL-AYTEMCDFDKNGAINMADVIILARLFNSAV---
WP_050753933.1   LMAGDFNGDNAINMSDVIILAKSFNSISNDS-KYAAGIDLNRDGSINMSDVLILAMNFNKTSLNY
WP_050753725.1   ASIEDINNDGVINMADVILMAGAFNTVRGDS-IYNSSCDLNNDGTVNMSDIVIIATKFNVIVRH-
KNY27843.1       TNRIDINNDGVINMADVILLANVFNSVHGDS-KYVEAYDINKDGAINMSDVILIAAKFNAIV---
KNY27845.1       ANSSDINHDGVINMADVILLASRFNTVIGNA-GYVDAYDLNKDGAINMTDVILIAAKFNTIV---
KNY27838.1       KNSLDVNLDGAINMGDVIMLAIVFNSIRGDA-RYVETCDLNKDGVVNMSDVIMIAAKFNTVV---
WP_050753175.1   VNSLDVNHDGAINMADVIILAKVFNSVSGDS-KYVYAYDLNRDGAINISDIVLIASKFNTVV---
WP_036940830.1   VNNLDINHDGAINMADVIILAKVFNSVRGDS-KYVDAYDLNKDGAINMSDVIMLASKFNTVV---
WP_050753005.1   ILYGDLNEDLSVNMADVIILAKTFNCVKGDR-KYIEANDLNTDGSINMADVVIIAKVFNTVKHME
KNY25662.1       VLIGDIDGNGAVNMSDVVLMAAVFNSVIGDG-DYKASCDLNNDGSINMADAVIIALNFNKTIND-
WP_036941945.1   PVPQDVNGDRVVNMADVILIASRFNAIREQS-KYDVKCDLNSDGVINMSDVIMIASKFNYTY---
KNY27826.1       NLTGDINGDGIVNMSDVILLALSFNSVNGDI-NYKAEYDLNSDGVINMKEVIIIAVNFNAVLK--
KNY30347.1_      SAPEDINGDKVVNMSDVILMASHFNAVSTDS-NYDRKCDVNGDGAINMSDVIMVAAKFNKSV---
WP_050753500.1   TPPGDLSNDGIINMSDVILLAGVFSSYRGDS-KYVELYDLSNDGVINMSDVIILAGNFNKTATSA
KNY26478.1       SISYDINKDGVINMSDVILIAMKFNSVRGDD-KYTDLCDLNRDGAINMADVILIAGKFNKTA---
KNY25998.1       VISADINNDGVVNMADVILLATKFNSVKGDS-KFVDNYDLTRDGSINMADVLMIAVKFSKS----
WP_036946597.1   MKSPDLNGDKVVNMSDVILIATVFGAVSGDG-KYKSSYDINGDGAINMSDVIIVATSFNKTVNDP
WP_036942003.1   VLKEDLNKDGVINMTDVILLATSFNMVREDP-KYVEARDLNGDGAINIADVIILAAKFNTVI---
WP_036938130.1   SKGPDLNGDGAINMADVILIAMVFNTTSENS-KYIPAYDLNGDGAINMADVIAIASNFNKLVQST
KNY27825.1       TTKGDLNNDGVINMSDVILLASTFNAVRGDF-KYALAYDLNDDGVINMSDVIVIAGNFGEIVAIA
WP_050753182.1   DIKGDINNDSVINMADVILLAGAFNAVSGDP-RYAPIYDINNDGVINMADVIIIAANFGKTVPKA
WP_036943551.1   AGSGDLNNDGIVNIADVVLLGVIFNSIAGDN-KYRPSCDLNSDGVINMADVIIIAGNFNKAVTV-
KNY27842.1       SSSPDLNNDGVINMADVIILAGGFNSVRGES-KYVAAYDLNNDGAINISDVIIVAGNFNKIITPV
KNY25189.1       TILGDINNDGVINMADVLLLAIKFNSTAGDG-KYLASYDLNNDNTINMADILVIAINFGAIVPVA
WP_050753003.1   AIRGDLSNDGVINMADVILLATKFNAIDGDG-KYVSAYDLTDDGVINMADVVIIGKYFGKNVMVV
KNY27799.1       TKTPDLNGDKVVNMADVILLAGAFNAVSGDG-KYKAEYDLNVDNAINMADVIIIAGQFNTII---
WP_050753051.1   TKTADLNNDGIINMADVILLATTFNSVAGDG-KYIGSYDLNSDGAINMTDVITLATQFNKPPVII
WP_036946577.1   ASTGDLNNDGVINMSDVISLAQTFNSVIGDG-KYVASYDLNSDGAINMSDVIVIASKFNTIITNP
WP_050753313.1   SKTPDLNGDRVINMADVILMAQTFNSVAGDG-KYKAAYDLNSDGAINMSDVIAIASNFNKAIG--
KNY27806.1       VTTGDLNNDSVINMADVILLATKFNAVSGDG-KYVAAYDLNSDGVINMADVIIIASKFNTVVS--
KNY26370.1       SLKGDLNNDGVINMADVILLATKFNSVAGDG-KYVASYDLNSDGSINMADVIIIASNFNKTVSTT
WP_081926923     SLKGDLNNDGVINMADVILLATKFNSVAGDG-KYVASYDLNSDGSINMADVIIIASNFNKTV---
KNY28540.1       EKTPDLNSDGVVNMADVILIATTFNSITGDS-KFKAAYDLTNDGVINMADVLIIGKMFNKSI---
KNY27822.1       TIKGDINNDGIVNMADVLLLASAFNAANGSA-KYNLAYDLNGDGVINMADVLIIASNFNKSGSIV
KNY25430.1       AVKGDLNNDGVINMADVILIATAFNATSGSE-KYKAAYDLNNDGAINMADVLTIAQNFNKSVTQV
KNY28459.1       SLKGDLNDDGIINMADVLLVATSFNATPSFP-KYNAAFDLNEDGAINMADVLIIAQNFNKPVT--
WP_050753002.1   VVSEDLNNDGIVNMADIILIATTFNSVTGNE-KYNKAYDLSNDGSINMLDVIIMAKKFNTVRN--
WP_050753934.1   SVTEDLNNDGTINIADIILIAITFNSVVGNP-NYNKAYDLSSDGAINMLDVIIIAKKFNMVMHE-
WP_036935052.1   SKIADVNADKVVNMSDVIIMASVFNSSKGDG-VYIESYDLNSDGAINMNDIIVIAKNFNTIISD-
WP_050754083.1   HSSGDLNGDGKINMADIIILATSFNAVRGEG-KYIEANDINRDGVINMADVIIIAAKFNSTV---
KNY29902.1       YVSVDLNGDRIINMADLIILASTFNAVRGDG-KYAEANDLNKDGAINMSDVIVIAAKFNSSI---
WP_036946550.1   IPTGDFNNDGIINMADVINLARRFNKVKGDQ-TYNLAYDLKNDGVINMADVLIMASKFNKTI---
KNY26476.1       TKSLDLNSDGVINMSDVIIMALTFNAIRGDG-KYKDAYDTNSDGAINMADVIKIAEKFNTIISAT
WP_036945378.1   QNSPDLNKDGVVNMTDVILLAKVFNSVHGDN-QYIASYDLNNNGSINMSDVIIIAKAFNTVIT--
KNY27855.1       ASIGDCNKDGVINMADVIELACRFNKTSGDM-MYNLVYDLNNDGVINMADVIIIACKFNTIC---
KNY27824.1       IPTGDLNKDGIINMADVILLAKAFGSVIGDS-KYDPSYDLNNDGAINMSDVIIIASKFNTVISSP
WP_036942018.1   VATGDLNKDGVINMADVIILAVAFNSIHGDI-KYNKLYDLNNDGAINMADVIIIASKFNT-----
WP_036940084.1   MWPGDVLQDDAINFVDVLLMAKSYNAIKNEV-LYSKICDFNLDSSVNVADVIIMSRHFNAVPNEY
WP_036936449.1   MLPGDLCKDGAINLKDVLELAKSFSAVVGDA-NYSEIKDFNRDNAINMADVIIIIKNFNETSDSE
KNY28903.1       IWAGDTTQDGAINMADVLQIAKFFNSTSGST-KYNAEADLSNDGVVNMADVLIVAKHFNATIDSY
WP_036944080.1   LWAGDITHDGAINLSDFIEIAKSFNAVSGDK-NYKNGLDLNIDGAINMLDVLIIAKHFNQTINDY
```

WP_050753404.1	IWPGDIDASGVINMSDIILMAGSFNAVKEDS-KYNESCDFNLNGAINMEDVLFVAKRFGNTSENY
WP_050753852.1	MWAGDVLQDNAINMSDVIKIANSFNAITTDA-NYSTAADININGSINMEDVIILAKGFNSTSNDY
WP_050753851.1	LLAGDITQDGTINMSDAIKIAGSFNAVKNDV-KYTESTDINSNGSINMEDVIILATAFGKTSSSY
WP_036936247.1	MYGGEIIKDGIINLADIMEFVKRFAKQVDNE-SADKACDLDNDGCINLKDIMIAANNFNKSTDD-
WP_050753358.1	IWGGDINKDKSINMLDLILIAKLFNVNSTDGGKYIYNRDLNADNVINMEDVIIVAQHFNLTTVDY
WP_050753919.1	ILPGDIDGDSSINIADCVYLAKMFNSIKGDN-IYSADADINKDGAINLSDFVYIAKYFNMTSAAY
WP_036945613.1	MFAGDIDQNGLINIADILETAEGFNTTSANE-NYKPKCDINGDDCINIIDIMIVAKNFGKSSESY
WP_036943605.1	IWPGDIDQDGTINMKDVIMIAKLYNVSSKES-GYNAGYDLNQDGVINISDIIAIAKHFNASTSSY
WP_036945212.1	MWAGDINGDGAINMADIVEVSKVFNTSSDDI-GYKAKCDMNKDGRVNMADIVAIAKHFGCVSVDY
WP_036945397.1	LLAGDNNGDGSINFADVVEIAKVFNTNSNNE-NYKADCDINKDNSINIADVIIIAKHFNETSG--
WP_036946480.1	LWAGDINQDNSINMADVIKIAQCFNSNSDDE-NFKPDYDINKDKTINIADIIIVAKHFNATTDSY
WP_036935657.1	IHWGDLNNDEIVNIADIIVVSSNFGRTSSST-SYNKDADLNSDGVINMNDIMKLSNIFSATPENQ
WP_036941407.1	IIAGDIDQNDSINMADIAEIAKAYNSVKDSS-LYRIDADFNADGVINIKDIMIAAKRFNKVSEDF
WP_050753935.1	FKQVDLNHDDIINMDDIIIMAKAFNTAKGHE-SYNAACDFNEDGCINLLDIAVISKMFGQNKD--
WP_036935113.1	MLIGDIDNNGVINIADIIQMTNSFNSVDGDS-WYRYAYDLNRDNVINMADIIMIAKNFNMASTDY
WP_036944928.1	MWAGDINGDKRINIADIILMSSSFNMSYTAS-DQNIVSDFNKDKSVNMADVMIIARHFGATVNDY
WP_036945094.1	SLTGDINQDNIVNMADVIMMAKEFNGNKPG-----SPCDINGDSIINMSDIIIVAKNFNKSAIA-
WP_050753224.1	MWAGDVNGDKISNMADLVVIANFFNTLRGAG-NYSDKVDLNDDGAVNMSDVVIIAKNFNKTSGDY
WP_036946417.1	MWAGDINQDNSVNMSDIVAIAACFSTSKGES-KYKGSIDINGDGAVNMSDVVIIARNFNRTSNDY
WP_036937898.1	MLCGDINNDNSVNMSDVIQMAKTFNLSKGTE-GFIDTADFNRDSVVNMNDIVLAAKNFSKSASDY
WP_050753260.1	IWPGDFNGDLIINMSDIVQISKVFNTAALES-TYNQIYDVNNDNAINMLDVVGVAKNFNKKSSDY
KNY27959.1	IWAGDFNGDLSINMADIIEFAKGFNLISSSE-KYNLVLDMNQDGAINMSDIMIVIKHFSKTANDY
WP_050753159.1	LWPGDFNSDNSINMSDIILMAKVFNAVKGDV-KYNADFDLNKDDVVNMTDLVLIAANFNKVSSEY
WP_036945534.1	LFAGDINQDNTINMNDIIELAKCFGSSNEND-LYNEVCDFNKDQVVNFSDVIIVAKNFNKTSSDY
KNY28878.1	MMAGDINGDNAINMTDIIQIAKAFNASKGGS-GYNEAYDLNMDGSVNMTDIIVIAKYFGKTSSDY
WP_050753784.1	MFAGDINGDSSINMSDIIKLALSFNALKGSP-GYNGVCDFNLDGNINMSDIIIMAVNFNKTPADY
WP_050753119.1	LFAGDLNGDNSINMSDILLLAKSFNATKESS-NYDDINDLNGDGSINMNDVMIVAKNFNKTSADY
KNY28320.1	IVKCDINSDGVINIADIILIARYFNSTYGDG-RYETMFDLNCDGFINMIDILRVAVYFNQKL---
KNY26018.1	KSKVDVNGDGVINMADVIIIAKKFNAVSTDS-NYDVNCDLNVDGAINIGDVIMVAAKFNTQA---
KNY30340.1	LHSEDLNGDNVVNMADVILIGLYFNQLVN---EQNKRFDVNKDGVINMSDVMKIAISFNKFTISN
WP_050753071.1	PAPEDINGDMVVNVKDVILAATHFNTISSGN-NYDIKCDLNDDGYINMIDIMMIVEKFNHTY---
KNY30341.1	TLVEDINGDRVVNMADVIMLARNFNAQVT---DSTRSCDLNSDGVINMSDVIKVALKFNTSM---
KNY25261.1	LNAEDINGDKVVNMADVIIIAGFFNQLSS---QSNIKCDLNSDGVINMSDIIILAKKFNKIF---
KNY27551.1	LDTEDINGDKVINMADIILIAYHFNSTASGT-NYDIKCDLNNDGAINMSDVILIALKFNKIISVA
KNY29222.1	ENPEDINGDYVINMSDIILMAAHFNATLYSG-NYDKKYDINNDGVINMGDIIIVAKKFNKIIEKG
WP_036944865.1	IFLADVNGNGIVNMTDVIVVAVSFNSSIGDP-LYSERLDLDKSGTINMGDLIIVAKYFNQNVPIP
WP_036940816.1	ISSVDFNDDGAVNMLDVIVLASAFNSKSGDT-RYLVKIDINHDGVINMSDVIYIAKYFGRIIEKP
KNY25250.1	LWVGDIKPDNMINMEDVVKIAVEFNTNFKEA-GFKYEYDLNKDNVINIMDFAAIARNFNTNSSDY
WP_050753644.1	IWCGDANQDGVINLIDIIKLALLFNSTSGDY-KYDESIDFNMDGVINVRDFYVVSRHFNQTKNDY
WP_050753782.1	ICPGDIVQDNAINMMDVIEIASLFNTFEGSE-NYR--CDLDRNGVINIEDFLIIAKHFNKCSEDY
WP_036945773.1	LWGGDVNQDDMINMLDIVELAKVFNTAATDE-NFKYNCDINGDNITNMLDITIMAKHFNQNSNDY
WP_050753775.1	LWGGDVNGDNSINMADIVNIVKEFNTTLTST-IFKHDYDFNKDNTINTYDVVIMARHFNQTTEDY
WP_036946329.1	MWPGDIVKDNAINMVDVIDLAKAFNSKEGDE-LYNTACDINMDKVINIADIVIIAKYFGKTINDY
WP_050753406.1	MWGGDILQDNALNISDVIEIAKAFNSVKGDQ-IYNPASDVNMDNSINIMDMVIIAKHFNATPDSY
WP_036937631.1	MWCGDVVNDETINMTDIIKLAKLFNTAFGDG-IYVDSCDFNKDKAINISDVMIIAKHFNQTSEDY
KNY26737.1	MWIGDTNGDGAINMSDIMAYALVFNSTSGDG-KYKQDLDVNQDGAINMSDIINVAKHFSASVISY
WP_050753580.1	MYPGDINADDTINMGDIVAIAKVFNTTINDA-AFNASADLNNDKTINMSDVIRVSKYFNINSDKY
WP_036944515.1	LYAGDVNGDDTINMNDIVMMAKAFNSTLNGI-QFNDAADINSDKTVNMTDVVIVAKNFNKSSGDY
KNY30355.1	MWAGDLNQDGTINMSDIIIMAKYFGGVKGDG-TYLPDADLNSDSSINMSDVMILAKHFNATTSSY
WP_050753120.1	IWGGDSNQDGTINMSDIIVVAKAFNSTSEAA-NYNKDADINLDGTVNMSDVVIAAMRFNKTTADY
WP_036945157.1	MWGGDFYRDNVINMQDLIILAKVLNVDSSDE-KYSYVYDLNRDKVIDMKDVFIIARHFCDAREDY
KNY25239.1	LMEGDMNNNQVINMEDVIIIAKVFSLTSSHT-LYNAAADFNNDGIINMSDIIMLAKNFGKTYLDY
WP_050753884.1	LWPGDINQDNAINMSDVMGIAQRFNVTYQDS-KYDLLCDLDRNNAINMSDVIIIAGHFNKSISGY
WP_036945658.1	IWAGDLNEDASINMYDIVMLAKSFNKINTDF-GFNDAGDLNKDNAINMNDVIVMAKHFNSISSTY
WP_050753081.1	LWAGDINSDNTINMADVIEMAKSFNATSGEV-KFIANCDINKDNTVNMADIVIIAKNFPRIQGVI
WP_036935236.1	MLSGDANNDNAINMSDAILIAKLFNKTTADK-DFNPAADFNTDAVINVADVVCVAKNFNTTGDSY
WP_036935066.1	MIPGDINTDSVINMSDIVLLAKSFNTIPSDV-GFNSAADFNYDKVINMGDVVIIAKNFNATSADY
WP_036943587.1	MWAGDVNQDNIINMSDVIGIAKSFGVTSKDP-GFNIAADFNCDKIINMSDVIIICKHFNKASEDY
WP_036943585.1	MWAGDINQDNAINMADVIEMAKSFGMTSEDT-SFNAVGDFNCDNVINMTDIVTLSKHFNKISSDY
WP_036938565.1	MVPEDFNQDGVVNMADVVLLAKEFGNI--II-RYNPKYDLNNDGAINMADVIKVAIKFGYIYKLE
WP_036939882.1	YLKEDINKDFTINMADVVMIAKAFGFAKANT-NFDSRCDLNGDNSVNMSDVIKLALKFGYSY---
WP_050753430.1	YIIEDINKDGCVNMVDVVLLAKVFALTKVDT-GYDIKCDLNNDGTINMSDIVKLAQKFGYIYKL-
WP_050753431.1	EIMEDINKDGSVNMADVVLVAKVFGFMKDDP-EFDKKCDLNDDGTINISDIVKLALKFGYTYTL-
WP_050753432.1	KVTEDINKDGSVNMADVVLVAKVFGLTKDDL-EFDKKCDLNNDSTINISDIVKLALKFGYTYTS-
WP_050753593.1	VVLEDINKDGTVNISDVVLVAKAFGTTYDSI-NYDPLADVNKDGVVNMVDAIAIAGKFGYTYSHK
WP_036938562.1	VVVEDINKDGVVNISDIVIIAIAFGTTPDCK-DYNALADLNQDGAINMVDVVAIARKFGYAYSLK
WP_050753843.1	PNPADINGDGKVNMRDVVEMALAFGTTSKDP-NFNKKCDLTNDGAVNMSDVMALAMRFGDEYELP
KNY27848.1	VKNVDVDHNGVVNMADVILIASAFNTVQGDV-KFVESYDFNSDGAVNMADIVMIAAKFGNIL---
WP_036938559.1	RIPEDLNNDGVINMADVVIIAFAFNSTW----ETDLTCDLNNDGAVNMTDVMRLALKFGVVYRK-
WP_050752989.1	LVAEDINGDSTVNMTDIMEIAKRFGAVAGDA-RYKINCDLNSDGAINMTDVIMIAKKFGFTY---
WP_036942563.1	SVKEDINKDKAVNMMDVAMMAKAFGGVAGDT-LYDSKCDLDGDGSINMNDVVILAKKFGFTY---
WP_036945506.1	GWVEDVNQDGTVNMSDVIMLAKSFGKVSGES-GFDPKCDLNKDNSVNMADVVLIGIKFGKTY---
WP_036936730.1	KVPEDVNRDGVVNMVDVMRIATAFGTVSGNV-KFEPACDLNSDGAVNMADVMKLAVKFGYTYSL-
KNY27224.1	YMPEDVNRDKAVNIADVVEIAKRFGKVNGDP-GYDTRYDLTNDNSINMADVIKIGLKFGQTY---
WP_050753145.1	GVVEDINNDGTVNMHDVVIIAKAFGTVPGDK-IFDKRCDLTNDNGINMADVMILALKFGFVYSV-
WP_050753594.1	GIAEDINKDRVVNMIDVFIIAKAFGFCLGDD-KFDICCDLNNDGAINMADVMRLSLKFGCVYELE
WP_050753192.1	LIPGDLNNDEAINIGDAILIAASSFSKLAV---VNYEMDLNNDGVINMSDIIIIAQNFGKTSADY
WP_036944039.1	GLKGDVNGDGVVNMADAIAIAGVFGKEAI----AKPSADLNIDGVINMSDVIIVAQNFGKTK---
WP_050753099.1	KLKGDLNGDGVINMADVMILAQSFGKAIGNP-GVNEKADLNNDGVINMADAIILAQYFGKTKSAE

```
WP_050753563.1    PITGDLNGDNAVNMTDVVEVSKYFNTLNTDP-NYKKEYDINLDNAINMIEIMIIASKFNTISNSI
WP_050753282.1    LPDGDINGDRIVNMADVIILAGCFGLTSGET-GFLIECDINRDGAINMKDVIIIANNFGKTNR--
WP_050753050.1    ANVEDVNGDGVVNMADALIIATAFNSTTGEP-KFKANCDLDRNGVINMADILLIAAKFGKTF---
KNY29876.1        RIAEDINMDGVVNMADVLQIAIAFNSVRGNP-NYKTILDLNNDGVINLSDVIMCAVKFNTICY--
WP_036943545.1    TIVEDVNGDRAVNMADVILLAKCFNSALGDS-KYNENYDINKDNVINFSDIMKIAVRFNYSY---
WP_036945220.1    GVPEDINGDRAVNMADVVLMAASFNAISTSP-NYNKKCDINNDGTVNMSDVVKLSLKFNYTY---
WP_036938093.1    FILEDVNKDRAVNMADVIALASCFGKVRNDS-SYKQECDINKDGSINMSDVMMIAQKFNYTY---
WP_036944617.1    QVVEDVNRDRAVNMADVVQLATCFNATRGTN-KYIANCDINNDGSINMSDVIMIAVKFNYTY---
WP_050753911.1    AVVEDINGDKAVNMADAILIAGCFNATTTSN-KYNTKCDLNNDGVINMTDVIMLAVKFNYTYR--
WP_036944505.1    VYQGDINGDNVINMQDVVQIGKIFNSKNGDK-TYNIQYDPNSDGVINMVDVFVLAKILIW-----
WP_036935054.1    TYIGDVNIDNVINVSDVMMIAGVFNCKYGDS-KYNTIYDLNGDGVVNMMDVIILAKNFSKFGDQD
KNY27620.1        MWAGDMNGDNVINMADVIDVVKGYNTIEGDN-LYIADYDLNKDGVIKMLDIIIIARNFNMTSYMN
KNY25939.1        LWAGDFNLDDSVNMSDVIEVSKAFNSRTGDS-KYNSICDIDKDGSINMSEAIYIATRFNKTSADY
WP_036935603.1    LWVGDFNQDSSINMADVLEISKSFNSTVGDP-LYISDNDLDSDGVINLKEVMLIARRFNMTSFDY
                      :   .   *. *                      *   .   .  :         :
```
