# Supplementary material for: Unique organization and unprecedented diversity of the Bacteroides (Pseudobacteroides) cellulosolvens cellulosome system
Source: Biotechnol Biofuels. 2017 Sep 7;10:211. doi: 10.1186/s13068-017-0898-6 (PMC5590126; doi:10.1186/s13068-017-0898-6)
Supplement: Supplementary file 6 — Additional file 6: Figure S5. Multiple sequence alignment of the 49 miscellaneous Bacteroides cellulosolvens type II dockerin modules. The alignment shows two internal dockerin repeats of the B. cellulosolvens type II dockerins that contain unique sequences (particularly in the first calcium-binding loop) but remain type II dockerins. The left part of the sequence (before the internal hyphens) represents duplicated sequence 1 and the right part (after the internal hyphens) represents duplicated sequence 2. Cyan highlight indicates putative calcium-binding residues. Yellow highlight indicates putative recognition residues. Alignment length: 77. Identity (*): 5 residues = 6.5%. Strongly similar (:): 9 residues = 11.7%. Weakly similar (.): 3 residues = 3.9%. [file 13068_2017_898_MOESM6_ESM.pdf]

Additional File 6:

Figure S5. Multiple sequence alignment of the 49 miscellaneous *Bacteroides cellulosolvens* type II dockerin modules. The alignment shows two internal dockerin repeats of the *B. cellulosolvens* type II dockerins that contain unique sequences (particularly in the first calcium-binding loop) but remain type II dockerins. The left part of the sequence (before the internal hyphens) represents duplicated sequence 1 and the right part (after the internal hyphens) represents duplicated sequence 2. Cyan highlight indicates putative calcium-binding residues. Yellow highlight indicates putative recognition residues. Alignment length: 77. Identity (*): 5 residues = 6.5%. Strongly similar (:): 9 residues = 11.7%. Weakly similar (.): 3 residues = 3.9%.

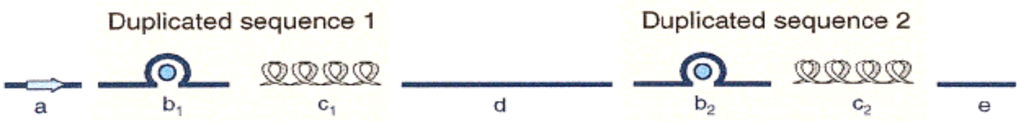


```
WP_050753073.1   ----WAGDSVKYQNGRYEQDSVINMTDIVNLAVSFNT--TSNESGYVAGNDFNNDGVINMADVVIVSKHFNTFY---
KNY28669.1       ----WVGDIKT------PQDGVINMTDIIEVAKVFGS--VTDDGKYDSACDTNLDGAVNMADIVNIAKHFNATTASY
WP_051983551.1   ----LAGDIPNN----GKQDSVINMADIVSIAKCFGT--KNGDVDFSEIFDFNRDNKINMADIMIMAKSFNMTISDY
WP_036941818.1   ----FAGDLNIN----GKQDGSINMADIVKMATRFNT--VSGNEGYSVECDFNMDNSINMADVIIIAKHFNCTSLDY
WP_050753954.1   ----WAGDVTIN----GSHDNVINMNDVIQIARSFNS--------YNANVDFNGDGSVNMSDVIIMAKNFNRTSSDY
WP_050753810.1   ----WAGDILKD----GVQDNTINMVDIIEIARVFGS--VAGDGKYIESYDFNMDNVINLNEVIIIARHFNATSSNY
WP_081927119.1   ----CAGDVGTK----GVQDNVINMSDIIEIAKVFGS--VSGDGKYNSDCDFNMDYSINMSDVIIIARHFNMSSDDY
WP_050753340.1   ----WAGDIAVN----GIKDNTINMTDVIQIARTFSS--VKNDVKYDAGCDLNSDGAINMSDVIIAAKHFNKVSADY
KNY26505.1       ----WAGDVLVN----GNKDNTINMADILEMAKGFNS--VAGDGKYRTDCDLNEDSVINMSDVIIVAGHFNKTSSDY
KNY29562.1       ----LAGDIGEN----SLGDNTINMLDVISAAKAFNL--AKGDMGFSDNCDLNKDDVINIKDIMIIAKNFGRSSTDY
WP_081926754.1   ----WSGDVAKD----GVQDGAVNLSDAITIATAFNS--SSDSPKYKKDCDLNADRSINIADVVIVARNFNKTAQDY
WP_050753716.1   ----YAGDVPKQ----GVQDSMINIIDAMVLASAFNA--TSSDLRYSPFADFNLDESIDMWDVIILAKHFSATTAEY
WP_036942062.1   ----WAGDVPVK----GIQDNSINMIDVVEIAKFFNK--TSSDSGFLAIVDFNKDNAVNIADVIVLAKSFNMTSEDY
WP_081926943.1   ----WAGDLPVK----EVQDGTINIADVVELAKAFNK--VSTDTGFLTCADFNSDNSINMSDVIILAKNFNKTSAGY
WP_050753232.1   ----WAGDIPVN----NVQNNSINMSDVIEVAKSFGK--TSTTSGFISSADFNKDNTINMADVIILAGNFNKTQADY
WP_036946329.1   ----WPGDVSIN----GNQDNVVNMKDVVEIAKRFNS--TLGDSLYSSNCDFNMDKAINIADIVIMAKYFNRTSNDY
WP_036940937.1   ----WAGDVPVN----GQQDNTVNMADVIEIAKKFSS--VSGDSKYSADLDINKDNSINIADIVIIAKNFNKVDTSY
WP_050753025.1   ----WSGDIS------SPNDDKINMSDVISLSKVFNS--VKGDGVYYESADLNGDSVINIMDVVIMAKHFNQTSSDY
WP_050753111.1   ----WAGDID------SSRDGIINMLDVLVLAKTFNT--IKNDPDFMESADINGDFVINILDVFLMAKHFNQTINCY
KNY25653.1       ----WPGDI-------GAQDRAVNIKDVICMASAFNT--SSNQTAYNVDYDINRDNAINISDVIIVAKYFLSIPSDY
WP_036937628.1   ----WTGDVPVS----GVQDNVINTVDQIEISRGFNT--SAGSPDYTLDRDLNKDGNIDMIDISILSRHFNATPGSY
WP_036935069.1   ----WAGDFAVN----SAPDNVINMNDVIGLINSEYFNTYSEIHRYRPICDLNLDNVLNMGDVIILAKYFGKTSEDY
WP_050753132.1   ----WAGDIAIN----GVQNGAINIMDLMQLAKAFNT--ISTDDGYEVDLDINRDGAINMADIFIIIKNFNTSSSSY
WP_036943995.1   ----LAGDISID----GVQDGTINILDAVQMAKCFNS--TSSDTSYNISVDFNKDKIINMSDLMPIALHFNSTKSSY
WP_051983568.1   ----WAGDIPKN----GIADNMINMADVFAVAKAFNT--VKGDANYNADVNFDMDATVSMQDVLIIAKHFGATPADY
WP_036940087.1   ----WAGDIAVK----GAQDNSININDVIQMAISFNS--TKGSINYNTSCDFDLDGAINIKDVVTISRHFNMTGDKY
WP_081927360.1   ----WAGDTARK----GVQDNAINMADIIEFAACFNL--TRDNANYKADIDINKDGAINMSDIIIVAKCFNKVSADY
KNY25264.1       ----WAGDVARN----GVQDNSINMADIIEFAAYFNT--TAGNAKYKDSIDINKDGSINMSDIIIVAKNFGKISSDY
KNY25033.1       ----WAGDIVKN----GVQDNSINMKDVLEIAKSFNS--ISGNALYNINADLNLDGAINMSDVIIVSKHFSKTSADY
KNY25262.1       ----WAGDMVIK----GVQDNAINMSDVIEVAKCFSS--KLGDAKYIKTDDLNEDGAINMSDVIIIARHFGKTAGEY
WP_081927368.1   ----WAGDIVIN----DKQDNAINMSDIIELAKHFNS--ISGDGKYNQNTDLNLDGTINMSDVIIIARHFNNVPGDY
WP_050753166.1   ----WAGDIKIG----GVQDDVINMTDVIQIAQGFSA--VMGDTKYKADNDLNNDNVVNMADIIVLVRHFSATPTSY
WP_081926693.1   ----LAGDIPNS----GMQDGAINMKDVVEIAKVFNT--VKGEEQYNPVCDFDMDDSINIKDVIQLAVNFGAVSEEY
WP_051983524.1   ------GDGKYKQDLDVNQDGAINMSDIIELAKGFNS--IKGDLKYSSEYDFNQDNSVNMNDVIIIAKHFNAVSASY
WP_036935133.1   ----WAGDIPND----GVQDDIINMKDIMAMAKSFNV--TYEDPSYAEYKDLNGDNAINMSDILIMAKHFNTNTGSY
WP_050753276.1   ----WAGDVPIN----GKSDGVINMTDIVEIAKCFNS--IKGAQDFNASCDFNLDDAINMSDVISIAKHFNATSGSY
WP_036942631.1   ----WAGDILIN----GKSDGAINMADVVQVAKAFSS--NKITTEFNNNCDLNCDGAINMSDIIIMAKHFNCTSNSY
KNY29155.1       ----WAGDFPAN----GVQDSIVNMADLLLMAKVFNT--TKADQNYASVYDVNSDGVINITDVIIIAKHFNATSSNY
WP_050753006.1   ----WPGDMAKN----GIQDNAINISDIIEISKVFNS--TPQDTKYKEALDINLDNAINLKDVIIIAKYFNKVSSDY
WP_051983540.1   ----WVGDMEKN----GIQDGAINMSDIIEIAKVFNS--ISGDSKYDGDSDFTKDGSINMADVIVVAKHFNATNSSY
KNY25199.1       ----WAGDIEVN----NVQDGAINMLDVIEVAKLYNA--VQNDLKYNQKADLNRDGRINLADIVLIAKNFNKTSKDY
WP_050753018.1   ----WAGDLMNN----GVQDDVINLSDFILLAKLFNS--VKGDGTYITDADINMDASINIADVIIIAKHFNETPSNY
WP_050753177.1   ----WAGDIPVN----GVGDNSINMSDVIQIAKSFNA--ITGDVNYNIDADINKDNAVNIKDVIILAKHFNASGY--
KNY25560.1       ----WAGDITKG----DVQDGAINMADVIEIAKAFNA--TSDTAKYVAEQDFNADGSINMSDILIIAKHFSMTSSNY
WP_050753616.1   ----WAGDIPVD----GVSDDAVNIKDVVEISKVFNA--VSTDSKYNADCDLNKDGSINMSDVIVVAKNFGRVSTDY
KNY25990.1       ----WAGDLNND----GTGDGVINMSDIVQLAKSFGA--VKEDAKFVSSCDLNMDSSINMSDVIIIAKHFNYTSVNY
WP_081926734.1   ----WVGDIPKN----GIQDDAINMADVISLAKVFNA--VSSDAHYESQFDLNLDGSINMSDIILLAKHFNATAANY
WP_050753443.1   ----WAGDIPVD----GIQDGAINMKDVIEIGKCFNA--TKSDSKYNSSCDLNMDNSINMSDIVIIAKHFGKTTADY
WP_050753163.1   ----WAGDIAVN----GVQDGSINMSDVIALAKGFNA--VSTDSKYIASCDLNKDNSINMSDVIILAKHFNMTTSNY
                        *           :  :*  * .     :.         :    :   *  :.: ::      *
```
