# Supplementary material for: Unique organization and unprecedented diversity of the Bacteroides (Pseudobacteroides) cellulosolvens cellulosome system
Source: Biotechnol Biofuels. 2017 Sep 7;10:211. doi: 10.1186/s13068-017-0898-6 (PMC5590126; doi:10.1186/s13068-017-0898-6)
Supplement: Supplementary file 7 — Additional file 7: Figure S6. Determination of dockerin specificity to Bacteroides cellulosolvens cell lysate components by affinity-based ELISA. The 96-well ELISA plates were coated with B. cellulosolvens cell lysate (grown on cellobiose), and various concentrations of Xyn-Docs were used to detect cohesin-dockerin interactions. Abbreviations: Doc, dockerin. [file 13068_2017_898_MOESM7_ESM.docx]

**Additional File 7:**

**Figure S6.** **Determination of dockerin specificity to *Bacteroides cellulosolvens* cell lysate by affinity-based ELISA.** The 96-well ELISA plates were coated with *B. cellulosolvens* cell lysate (grown on cellobiose), and various concentrations of Xyn-Docs were used to detect cohesin-dockerin interactions.  Abbreviations: Doc, dockerin.
