# Supplementary material for: Unique organization and unprecedented diversity of the Bacteroides (Pseudobacteroides) cellulosolvens cellulosome system
Source: Biotechnol Biofuels. 2017 Sep 7;10:211. doi: 10.1186/s13068-017-0898-6 (PMC5590126; doi:10.1186/s13068-017-0898-6)

**Additional File 8.**

**Figure S7. Determination of the inter-species interactions of *Bacteroides cellulosolvens* cell lysate by affinity-based ELISA.** Determination of the inter-species interactions of *Bacteroides cellulosolvens* cell lysate by affinity-based ELISA. The 96-well ELISA plates were coated with *B. cellulosolvens* cell lysate (grown on cellobiose) and various concentrations of Xyn-Docs from three different bacteria were used to detect cohesin-dockerin interactions.  Abbreviations: Doc, dockerin; Cc, *Clostridium clariflavum*; Rf, *Ruminococcus flavefaciens*; Ac, *Acetivibrio cellulolyticus*; GH9, Glycoside hydrolases of family 9*.* The dockerins were chosen to include the three previously defined types: Ac-GH9-Doc is a representative of type I dockerins; Cc-ScaA-Doc and Ac-ScaA-Doc represent type II dockerins; and Rf-ScaA-Doc represents type III dockerins. Here we show that *B. cellulosolvens* cell lysate is capable of crossreaction with type II dockerins from the primary scaffoldin ScaA from two different bacteria but not type I or III.


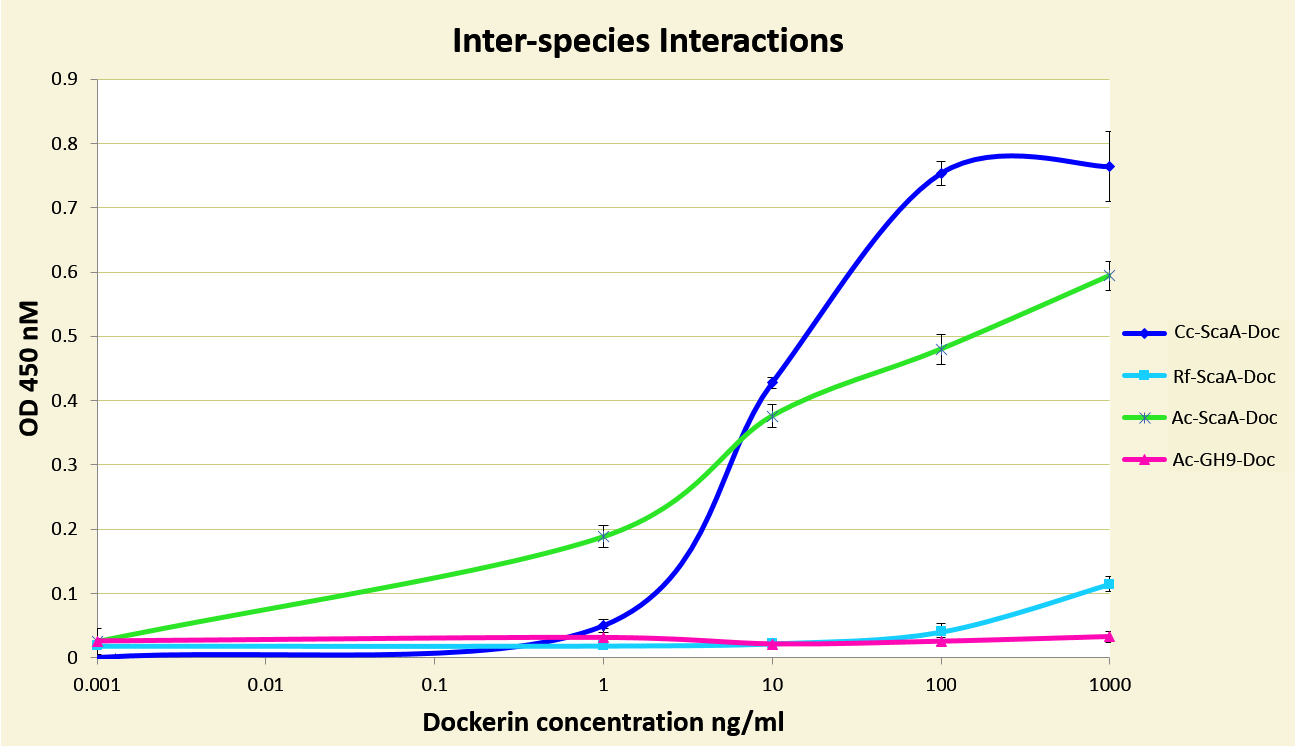

Supplement: Supplementary file 8 — Additional file 8: Figure S7. Determination of the inter-species interactions of Bacteroides cellulosolvens cell lysate by affinity-based ELISA. The 96-well ELISA plates were coated with B. cellulosolvens cell lysate (grown on cellobiose) and various concentrations of Xyn-Docs from three different bacteria were used to detect cohesin-dockerin interactions. Abbreviations: Doc, dockerin; Cc, Clostridium clariflavum; Rf, Ruminococcus flavefaciens; Ac, Acetivibrio cellulolyticus; GH9, Glycoside hydrolases of family 9. The dockerins were chosen to include the three previously defined types: Ac-GH9-Doc is a representative of type I dockerins; Cc-ScaA-Doc and Ac-ScaA-Doc represent type II dockerins; and Rf-ScaA-Doc represents type III dockerins. Here we show that B. cellulosolvens cell lysate is capable of crossreaction with type II dockerins from the primary scaffoldin ScaA from two different bacteria but not type I or III. [file 13068_2017_898_MOESM8_ESM.docx]
